# Discovery of potent tubulin inhibitors targeting the colchicine binding site via structure-based lead optimization and antitumor evaluation

**DOI:** 10.1080/14756366.2022.2155815

**Published:** 2023-01-11

**Authors:** Wei Liu, Youyou He, Zhongjie Guo, Miaomiao Wang, Xiaodong Han, Hairui Jia, Jin He, Shanshan Miao, Shengzheng Wang

**Affiliations:** a Faculty of Pharmacy, School of Food and Biological Engineering, Shaanxi University of Science and Technology, Xi’an, China; b Department of Medicinal Chemistry and Pharmaceutical Analysis, School of Pharmacy, Fourth Military Medical University, Xi’an, China

**Keywords:** Tubulin inhibitors, colchicine binding site, structural optimisation, antitumour activity

## Abstract

The colchicine binding site of tubulin is a promising target for discovering novel antitumour agents. Previously, we identified 2-aryl-4-amide-quinoline derivatives displayed moderate tubulin polymerisation inhibitory activity and broad-spectrum *in vitro* antitumour activity. In this study, structure based rational design and systematic structural optimisation were performed to obtain analogues **C1∼J2** bearing diverse substituents and scaffolds. Among them, analogue **G13** bearing a hydroxymethyl group displayed good tubulin polymerisation inhibitory activity (IC_50_  =  13.5 μM) and potent antiproliferative activity (IC_50_ values: 0.65 μM∼0.90 μM). **G13** potently inhibited the migration and invasion of MDA-MB-231 cells, and displayed potent antiangiogenic activity. It efficiently increased intracellular ROS level and decreased MMP in cancer cells, and obviously induced the fragmentation and disassembly of the microtubules network. More importantly, **G13** exhibited good *in vivo* antitumour efficacy in MDA-MB-231 xenograft model (TGI  =  38.2%; i.p., 30 mg/kg).

## GRAPHICAL ABSTRACT

**Figure:**
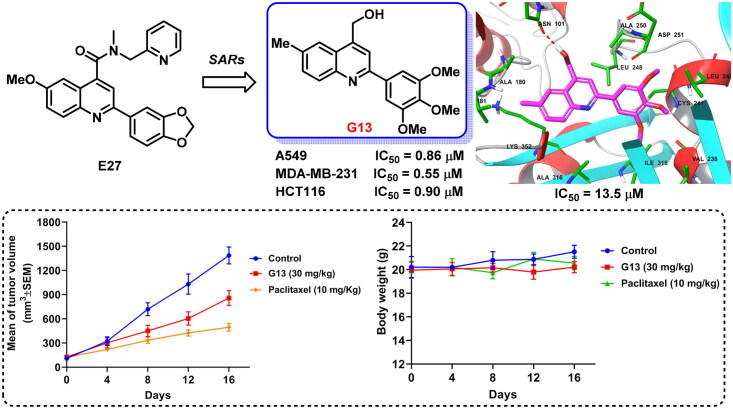

## Introduction

Microtubules play crucial roles in a wide range of cell events such as cell division, mitotic spindle formation, cell morphology and motility, and represent one of the most valuable antitumour targets for decades^1^^,^^2^. Microtubules composed by α-tubulin and *β*-tubulin heterodimers are highly dynamic cytoskeletal fibres and a major component of the cytoskeleton, which function as highways in intracellular transport of organelles, vesicles, proteins, and signalling molecules^3^^,^^4^. They show two types of non-equilibrium dynamics (i.e. dynamic instability and treadmilling), and both of them are crucial for cell division and mitosis^2^. Due to the vital roles of microtubules in cell events, microtubule targeting agents (MTAs) that regulate microtubule dynamics display excellent anti-proliferative activity. Based on the effects on microtubule dynamics, MTAs can be classified into two categories as microtubule-stabilizing and -destabilizing agents^1^^,^^5^. Microtubule-stabilizing agents (MTSAs) such as taxanes and epothilones bind to polymerised tubulin proteins and promote microtubule polymerisation^6^, while microtubule-destabilizing agents (MTDAs) such as colchicine and vinca alkaloids inhibit microtubule polymerisation^6^. Despite of the opposite effects on microtubule polymerisation, both MTSAs and MTDAs have been successfully developed as chemotherapeutic drugs and widely used in clinic for cancer therapy.

**Figure 1.**
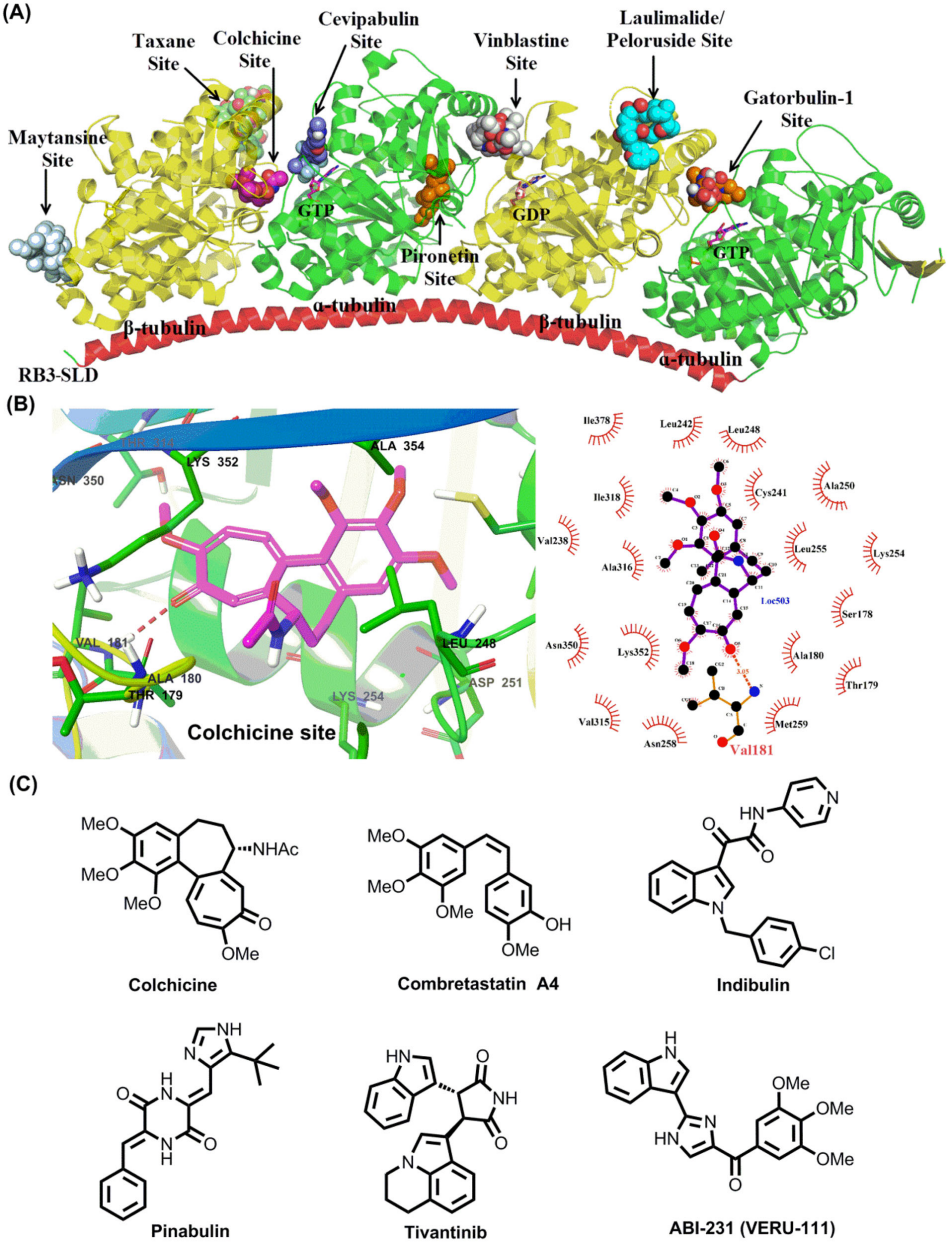
(A) Eight distinct tubulin-ligand binding sites that have been reported so far. (B) Schematic representation of the colchicine binding site in tubulin (PDB code: 4O2B). (C) Representative colchicine binding site inhibitors.

A large number of chemically diverse MTAs have been reported to bind to distinct binding sites on tubulin. The taxane, laulimalide/peloruside, colchicine, vinblastine, maytansine, gatorbulin-1, pironetin, and cevipabulin sites are the eight distinct tubulin-ligand binding sites that have been reported so far (Figure 1(A))^7–13^. Agents targeting taxane and laulimalide/peloruside binding sites on *β*-tubulin display microtubule-stabilizing effect, while agents targeting colchicine, vinblastine, maytansine, gatorbulin-1, and pironetin sites directly inhibit tubulin polymerisation. Cevipabulin binds to two spatially independent sites: vinblastine site and cevipabulin site (a novel site on α-tubulin)^11^. Agents binding to the cevipabulin site promote microtubule polymerisation and tubulin degradation^11^. Up to now, three of identified tubulin-ligand binding sites have been successfully targeted to develop cancer chemotherapeutic drugs. For instance, taxanes and vincristine antitumour drugs, binding to taxane and vinblastine sites, respectively, have achieved great success in clinic cancer treatment. The natural product maytansine (targeting maytansine site) is approved as the cytotoxic part of an antibody-drug conjugate (i.e. trastuzumab emtansine)^14^. On the contrary, agents binding to the remaining tubulin-ligand sites have not been approved so far. In addition, the approved MTAs have been acquired serious drug resistance over the long-term treatment^15^. Overexpression of transporter proteins such as P-glycoprotein (P-gp), multidrug resistance-associated protein 1 (MRP1) and MRP2, specific tubulin isotypes (especially *β*III isoform), and tubulin protein mutations are the main mechanisms that contribute to developing the drug resistance against MTAs^16–18^. Therefore, there is an urgent need to develop novel tubulin targeted chemotherapeutic drugs that are efficient to suppress the resistant phenotypes.

The colchicine binding site is located at the interface between *α* and *β*-tubulin heterodimers, and has been extensively studied for discovering novel tubulin targeted antitumour agents (Figure 1(B))^19^. Colchicine binding site inhibitors (CBSIs) are reported to display excellent anti-proliferative activity with IC_50_ values at nanomolar level, and valuable antitumour properties such as reducing cellular motility, impeding protein assembly, and exerting antiangiogenic effect^17^. Moreover, extensive preclinical and clinical studies have strongly suggested CBSIs are effective in suppressing the multi-drug resistance mediated by the overexpression of *β*III tubulin or transporter proteins such as P-gp, MRP1 and MRP2^16–18^. Colchicine, an alkaloid isolated from the plant *Colchicum autumnale*, displays excellent submicromolar antiproliferative activities with wide-scope mechanisms of action^20^^,^^21^. Unfortunately, it has a narrow therapeutic index and induces systemic toxicities and multi-organ dysfunction at high doses that hamper the therapeutic application in cancer treatment^22^^,^^23^. In addition to colchicine and its derivatives, numerous structurally diverse CBSIs have been reported so far (Figure 1(C))^1^^,^^5^^,^^16^^,^^17^^,^^24–30^. Several inhibitors are actively undergoing clinical trials for the therapy of various solid tumours. For instance, ABI-231 (Sabizabulin, VERU-111) is a potent, orally bioavailable CBSI that is currently undergoing several clinical trials for the therapy of metastatic castration-resistant prostate cancer (Phase 3)^31^ and metastatic breast cancer (Phase 2)^32^. Based on the results of phase 1 b/2, oral 63 mg daily dosing of ABI-231 is well tolerated and associated with significant and durable objective tumour responses in patients^33^. Taken together, CBSIs display encouragingly *in-vitro* and *-vivo* antiproliferative activity with wide-scope molecular mechanisms, which are promising to surmount drug resistance faced by the available microtubule-targeting drugs.

**Figure 2.**
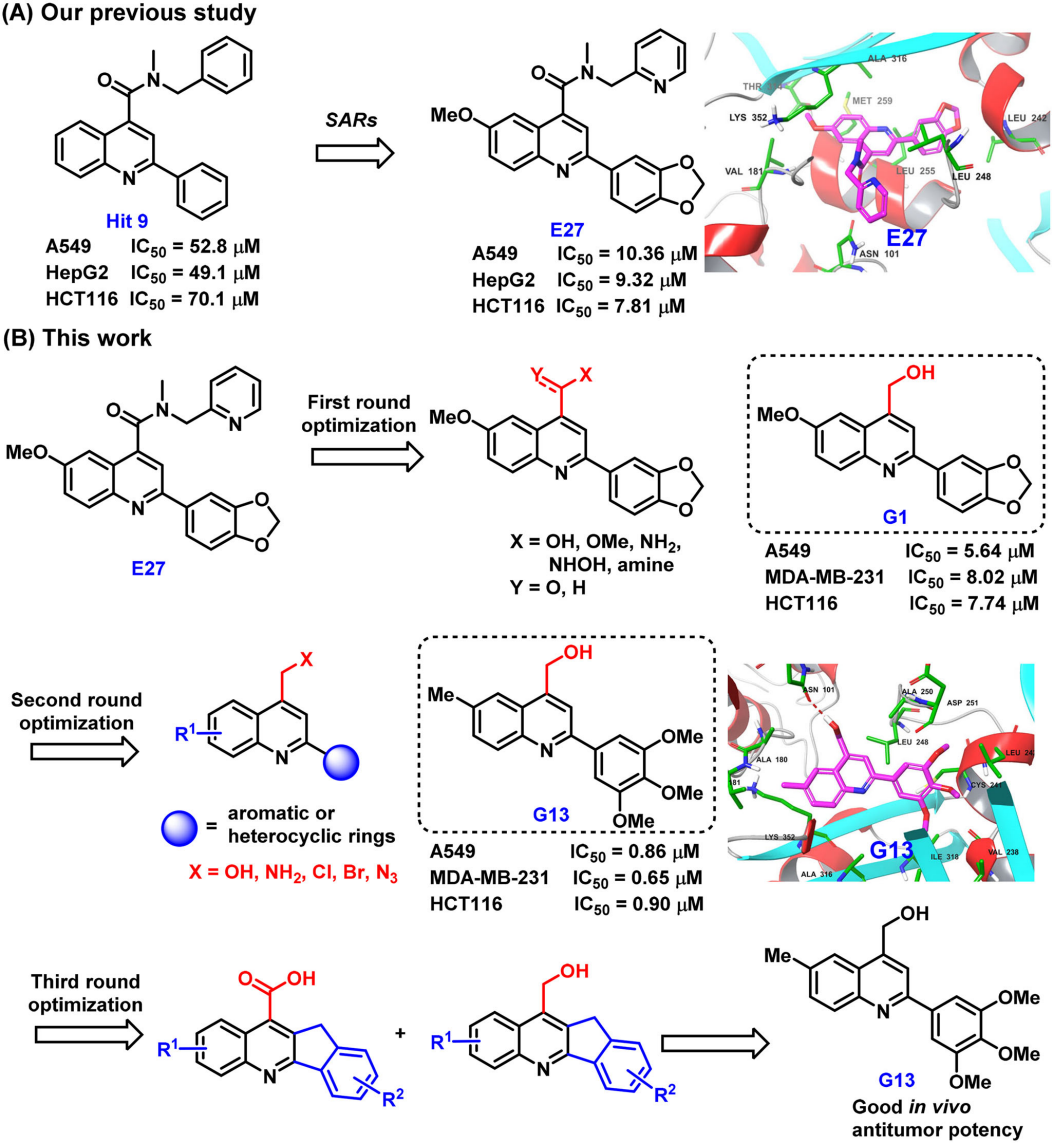
(A) Our previous study identified 2-aryl-4-amide-quinoline derivatives as the novel colchicine binding site inhibitors. (B) Rational drug design and structural optimisation were performed to identify analogue **G13** displaying potent antitumour activity.

In our previous study, structure based virtual screening against colchicine binding site was performed to indentify 2-aryl-4-amide-quinoline hit **9** (Figure 2(A)) displayed moderate tubulin polymerisation inhibitory activity (IC_50_  =  25.3 μM) and broad-spectrum *in vitro* antiproliferative activity (IC_50_ values: 49.1 μM∼70.1 μM)^34^. After the preliminary structural optimisation, analogue **E27** (Figure 2(A)) was identified to display improved enzymatic inhibition activity (IC_50_  =  16.1 μM) and antitumour activity (IC_50_ values: 7.81 μM∼10.36 μM). Guided by the binding mode of **E27** with tubulin, we envisioned that the 4-amide long side chain could be truncated and optimised to improve the molecular interactions to the binding site (Figure 2(A)). Therefore, structure based rational design and structural optimisation were performed in this study (Figure 2(B)). A series of novel analogues with diverse substituents and scaffolds were synthesised. Biological evaluation identified analogue **G13** (Figure 2(B)) bearing 4-hydroxymethyl and 2–(3′,4′,5′-3OMe-phenyl) groups displayed improved enzymatic inhibition activity (IC_50_  =  13.5 μM) and potent *in vitro* antitumour potency (IC_50_: 0.65 μM∼0.90 μM). More strikingly, **G13** exhibited good *in vivo* antitumour potency in MDA-MB-231 xenograft model with low toxicity (TGI  =  38.2%; i.p., 30 mg/kg). Altogether, this study provided a valuable lead compound for developing novel tubulin-targeted antitumour agents.

## Results and discussion

### Structure based rational design

**Scheme 1.**
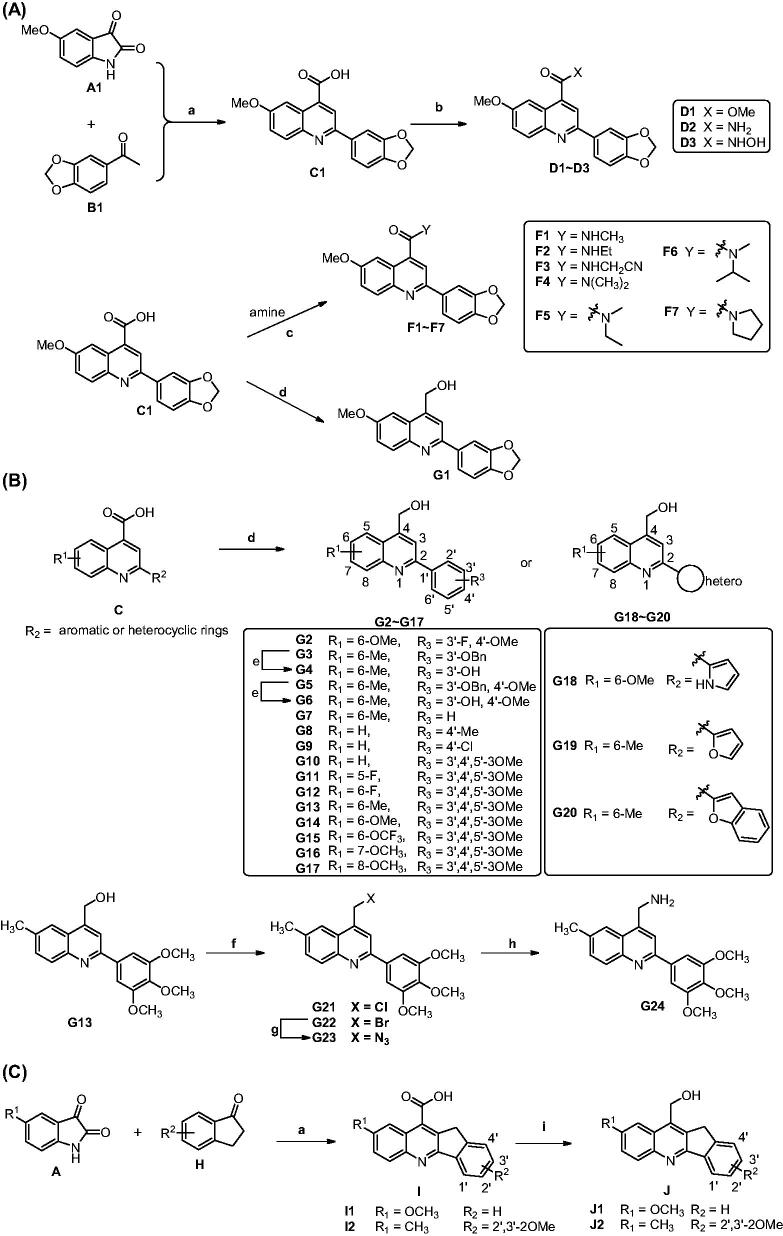
Reagents and conditions: (a) 33% KOH, EtOH, 80 °C ∼ 100 °C, 24 h; HCl, H_2_O, rt, pH = 2. (b) For **D1** and **D2**: SOCl_2_, DCM, reflux 6 h, then methanol or ammonia added; For **D3**: CDI, THF, NH_2_OH·HCl, rt. (c) Amine, EDC, DMAP, DCM, rt. (d) LiAlH_4_, THF, 0 °C. (e) For **G21**: SOCl_2_, DCM, 0 °C; For **G22**: NBS, PPh_3_, DCM, rt. (g) DMF, NaN_3_, rt. (h) THF/H_2_O, PPh_3_. (i) NaBH_4_/BF_3_·OEt_2_, THF, rt.

The predicted binding mode of **E27** with tubulin revealed the hydrophobic amide side chain of **E27** could be truncated and optimised to form a preferable interaction with tubulin (Figure 2(A)). Therefore, we firstly focussed on the optimisation of the hydrophobic amide side chain of **E27** to prepare truncated methyl ester derivative **D1**, amide derivatives **D2** and **F1∼F7**, hydroxamic acid derivative **D3**, and hydroxymethyl derivative **G1** in first round optimisation (Scheme 1(A)). Structure activity relationship studies (SARs) revealed compound **G1** bearing a hydroxymethyl group displayed the best tubulin inhibition activity and greatly improved *in vitro* antitumour activity. To account the essential role of the hydroxymethyl group, molecular docking was performed to visualise the binding mode of **G1** with tubulin. The hydroxyl group of **G1** formed a hydrogen bond with residue Asn101 that greatly improved the binding affinity of **G1** with tubulin. Therefore, the hydroxymethyl group was retained in subsequent optimisation process. In second round optimisation, we investigated the effects of diverse R_1_ and R_2_ groups on antitumour activity. We varied the attached position and electronic properties of substituents to synthesise analogues **G2∼G20** (Scheme 1(B)). Moreover, the hydroxyl group of **G13** was transformed to obtain halogen derivatives **G21** and **G22**, azide derivative **G23** and amino derivative **G24** (Scheme 1(B)). To explore the effect of rotatable substituents at C2 position of the quinoline scaffold on activity, analogues **I1∼J2** containing a non-rotatable restrained rigid planar structure were synthesised in third round optimisation (Scheme 1(C)).

### Chemical synthesis of analogues C1∼J2

The synthetic procedure was depicted in Scheme 1. Accordingly to our previous report, 5-methoxyisatin (**A1**) and 3′,4′-(methylenedioxy)acetophenone (**B1**) were dissolved in ethanol solvent and refluxed to obtain the intermediate **C1**^34^. In DCM solvent, intermediate **C1** was refluxed with SOCl_2_ to afford acid chloride derivative. Then the solvent and excess SOCl_2_ were evaporated under reduced pressure and the residue was reacted with methanol or ammonia in DCM at room temperature to afford methyl ester derivative **D1** and primary amide derivative **D2**, respectively. In the presence of *N*,*N'*-carbonyldiimidazole (CDI), **C1** was reacted with hydroxylamine hydrochloride to afford hydroxamic acid derivative **D3**. Using EDC/DMAP as the condensation reagents, **C1** was reacted with various primary or secondary amine to afford amide derivatives **F1∼F7**. Moreover, the carboxyl group of **C1** was reduced by LiAlH_4_ in THF at 0 °C to afford analogue **G1** bearing a hydroxymethyl group. The synthetic route for analogues **G2∼G20** was similar to that of **G1** (Scheme 1(B)). In the presence of SOCl_2_ or PPh3/NBS, the hydroxyl group of **G13** was transformed to chlorine (**G21**) and bromine (**G22**). Moreover, the bromine atom of **G22** was reacted with NaN_3_ to afford azide derivative **G23**, which further reduced in the presence of PPh_3_ to obtain amino derivative **G24**. Furthermore, analogues **I1∼J2** bearing a restrained rigid plane structure were designed and synthesised (Scheme 1(C)). In the presence of KOH, substituted isatin was reacted with 2,3-dihydro-1*H*-inden-1-one to afford indeno[1,2-b]quinoline analogues **I1∼I2**. Moreover, the carboxyl group of **I1∼I2** was reduced by NaBH_4_/BF_3_·OEt_2_ in THF at room temperature to afford analogues **J1∼J2** bearing a hydroxymethyl group.

### In vitro antitumour activity

CCK-8 method was used to evaluate *in vitro* antiproliferative activity of analogues **C1∼J2** against three cancer cell lines including HCT116 (colon cancer cell), A549 (lung cancer cell) and MDA-MB-231 (triple-negative breast cancer cell). Cisplatin and colchicine were used for comparison. As shown in Table 1, compounds **C1** and **D1**, bearing a carboxyl and methyl ester group, respectively, were inactive against three cancer cell lines (IC_50_ > 50 μM). Compound **D2** bearing a primary amide group displayed weak activity against A549 (IC_50_  =  46.56 μM). In contrast, compound **D3** bearing a hydroxamic acid group displayed broad-spectrum and moderate antitumour activity (IC_50_: 35.25 μM∼38.26 μM). We further synthesised analogues **F1∼F7** containing a secondary or tertiary amide group. Among them, **F5** and **F6** bearing an asymmetric tertiary amide group displayed the best anti-proliferative activity (IC_50_: 8.00 μM∼30.52 μM). More strikingly, compound **G1** bearing a hydroxymethyl group displayed greatly improved antitumour activity with IC_50_ values ranging from 5.64 μM to 8.02 μM, indicating the hydroxyl group might form an essential interaction with surrounding residues. Based on the above SARs, we further synthesised analogues **G2∼G20** bearing the hydroxymethyl group. SARs revealed the attached position and properties of R_1_ and R_2_ substituents had significant effects on antitumour activity. Among the analogues, compounds **G13∼G15**, bearing a 3′,4′,5′-3OMe phenyl group at C2 position of quinoline scaffold, displayed the best antitumour activity with IC_50_ values lower than 5 μM. For instance, IC_50_ values of **G13** against HCT116, A549 and MDA-MB-231 were 0.90 μM, 0.86 μM and 0.65 μM, respectively, which were more potent than that of CDDP. Compounds **G18∼G20** bearing a heterocyclic substituent also displayed moderate to good antiproliferative activity. For instance, IC_50_ values for **G18** containing a pyrrole group are ranging from 8.19 μM to 12.71 μM. Furthermore, the hydroxyl group of **G13** was transformed to chlorine (**G21**), bromine (**G22**), azide (**G23**), and amino (**G24**). These analogues displayed broad-spectrum and moderate to good antitumour activity. Analogues **I1∼J2** bearing a restrained rigid plane structure displayed decreased antitumour activity, indicating the restrained rigid plane structure was not favourable for antitumour activity. Among the derivatives, **G13** displayed the best antitumour activity. The cytotoxicity of **G13** on human embryonic kidney cell 293 T was evaluated using CCK-8 method. It displayed moderate inhibitory activity against 293 T (IC_50_  =  18.12 μM). Taken together, **G13** was selected for further biological evaluation.

**Table 1.** *In vitro* antitumour activity of compounds **C1∼J2** (IC_50_, μM).

Compounds	HCT116	A549	MDA-MB-231
**C1**	>50	>50	>50
**D1**	>50	>50	>50
**D2**	>50	46.56 ± 1.23	>50
**D3**	38.16 ± 0.11	35.25 ± 0.14	38.26 ± 1.87
**F1**	>50	41.57 ± 0.60	29.01 ± 0.48
**F2**	>50	47.22 ± 0.96	>50
**F3**	>50	46.07 ± 0.47	>50
**F4**	20.12 ± 0.15	21.62 ± 0.20	52.28 ± 0.21
**F5**	19.47 ± 0.40	10.76 ± 0.08	25.10 ± 0.42
**F6**	16.50 ± 0.28	8.00 ± 0.65	30.51 ± 1.61
**F7**	>50	>50	>50
**G1**	7.74 ± 0.23	5.64 ± 0.23	8.02 ± 0.15
**G2**	28.42 ± 1.37	27.29 ± 0.29	21.52 ± 0.65
**G3**	>50	>50	>50
**G4**	25.49 ± 0.23	12.36 ± 0.23	12.90 ± 0.37
**G5**	>50	46.11 ± 2.14	49.46
**G6**	>50	23.90 ± 0.38	33.42 ± 0.46
**G7**	43.46 ± 2.23	24.81 ± 0.20	18.97 ± 0.47
**G8**	>50	49.64 ± 0.25	48.35 ± 1.12
**G9**	>50	29.67 ± 0.15	35.89 ± 0.85
**G10**	>50	35.26 ± 0.12	47.52 ± 0.16
**G11**	>50	>50	>50
**G12**	26.68 ± 0.25	39.58 ± 0.11	30.56 ± 0.21
**G13**	0.90 ± 0.05	0.86 ± 0.12	0.65 ± 0.10
**G14**	3.49 ± 0.05	2.3 ± 0.07	4.01 ± 0.10
**G15**	1.20 ± 0.12	1.96 ± 0.12	2.68 ± 0.11
**G16**	>50	46.25 ± 0.23	>50
**G17**	25.22 ± 1.29	20.15 ± 0.12	36.32 ± 1.74
**G18**	11.84 ± 0.25	8.19 ± 0.12	12.71 ± 0.13
**G19**	41.05 ± 0.92	20.97 ± 0.13	27.03 ± 0.41
**G20**	5.7 ± 0.09	11.91 ± 0.11	11.14 ± 0.33
**G21**	30.26 ± 0.11	25.29 ± 0.08	25.60 ± 0.29
**G22**	25.32 ± 0.27	40.65 ± 0.26	16.25 ± 0.45
**G23**	20.65 ± 0.13	44.32 ± 0.25	14.52 ± 0.13
**G24**	18.90 ± 0.22	24.81 ± 0.21	13.89 ± 0.19
**I1**	>50	>50	>50
**I2**	>50	52.86 ± 0.09	>50
**J1**	>50	49.91 ± 0.07	>50
**J2**	45.29 ± 0.15	51.77 ± 0.66	30.45 ± 0.43
**Colchicine**	0.079 ± 0.13	0.12 ± 0.14	0.35 ± 0.16
**Cisplatin**	8.43 ± 0.30	5.58 ± 0.11	15.31 ± 0.14

### Structure–activity relationships for analogues C1∼J2

**Figure 3.**
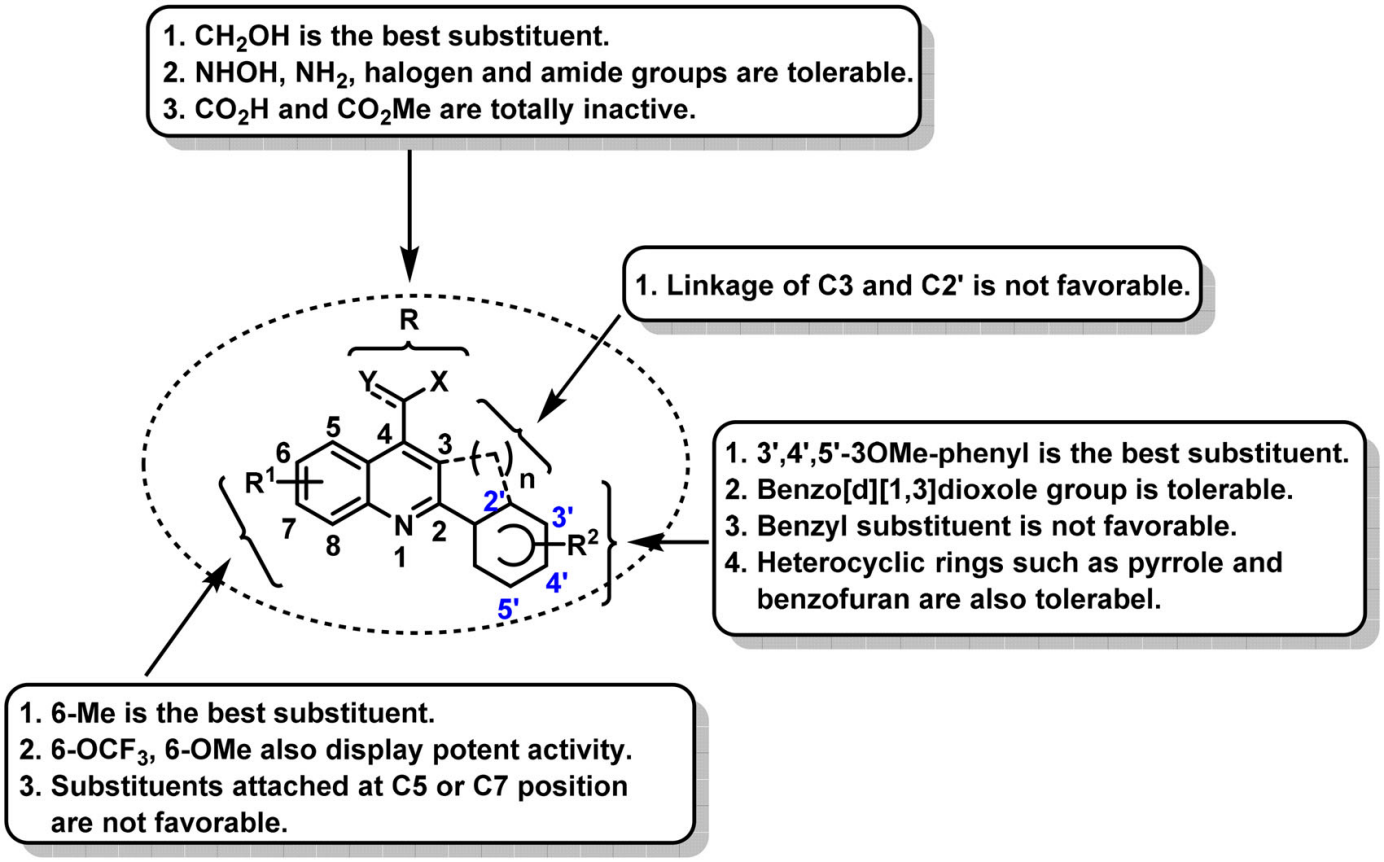
Summary of SARs for analogues **C1∼J2**.

Summary of SARs for analogues **C1∼J2** was depicted in Figure 3. For R group at C4 position of the quinoline scaffold, the hydroxymethyl substituent significantly improved the antitumour activity. The hydroxamic acid, amino, amide, or halogen group was also tolerable, but displayed less potent activity. In contrast, when the R group was a carboxylic acid or methyl ester group, compounds were inactive. For R_1_ and R_2_ groups, we observed the attachment position and electronic properties of substituents had significant effects on activity. For R_1_ group, substituents attached at C6 position was more favourable than C5 or C7 position. 6-Methyl was the best substituent and greatly improved the activity. 6-Trifluoromethoxy and 6-methoxy were also favourable for activity. For R_2_ group, the best substituent was 3′,4′,5′-3OMe phenyl, which is the most common structure feature in numerous potent CBSIs such as colchicine, combretastatin A4 and ABI-231. Benzo[d][1,3]dioxole moiety was also tolerable, but displayed less potent activity. Larger groups like benzyl substituent were detrimental to activity. Furthermore, analogues **G18∼G20** bearing a heterocyclic substituent displayed moderate to good antiproliferative activity, indicating the heterocyclic substituents were also tolerable. Four rings confused analogues **I1∼J2** displayed moderate to weak antitumour activity, indicating the linkage of C3 and C2’ by sp3 carbon atom was not favourable for activity.

### Tubulin inhibition activity and the binding mode of G13 with tubulin

**Figure 4.**
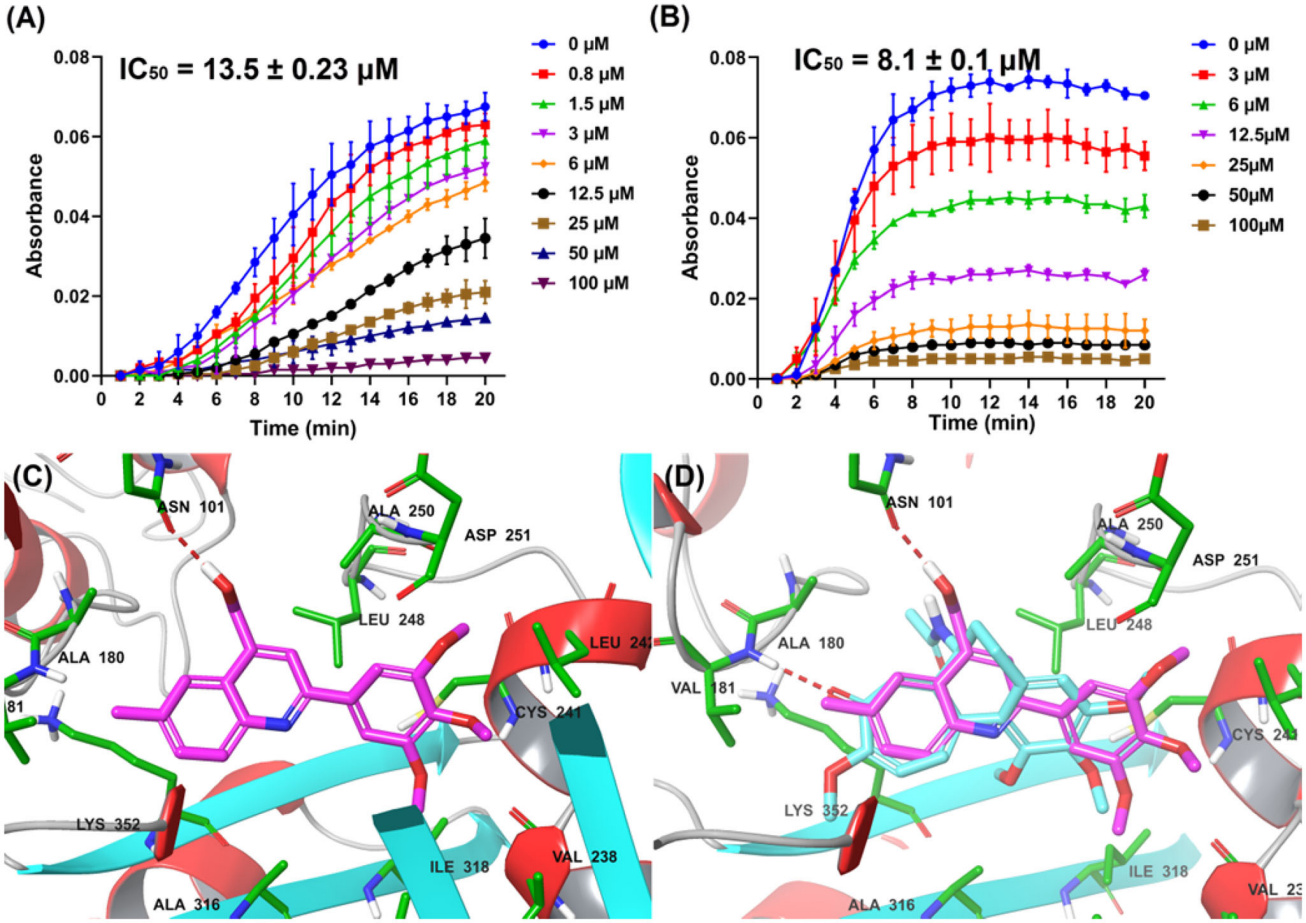
(A) Tubulin polymerisation inhibitory activity of **G13**. (B) Tubulin polymerisation inhibitory activity of colchicine. (C) The binding mode of **G13** with tubulin. (D) The overlapped binding modes of **G13** (pink) and colchicine (blue).

The tubulin inhibition activity was performed according to our previous reports^34^^,^^35^. Colchicine was used as the positive control. As depicted in Figure 4(A), compound **G13** displayed the potent tubulin polymerisation inhibitory activity with an IC_50_ value of 13.5 μM. In contrast, colchicine displayed a more potent enzymatic inhibition activity (IC_50_  =  8.1 μM), which was consistent with the more potent cytotoxicity (Figure 4(B)).

To clarify the binding mode of **G13** with tubulin, molecular docking using Glide within Schrodinger software was performed according to our previous report^34^. As depicted in Figure 4(C), **G13** was observed to be well-fitted into the colchicine binding site. The hydroxymethyl group of **G13** formed a hydrogen bond with residue Asn101, thus greatly improving the binding affinity and accounting the essential role for antitumour activity. 3′,4′,5′-3OMe-phenyl moiety of **G13** was well overlapped with the 3OMe-phenyl moiety of colchicine (Figure 4(D)), and formed mainly hydrophobic interactions with surrounding residues including Leu242, Ile318, Val238, and Ile378. 6-Methyl-quinoline moiety of **G13** was well overlapped with the tropone scaffold of colchicine and formed additional hydrophobic interactions with residues Ala180, Val181 and Val182, thus improving the activity.

### G13 potently suppressed the migration and invasion of MDA-MB-231 cells

**Figure 5.**
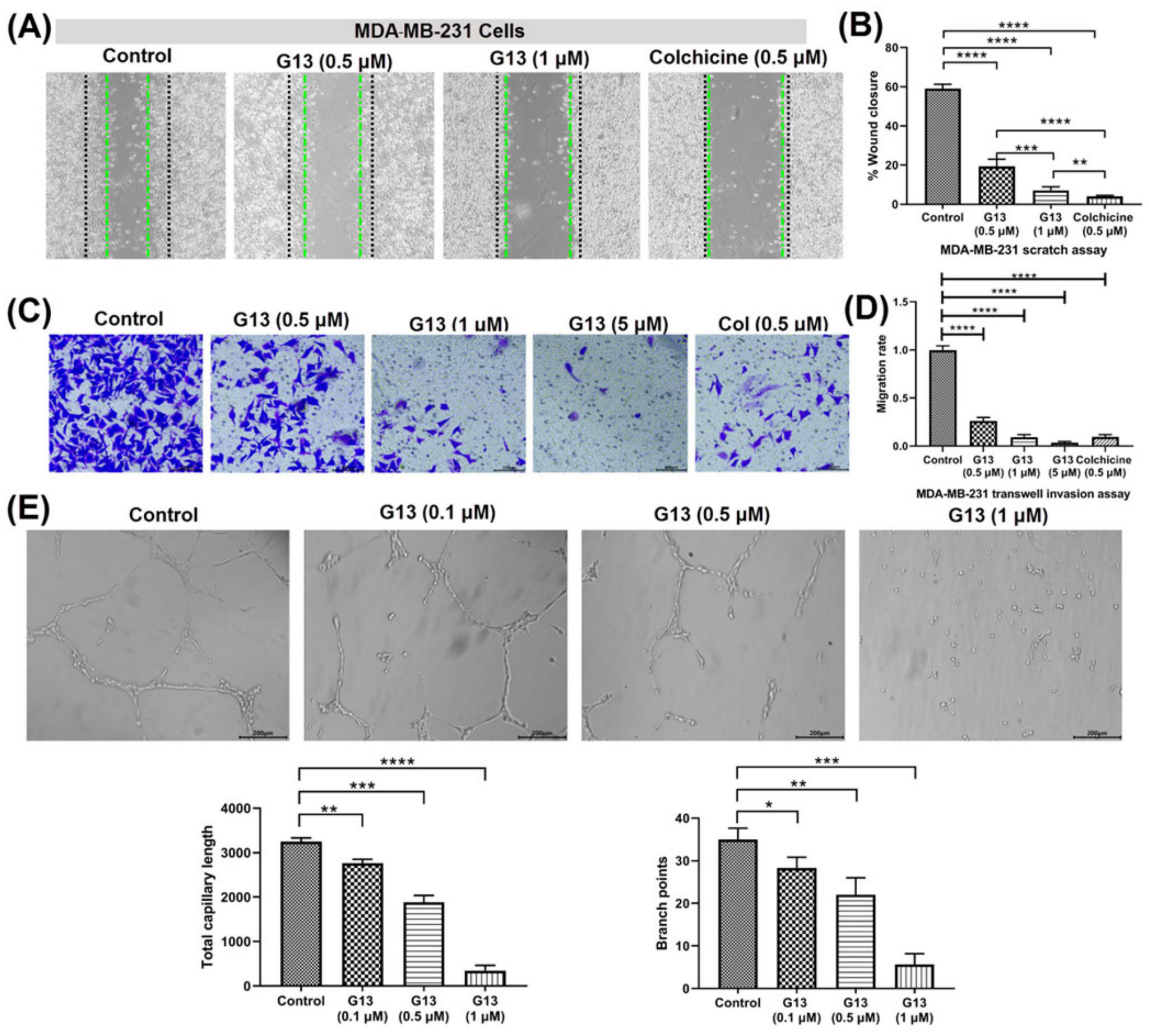
(A) The wound healing assay was performed using MDA-MB-231 cells treated with **G13** (0.5 μM or 1 μM) or colchicine (0.5 μM) for 24 h. The black and green dash lines indicated the edge of the scratch at 0 h and 24 h, respectively. (B) The wound closure area was measured by ImageJ software. (C) The transwell invasion assay was performed using MDA-MB-231 cells treated with **G13** (0.5 μM, 1 μM or 5 μM) or colchicine (0.5 μM) for 24 h. (D) The relative migration rate was calculated by comparing with the control group. (E) The tube formation assay was performed using HUVECs treated with **G13** (0.1 μM, 0.5 μM or 1 μM) for 6 h, then capillary-like networks were captured by the inverted microscope. Scale bar = 200 μm. The results are presented as the mean ± standard deviation. *p < .05, **p < .01, ***p < .001, and ****p < .0001, determined with unpaired *t* test.

The migration and invasion of cancer cells play an essential role in cancer metastasis, which is the most common cause for patients death. For investigating the effects of **G13** on anti-migratory activity against cancer cells, wound healing assay was performed using MDA-MB-231 cells. As depicted in Figure 5(A), **G13** potently suppressed the migration of MDA-MB-231 cells in a concentration dependant manner. Cells treated with **G13** at 0.5 μM or 1 μM displayed obvious declined migration capability with the wound closure (%) of 19.3% and 7.1%, respectively. Colchicine at 0.5 μM displayed potent anti-migratory activity (4.0%), which was consistent with the potent *in vitro* antitumour activity.

Transwell cell invasion assay is commonly used to measure cell chemotaxis and the invasion ability of cells through extracellular matrix, which are always found in cancer metastasis or embryonic development^36^. For investigating the effects of **G13** on anti-metastasis activity against cancer cells, transwell cell invasion assay was performed using MDA-MB-231 cells. As shown in Figure 5(C), **G13** potently decreased the invasion of MDA-MB-231 cells in a dose dependant manner. At 1 μM, **G13** displayed a comparable inhibitory effect with that of colchicine at 0.5 μM. More strikingly, **G13** at 5 μM almost totally inhibited cell invasion, which was superior to that of colchicine at 0.5 μM.

### G13 displayed potent antiangiogenic activity

CBSIs have been reported to display potent antiangiogenic effect, which is important to prevent the tumour recurrence and metastasis^37^. To investigate the effects of **G13** on the formation of new blood vessels, tube formation assay using human umbilical vein endothelial cells (HUVECs) was performed. As shown in Figure 5(E), HUVECs in the control group formed the intact capillary-like networks. After treating with **G13** at 1 μM for 6 h, most HUVECs were observed to form spherical and small clusters. In contrast, **G13** showed weak antiangiogenic activity at the concentration of 0.1 μM. The total capillary length and branch points were analysed by Angiogenesis Analyser installed in ImageJ software. This assay indicated **G13** displayed obvious antiangiogenic effect in a concentration-dependent manner, which has promising applications in discovery of novel drugs that prevent the tumour recurrence and metastasis.

### G13 induced the remarkable ultrastructural alterations of intercellular organelles

**Figure 6.**
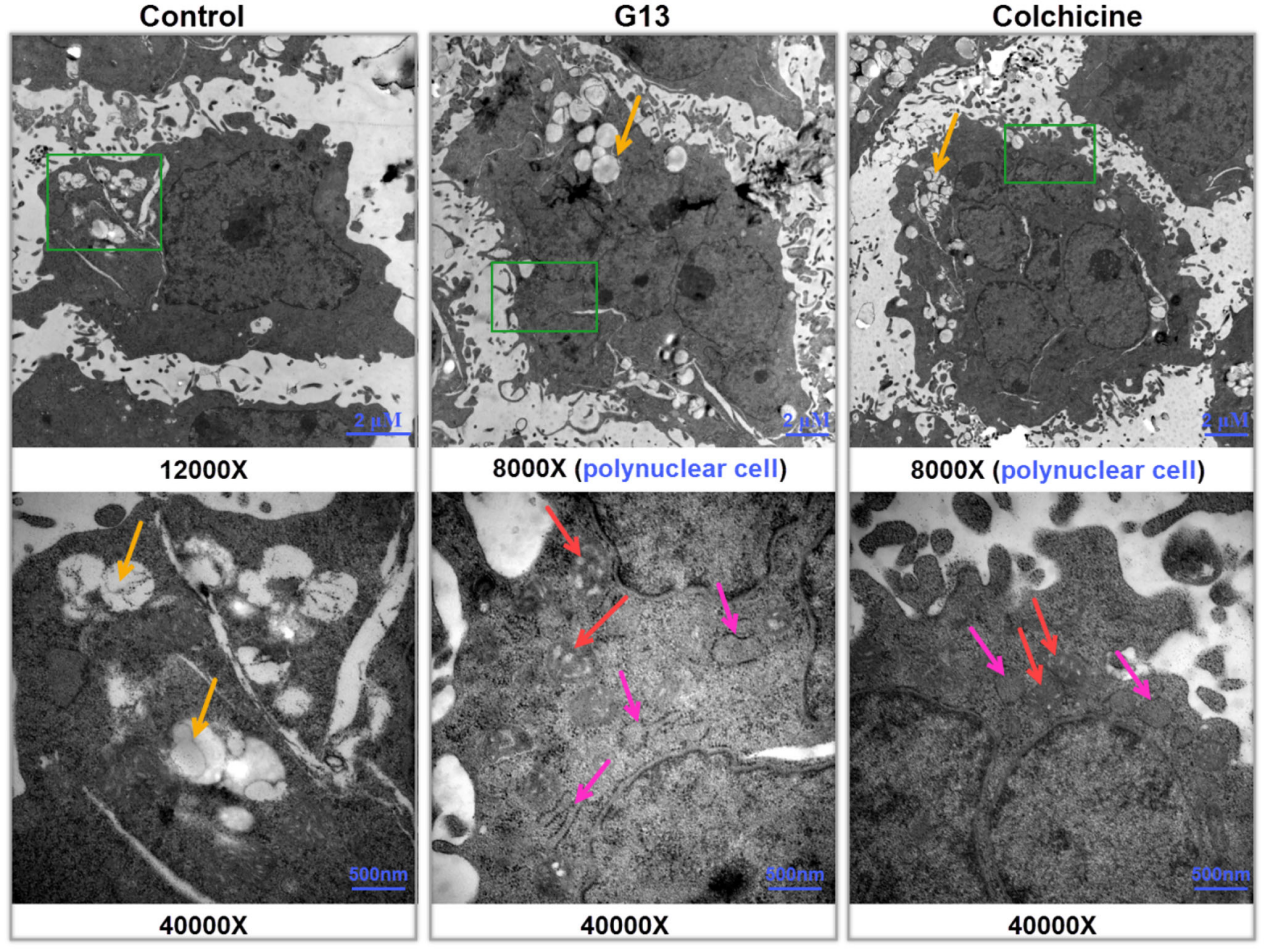
Transmission electron micrographs of MDA-MB-231 cells treated with 0.1% DMSO (control group), **G13** (5 μM) or colchicine (5 μM) for 48 h. The green area in the top micrographs was enlarged below the micrographs. The yellow arrows illustrated a lot of fat droplets in cells. The red arrows illustrated the ultrastructural alterations of mitochondria. The pink arrows indicated the endoplasmic reticulum became dilation. Cells treated with **G13** or colchicine resulted in the formation of polynuclear cells.

Transmission electron microscope (TEM) has been widely used to directly visualise the ultrastructural alterations of the intercellular organelles at high magnification, thus representing a valuable tool to explore the molecular mechanism^38^. To investigate the effect of **G13** on intercellular organelles, MDA-MB-231 cells were treated with 0.1% DMSO (control group), **G13** (5 μM), or colchicine (5 μM) for 48 h, then collected and captured using TEM. As depicted in Figure 6, cells in control group displayed an intact nucleus, normal cellular morphology, and undamaged intercellular organelles. Moreover, cells showed a lot of apparent fat droplets (yellow arrows). In contrast, cells treated with **G13** showed the severe damage of mitochondria as seen by the swollen structure and the enlarged cristae spaces (red arrows). In addition, the rough endoplasmic reticulum became dilation (pink arrows). Similar alterations of mitochondria and endoplasmic reticulum were observed in colchicine treated cells. More strikingly, treatment with **G13** or colchicine led to the formation of many polyploidy cancer cells (Figure 6). It has been reported that tubulin inhibitors can induce mitotic arrest and result in formation of polynuclear cells, which are generally considered to be non-dividing cells or at the edge of apoptosis^39^^,^^40^. Taken together, the TEM results indicated **G13** induced the remarkable alterations of the mitochondria and endoplasmic reticulum, blocked mitotic to form polynuclear cells, and eventually triggered apoptosis.

### G13 caused severe damage to the microtubule network in MDA-MB-231 cells

**Figure 7.**
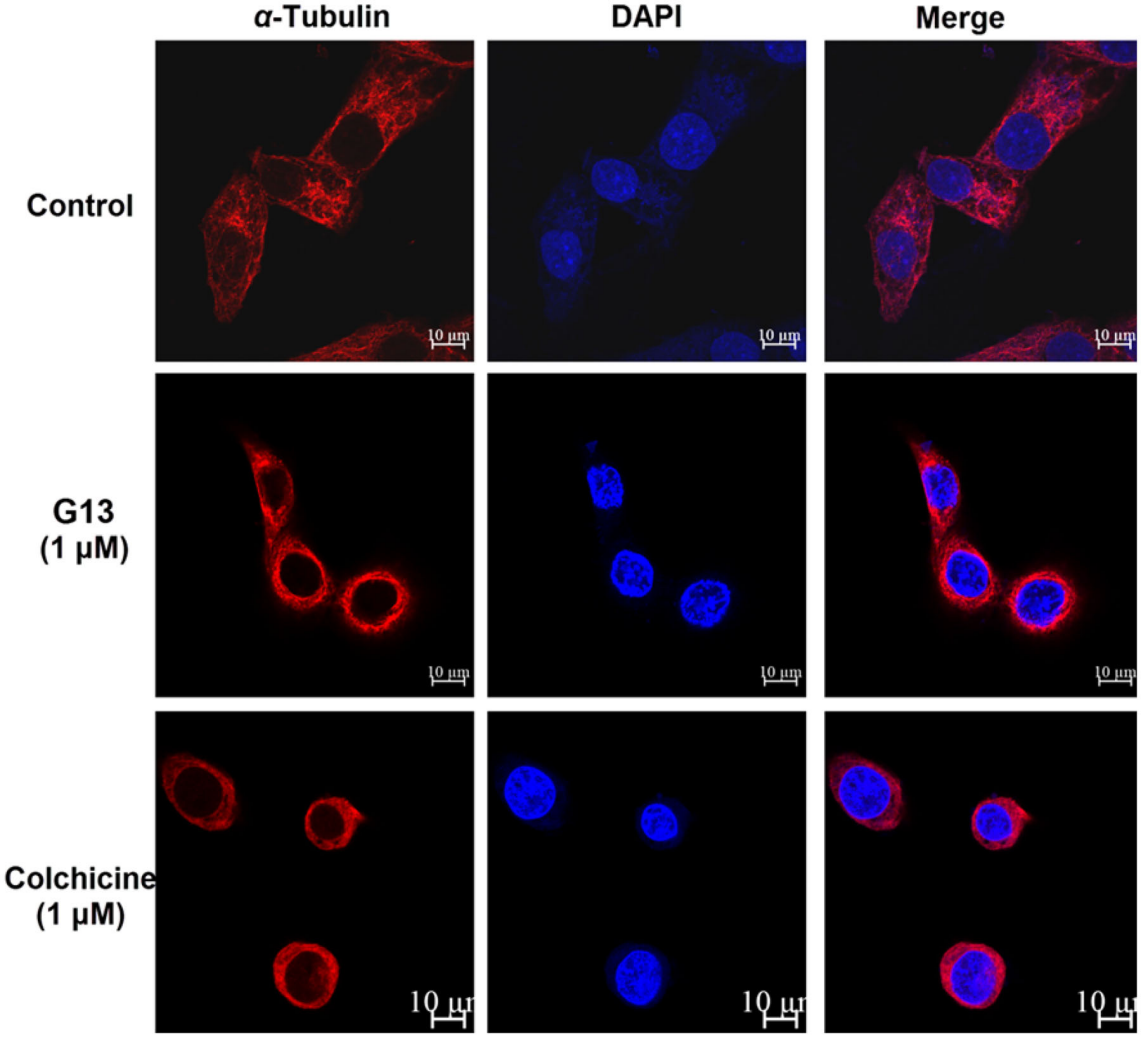
MDA-MB-231 cells were treated with 0.1% DMSO (control group), **G13** (1 μM) or colchicine (1 μM) for 24 h, then the change of microtubule networks was visualised using confocal immunofluorescent microscopy. Scale bar = 10 μm.

CBSIs interact with tubulin monomers and prevent their polymerisation into microtubule network. Due to the dynamic character of microtubule network, cells treated with CBSIs have been reported to display the fragmentation and disassembly of the microtubule network^1^^,^^41^. For visualising the effect of **G13** on microtubule network, immunofluorescent assay was performed according to our previous report^34^. Alexa-594 fluorescent labelled *α*-tubulin antibodies fluoresce red to visualise the microtubule network, and DAPI fluoresces blue to visualise the nucleus. As shown in Figure 7, MDA-MB-231 cells treated with 0.1% DMSO (control group) showed an intact organised microtubule network extending throughout all cells. In contrast, cells treated with **G13** at 1 μM showed the fragmentation and disassembly of the microtubule network indicating a serious damage was induced. Similarly, cells treated with colchicine at 1 μM showed severe damage of the microtubule network. This assay revealed **G13** potently prevented the polymerisation of tubulin monomers and resulted in the depolymerisation and disassembly of the microtubule network.

### G13 potently induced apoptosis in MDA-MB-231 cells

**Figure 8.**
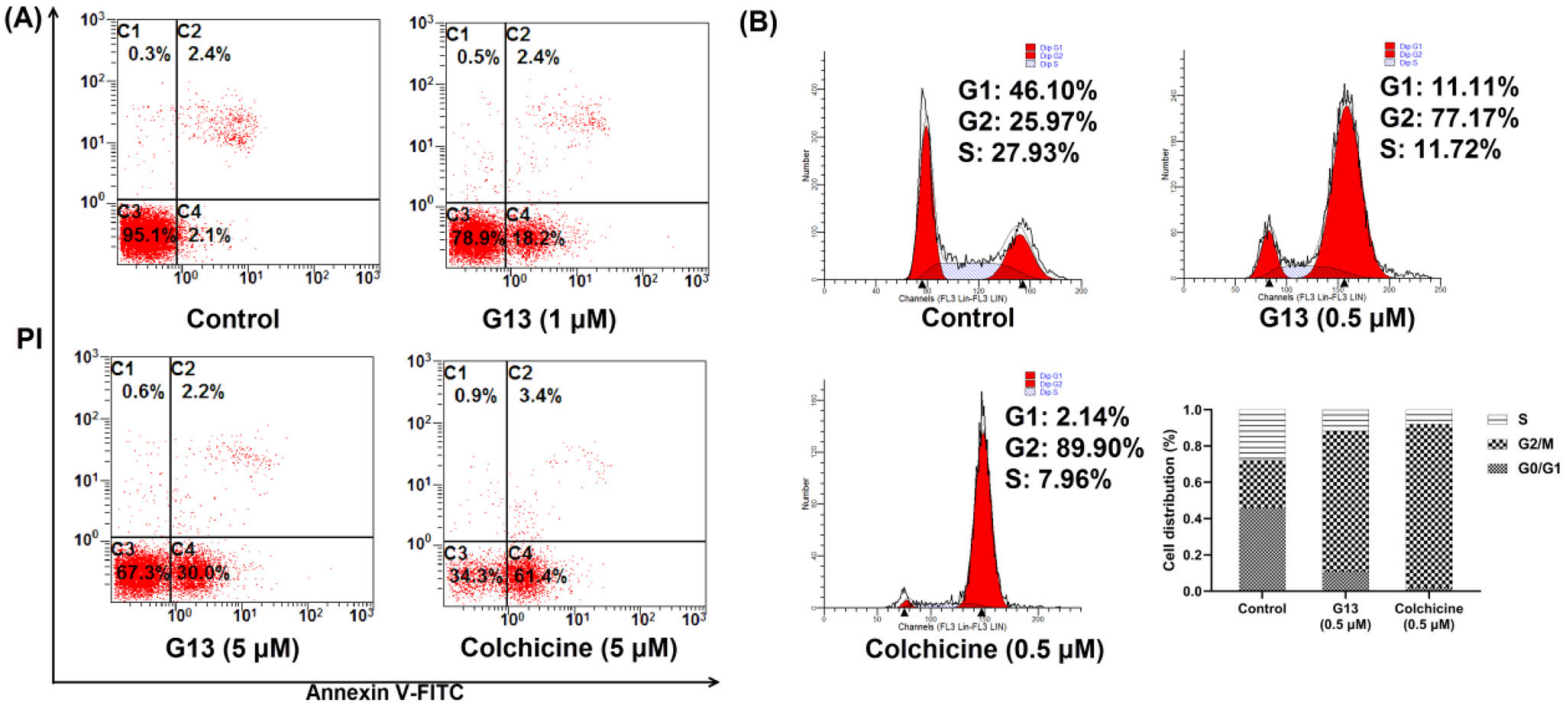
Induction of apoptosis and cell-cycle arrest by **G13** in MDA-MB-231 cells. (A) MDA-MB-231 cells were treated with 0.1% DMSO (control group), **G13** (1 μM or 5 μM) or colchicine (5 μM) for 24 h, stained with annexin V-FITC/PI apoptosis detection kit and analysed by flow cytometry. (B) Cell cycle analysis after treatment with 0.1% DMSO (control group), **G13** (0.5 μM) or colchicine (0.5 μM) for 24 h. The data are representative of two independent experiments.

CBSIs bind tightly with tubulin monomers to perturb microtubule dynamics, thus leading to cell cycle arrest, apoptosis and ultimately cell death. To evaluate the effect of **G13** on cell apoptosis, MDA-MB-231 cells were treated and detected by flow cytometry. As depicted in Figure 8(A), both **G13** and colchicine induced a significant apoptosis in MDA-MB-231 cells. After treating with **G13** at 1 μM or 5 μM for 24 h, the apoptotic rates were 20.6% and 32.2%, respectively. Colchicine at 5 μM induced a higher apoptotic rate (64.4%), which was consistent with the more potent cytotoxicity. In contrast, cells treated with 0.1% DMSO (control group) displayed a low apoptotic rate (4.5%). This assay revealed **G13** potently induced cell apoptosis, thus leading to cancer cell death.

### G13 caused G2/M cell cycle arrest in MDA-MB-231 cells

CBSIs have been shown to induce mitotic arrest and block the cell cycle at the G2/M phase in various human carcinoma cells^34^^,^^41^^,^^42^. Therefore, the effect of **G13** on cell cycle progression of MDA-MB-231 cells was investigated by flow cytometry assay. As depicted in Figure 8(B), cells treated with **G13** at 0.5 μM for 24 h showed an obvious G2/M cell cycle arrest with a ratio of 77.17%, which was lower than that of colchicine group (89.90%). In contrast, the ratio of G2/M phase for the control group was 25.97%. Taken together, this assay revealed **G13** potently induced an obvious G2/M arrest in MDA-MB-231 cells, which was consistent with the effect of CBSIs.

### G13 decreased MMP and increased intracellular ROS level in MDA-MB-231 cells

**Figure 9.**
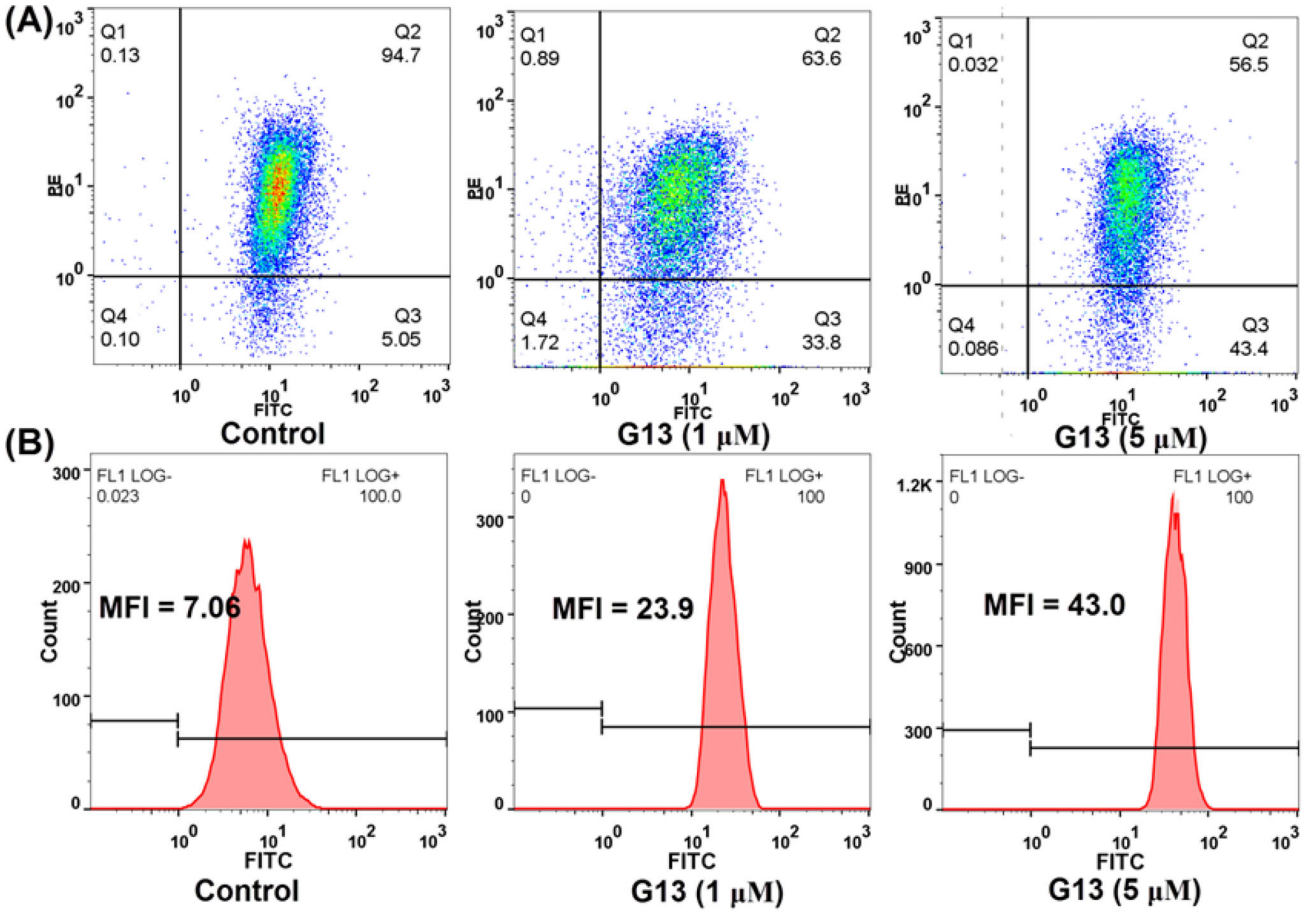
The effect of **G13** on MMP and intracellular ROS level in MDA-MB-231 cells. (A) MDA-MB-231 cells were treated with 0.1% DMSO (control group), or **G13** (1 μM or 5 μM) for 24 h, stained with JC-1 dye and analysed by flow cytometry. (B) ROS level was detected after treatment with 0.1% DMSO (control group), or **G13** (1 μM or 5 μM) for 24 h. The data are representative of two independent experiments.

TEM results indicated **G13** induced the severe damage of mitochondria. The alteration of mitochondrial membrane potential (MMP, ΔΨm) can change the outer and inner membrane permeability of mitochondrial and lead to cytochrome *c* release and caspases activation, thus efficiently inducing cell apoptosis^43^. To explore the effects of **G13** on MMP, JC-1 dye was used to assess the MMP alteration in MDA-MB-231 cells^34^. As depicted in Figure 9(A), cells treated with 0.1% DMSO displayed well-defined integral mitochondrial membranes (monomers: aggregates  =  5.05: 94.7). After treating with **G13** at 1 μM or 5 μM, cells with high MMP were decreased to 63.6% and 56.5%, respectively. Accordingly, cells with low MMP were increased to 33.8% and 43.4%, respectively. This assay revealed **G13** decreased MMP in a concentration dependent manner which effectively induced the apoptosis of cancer cells.

The intracellular reactive oxygen species (ROS) level plays an important role in triggering cell apoptosis. To explore the effect of **G13** on intracellular ROS level, MDA-MB-231 cells were treated with **G13** at 1 μM or 5 μM for 24 h, then added the oxidation-sensitive dye 2′,7′-dichlorodihydrofluorescein diacetate (DCFH-DA) to monitor the change of intracellular ROS level. DCFH-DA can be oxidised by intracellular ROS to afford the fluorescent product 2′,7′-dichlorofluorescein (DCF), which can be detected by flow cytometry to quantify the intracellular ROS level. As depicted in Figure 9(B), cells treated with **G13** at 1 μM or 5 μM showed an obvious increase of intracellular ROS level with the mean fluorescence intensity (MFI) value of 23.9 and 43, respectively. In contrast, MFI value of the control group was 7.06. This assay revealed **G13** efficiently increased the intracellular ROS level, thus triggering the cell apoptosis.

### G13 exhibited good in vivo antitumour efficacy in MDA-MB-231 xenograft model

**Figure 10.**
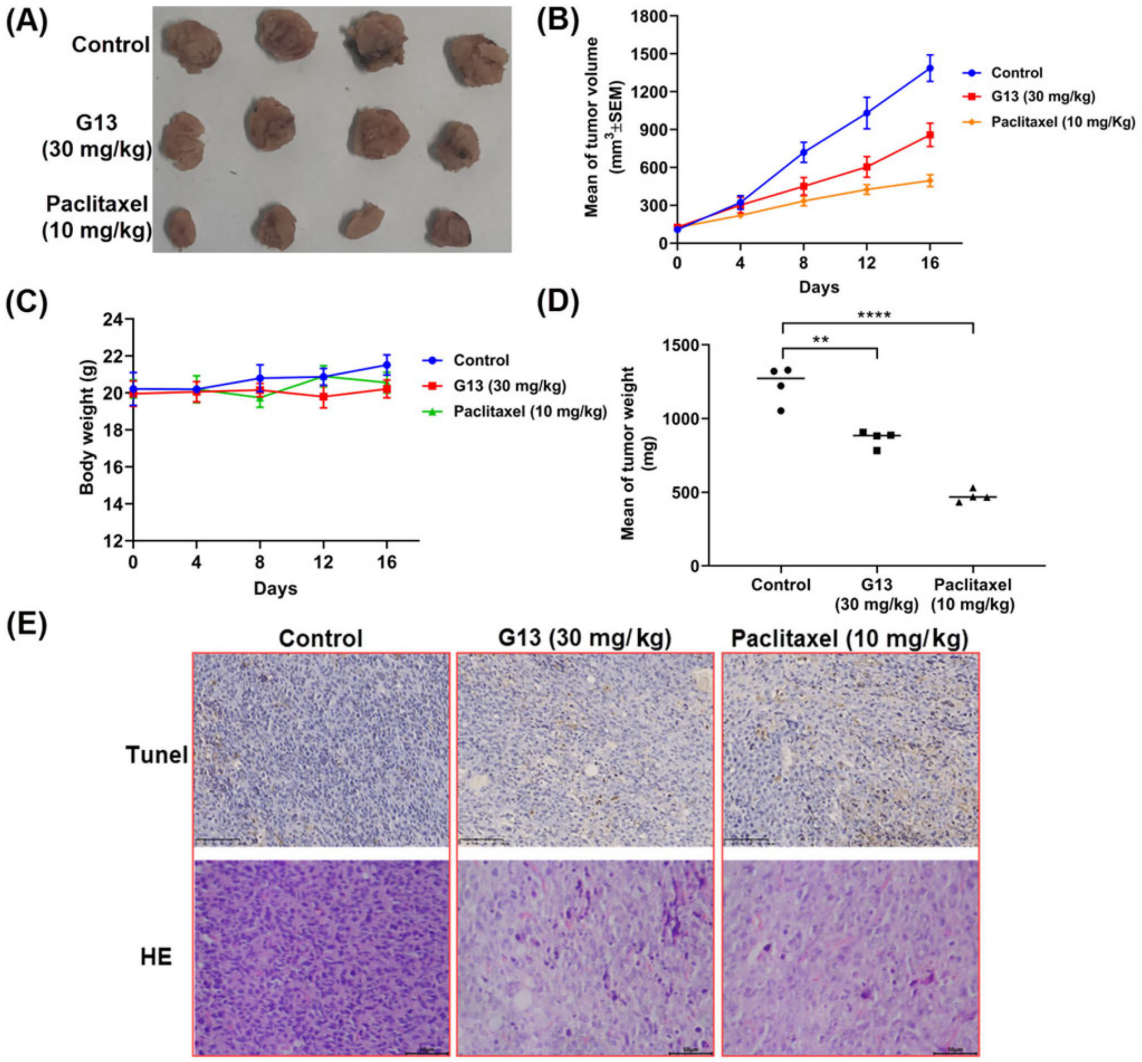
*In vivo* antitumour potency of **G13** in MDA-MB-231 xenograft model. (A) Picture of dissected tumour tissue. (B) Change in tumour size. (C) Change in body weight. (D) Tumour weight. (E) TUNEL and H&E staining pictures of dissected tumour tissue. **p < .01, ****p < .0001, as determined by unpaired *t* test.

**G13** displayed potent *in vitro* antitumour activity and valuable antitumour properties. To evaluate *in vivo* antitumour potency of **G13**, MDA-MB-231 xenograft nude mice model was used in this study. After the tumour size was about 100 mm^3^, the mice were randomly separated into three groups (*n* = 4) and administrated by paclitaxel or **G13** once three days via intraperitoneal (i.p.) injection. Control group was treated by saline. The body weight and tumour size were measured once four days. As depicted in Figure 10, **G13** displayed good *in vivo* antitumour potency at the dose of 30 mg/kg with the tumour growth inhibition (TGI) value of 38.2%. Paclitaxel at the dose of 10 mg/kg displayed potent *in vivo* antitumour potency (TGI  =  64.3%). More importantly, no obvious body weight change was observed in **G13** treated group, indicating **G13** was well tolerated and had a low toxicity (Figure 10(C)). Furthermore, the tumours were dissected and performed TUNEL and H&E staining. As shown in Figure 10(E), images of TUNEL and H&E staining showed more apoptotic cells and necrotic cells in **G13** and paclitaxel treated group as compared with the control group. Taken together, *in vivo* antitumour assay revealed **G13** exhibited good *in vivo* antitumour activity with a low toxicity, and it significantly induced *in vivo* tumour necrosis and apoptosis, thus representing a promising lead structure for further optimisation.

## Conclusion

CBSIs are promising to surmount drug resistance faced by the available microtubule-targeting drugs. For improving antitumour efficacy of our previous reported lead structure, herein, structure based drug design and structural optimisation were performed to afford a series of analogues with diverse substituents and scaffolds. Among them, analogue **G13** displayed potent tubulin polymerisation inhibitory activity (IC_50_  =  13.5 μM) and excellent *in vitro* antiproliferative activity. **G13** potently inhibited the migration and invasion of MDA-MB-231 cells, and exerted potent antiangiogenic activity. More importantly, it exhibited good *in vivo* antitumour efficacy in MDA-MB-231 xenograft models (TGI  =  38.2%; i.p., 30 mg/kg). Despite of the fact that hundreds of diverse molecules have been reported to interact with the colchicine site, compound **G13** displayed a number of preferable advantages, including good drug-like properties with a low molecular weight (M.W.  =  339) and a suitable clogP value (3.45), as well as an easy-to-modify chemical structure, thus representing a good lead structure for further modification. Taken together, our work highlights **G13** is a valuable lead compound for the development of novel tubulin-targeted antitumour agents. Further structural modification and antitumour evaluation are currently underway in our lab.

## Experimental

### Chemistry

^1^H NMR and ^13^C NMR spectra were recorded on Bruker AVANCE400 spectrometer (Bruker Company, Germany), using TMS as an internal standard and CDCl_3_ or DMSO-*d*_6_ as solvents. Chemical shift are given in ppm (*δ*). The mass spectra were recorded on an Esquire 3000 LC-MS mass spectrometer. Silica gel thin-layer chromatography was performed on precoated plates GF-254. All solvents and reagents were analytically pure, and no further purification was needed. All starting materials were commercially available.

#### Chemical synthesis of compounds C1∼J2

##### 2-(Benzo[d][1,3]dioxol-5-yl)-6-methoxyquinoline-4-carboxylic acid (C1)

A solution of 5-methoxyindoline-2,3-dione (150 mg, 0.85 mmol), KOH (143 mg, 2.55 mmol), and 1-(benzo[d][1,3]dioxol-5 -yl)ethan-1-one (140 mg, 0.85 mmol) in EtOH (15 mL) was refluxed at 80 °C for 24 h. Then hydrochloric acid solution was added to adjust pH to 2. Compound **C1** was obtained as yellow solid (186 mg, yield  =  68%) via filtration. ^1^H-NMR (400 MHz, DMSO-*d*_6_): δ 3.92 (s, 3H), 6.14 (s, 2H), 7.09 (d, J = 8.1 Hz, 1H), 7.52 (dd, J  =  2.9 Hz, 9.1 Hz, 1H), 7.79 (dd, J = 2.3 Hz, 8.2 Hz, 1H), 7.83 (s, 1H), 8.09–8.11 (m, 2H), 8.41 (s, 1H). HRMS (ESI positive) m/z calcd for C_18_H_14_NO_5_ (M + 1): 324.0872, found 324.0877.

##### Methyl-2-(benzo[d][1,3]dioxol-5-yl)-6-methoxyquinoline-4-carboxylate (D1)

A solution of **C1** (40 mg, 0.12 mmol) and SOCl_2_ (44 mg, 0.37 mmol) in DCM (5 mL) was refluxed for 6 h. Then the solvent was evaporated and the residue was dissolved in DCM (5 mL). Methanol (50 μL) was added and the mixture was stirred at room temperature for 30 min. After reaction, the solvent was evaporated under reduced pressure and the product **D1** was obtained as white solid (26 mg, yield  =  64%) by column chromatography. ^1^H-NMR (400 MHz, DMSO-*d*_6_): δ 3.99 (s, 3H), 4.08 (s, 3H), 6.07 (s, 2H), 6.97 (d, J = 8.2 Hz, 1H), 7.44 (d, J = 8.2 Hz, 1H), 7.71 (d, J = 8.2 Hz, 1H), 7.76 (s, 1H), 8.22 (d, J = 2.6 Hz, 1H), 8.37 (s, 1H). HRMS (ESI positive) m/z calcd for C_19_H_16_NO_5_ (M + 1): 338.1028, found 338.1036.

##### 2-(Benzo[d][1,3]dioxol-5-yl)-6-methoxyquinoline-4-carboxamide (D2)

A solution of **C1** (40 mg, 0.12 mmol) and SOCl_2_ (44 mg, 0.37 mmol) in DCM (5 mL) was refluxed for 6 h. Then the solvent was evaporated and the residue was dissolved in DCM (5 mL). Ammonia (100 μL) was added and the mixture was stirred at room temperature for 30 min. After reaction, the solvent was evaporated under reduced pressure and the product **D2** was obtained as white solid (23 mg, yield  =  58%) by column chromatography. ^1^H-NMR (400 MHz, DMSO-*d*_6_): δ 3.88 (s, 3H), 6.12 (s, 2H), 7.08 (d, J = 8.1 Hz, 1H), 7.45 (dd, J  =  2.9 Hz, 9.1 Hz, 1H), 7.67 (d, J = 2.7 Hz, 1H), 7.81 (d, J = 1.7 Hz, 1H), 7.83–7.87 (m, 2H), 7.99 (d, J = 9.2 Hz, 1H), 8.07 (s, 1H), 8.30 (s, 1H). HRMS (ESI positive) m/z calcd for C_18_H_15_N_2_O_4_ (M + 1): 323.1032, found 323.1033.

##### 2-(Benzo[d][1,3]dioxol-5-yl)-N-hydroxy-6-methoxyquinoline-4-carboxamide (D3)

A solution of compound **C1** (20 mg, 0.062 mmol) and 1,1′-carbonyldiimidazole (15 mg, 0.093 mmol) in THF (2 mL) was stirred at room temperature for 1 h. Then hydroxylamine hydrochloride (9 mg, 0.124 mmol) was added and the solution was stirred for further 12 h. After reaction, the solvent was evaporated under reduced pressure and the product **D3** was obtained as yellow solid (14 mg, yield  =  67%). ^1^H-NMR (400 MHz, DMSO-*d*_6_): δ 3.88 (s, 3H), 6.13 (s, 2H), 7.09 (d, J = 8.0 Hz, 1H), 7.47 (d, J = 9.2 Hz, 1H), 7.58 (s, 1H), 7.80–7.83 (m, 2H), 7.99–8.01 (m, 2H), 9.47 (s, 1H), 11.40 (s, 1H). HRMS (ESI positive) m/z calcd for C_18_H_15_N_2_O_5_ (M + 1): 339.0981, found 339.0988.

##### 2-(Benzo[d][1,3]dioxol-5-yl)-6-methoxy-N-methylquinoline-4-carboxamide (F1)

Compound **C1** (40 mg, 0.12 mmol), methylamine (3.7 mg, 0.12 mmol), EDCI (23 mg, 0.12 mmol) and DMAP (3 mg) were added in DCM (5 mL). The mixture was stirred at room temperature for 6 h. After reaction, the solvent was evaporated under reduced pressure and the product **F1** was obtained as white solid (30 mg, yield  =  74%) by column chromatography. The synthetic procedure for compounds **F2∼F7** was similar to that of **F1**. ^1^H-NMR (400 MHz, DMSO-*d*_6_): δ 2.90 (d, J = 4.6 Hz, 3H), 3.88 (s, 3H), 6.12 (s, 2H), 7.08 (d, J = 8.0 Hz, 1H), 7.45 (dd, J  =  2.7 Hz, 9.2 Hz, 1H), 7.61 (d, J = 2.6 Hz, 1H), 7.82 (d, J = 9.1 Hz, 1H), 7.84 (s, 1H), 7.99 (d, J = 9.2 Hz, 1H), 8.07 (s, 1H), 8.77 (d, J = 4.6 Hz, 1H). HRMS (ESI positive) m/z calcd for C_19_H_17_N_2_O_4_ (M + 1): 337.1188, found 337.1193.

##### 2-(Benzo[d][1,3]dioxol-5-yl)-N-ethyl-6-methoxyquinoline-4-carboxamide (F2)

Yellow solid (24 mg), yield  =  57%. ^1^H-NMR (400 MHz, DMSO-*d*_6_): δ 1.23 (t, J = 7.2 Hz, 3H), 3.87 (s, 3H), 6.12 (s, 2H), 7.09 (d, J = 8.1 Hz, 1H), 7.46 (dd, J = 2.6 Hz, 9.2 Hz, 1H), 7.58 (d, J = 2.8 Hz, 1H), 7.82–7.85 (m, 2H), 7.98 (d, J = 9.2 Hz, 1H), 8.03 (s, 1H), 8.86 (t, J = 5.6 Hz, 1H). HRMS (ESI positive) m/z calcd for C_20_H_19_N_2_O_4_ (M + 1): 351.1345, found 351.1349.

##### 2-(Benzo[d][1,3]dioxol-5-yl)-N-(cyanomethyl)-6-methoxyquinoline-4-carboxamide (F3)

White solid (32 mg), yield  =  74%. ^1^H-NMR (400 MHz, DMSO-*d*_6_): δ 3.88 (s, 3H), 4.18 (d, J = 2.8 Hz, 2H), 6.13 (s, 2H), 7.09 (d, J = 8 Hz, 1H), 7.47 (dd, J = 2.6 Hz, 9 Hz, 1H), 7.58 (d, J = 2.4 Hz, 1H), 7.84 (d, J = 11.6 Hz, 2H), 7.99 (s, 1H), 8.02 (s, 1H), 9.35 (d, J = 5.6 Hz, 1H). HRMS (ESI positive) m/z calcd for C_20_H_16_N_3_O_4_ (M + H): 362.1141 found 362.1143.

##### 2-(Benzo[d][1,3]dioxol-5-yl)-6-methoxy-N,N-dimethylquinoline-4-carboxamide (F4)

Yellow solid (27 mg), yield  =  64%. ^1^H-NMR (400 MHz, DMSO-*d*_6_): δ 2.82 (s, 3H), 3.13 (s, 3H), 3.86 (s, 3H), 6.12 (s, 2H), 6.93 (br, 1H), 7.06 (d, J = 7.2 Hz, 1H), 7.47 (d, J = 9.2 Hz, 1H), 7.84 (d, J = 9.2 Hz, 2H), 7.99 (s, 2H). ^13^C-NMR (100 MHz, CDCl_3_): 34.88, 38.77, 55.67, 101.39, 102.37, 107.60, 108.51, 115.92, 121.40, 122.81, 123.89, 131.56, 142.07, 144.54, 148.47, 148.83, 153.96, 158.36, 168.96. HRMS (ESI positive) m/z calcd for C_20_H_19_N_2_O_4_ (M + H): 351.1345, found 351.1348.

##### 2-(Benzo[d][1,3]dioxol-5-yl)-N-ethyl-6-methoxy-N-methylquinoline-4-carboxamide (F5)

Pale solid (31 mg), yield  =  70%. ^1^H-NMR (400 MHz, DMSO-*d*_6_): δ 1.01 (t, J = 7.1 Hz), 1.28 (t, J = 7.1 Hz), 2.78 (s), 3.13 (s), 3.87 (s), 5.76 (s, 1H), 6.12 (s, 2H), 6.93 (dd, J = 2.8 Hz, 4.4 Hz, 1H), 7.06 (d, J = 8.0 Hz, 1H), 7.47 (d, J = 9.2 Hz, 1H), 7.84–7.86 (m, 2H), 7.99–8.02 (m, 2H). HRMS (ESI positive) m/z calcd for C_21_H_21_N_2_O_4_ (M + H): 365.1501, found 365.1505.

##### 2-(Benzo[d][1,3]dioxol-5-yl)-N-isopropyl-6-methoxy-N-methylquinoline-4-carboxamide (F6)

Pale solid (31 mg), yield = 67%. ^1^H-NMR (400 MHz, DMSO-*d*_6_): δ 1.08 (dd, J  =  22.2 Hz, 6.5 Hz), 1.23–1.28 (m), 2.63 (s), 3.02 (s), 3.86 (s, 3H), 6.12 (s, 2H), 6.90 (dd, J  =  12.1 Hz, 2.1 Hz, 1H), 7.06 (d, J = 8.0 Hz, 1H), 7.47 (d, J = 9.2 Hz, 1H), 7.85 (br, 2H), 7.98–8.02 (m, 2H). HRMS (ESI positive) m/z calcd for C_22_H_23_N_2_O_4_ (M + 1): 379.1658, found 379.1660.

##### (2-(Benzo[d][1,3]dioxol-5-yl)-6-methoxyquinolin-4-yl)(pyrrolidin-1-yl)methanone (F7)

Pale solid (29 mg), yield  =  63%. ^1^H-NMR (400 MHz, DMSO-*d*_6_): δ 1.82 (t, J = 6.8 Hz, 2H), 1.93 (t, J = 6.8 Hz, 2H), 3.16 (t, J = 6.6 Hz, 2H), 3.65 (t, J = 7.0 Hz, 2H), 3.87 (s, 3H), 6.11 (s, 2H), 7.04 (d, J = 2.6 Hz, 1H), 7.06 (d, J = 8.0 Hz, 1H), 7.47 (dd, J = 9.2 Hz, 2.8 Hz, 1H), 7.84–7.86 (m, 2H), 8.01 (d, J = 9.2 Hz, 1H), 8.04 (s, 1H). HRMS (ESI positive) m/z calcd for C_22_H_21_N_2_O_4_ (M + 1): 377.1501, found 377.1502.

##### (2-(Benzo[d][1,3]dioxol-5-yl)-6-methoxyquinolin-4-yl)methanol (G1)

A solution of compound **C1** (40 mg, 0.12 mmol) and LiAlH_4_ (0.24 mmol) in THF (3 mL) was stirred at 0 °C for 15 min. Then the solution was warmed to room temperature and stirred for further 30 min. After reaction, the solvent was evaporated under reduced pressure and the product **G1** was obtained as white solid (27 mg, yield  =  73%) by column chromatography. The synthetic procedure for compounds **G2**, **G3**, **G5**, **G7∼G20** was similar to that of **G1**. ^1^H-NMR (400 MHz, CDCl_3_): δ 3.88 (s, 3H), 5.12 (s, 2H), 5.98 (s, 2H), 6.87 (d, J = 7.9 Hz, 1H), 7.12 (d, J = 2.4 Hz, 1H), 7.31 (dd, J  =  2.4 Hz, 9.2 Hz, 1H), 7.58 (d, J = 9.3 Hz, 1H), 7.65 (s, 1H), 7.81 (s, 1H), 8.01 (d, J = 9.1 Hz, 1H). HRMS (ESI positive) m/z calcd for C_18_H_16_NO_4_ (M + 1): 310.1079, found 310.1084.

##### (2-(3-Fluoro-4-methoxyphenyl)-6-methoxyquinolin-4-yl)methanol (G2)

White solid (22 mg), yield  =  58%. ^1^H-NMR (400 MHz, DMSO-*d*_6_): δ 3.93 (s, 6H), 5.04 (d, J = 7.6 Hz, 2H), 5.61 (t, J = 5.2 Hz, 1H), 7.32–7.41 (m, 3H), 7.97–8.08 (m, 4H). HRMS (ESI positive) m/z calcd for C_18_H_17_FNO_3_ (M + 1): 314.1192, found 314.1195.

##### (2-(3-(Benzyloxy)phenyl)-6-methylquinolin-4-yl)methanol (G3)

White solid (30 mg), yield  =  70%. ^1^H-NMR (400 MHz, CDCl_3_): δ 2.55 (s, 3H), 5.20 (s, 2H), 5.21 (s, 2H), 7.06 (dd, J = 2.0 Hz, 8.0 Hz, 1H), 7.34–7.44 (m, 4H), 7.50 (d, J = 7.2 Hz, 2H), 7.56 (d, J = 8.8 Hz, 1H), 7.66 (s, 1H), 7.70 (d, J = 7.9 Hz, 1H), 7.85 (s, 1H), 7.95 (s, 1H). ^13^C-NMR (100 MHz, CDCl_3_): 21.92, 62.12, 70.16, 113.73, 115.99, 116.27, 117.97, 120.19, 121.65, 124.85, 127.51, 127.64, 127.98, 128.59, 129.58, 129.79, 130.05, 131.72, 136.44, 137.04, 141.15, 156.10, 159.32. HRMS (ESI positive) m/z calcd for C_24_H_22_NO_2_ (M + 1): 356.1651, found 356.1655.

##### 3-(4-(Hydroxymethyl)-6-methylquinolin-2-yl)phenol (G4)

A solution of compound **G3** (20 mg, 0.056 mmol) and Pd/C (5 mg) in MeOH (3 mL) was stirred under hydrogen atmosphere at room temperature for 12 h. After reaction, the target compound **G4** was obtained as pale solid (12 mg, yield  =  81%) by column chromatography. The synthetic procedure for compound **G6** was similar to that of **G4**. ^1^H-NMR (400 MHz, DMSO-*d*_6_): δ 2.52 (s, 3H), 5.05 (s, 2H), 5.65 (s, 1H), 6.88 (d, J = 8.0 Hz, 1H), 7.33 (t, J = 7.8 Hz, 1H), 7.60 (t, J = 7.7 Hz, 2H), 7.67 (s, 1H), 7.81 (s, 1H), 7.95 (d, J = 8.5 Hz, 1H), 8.03 (s, 1H), 9.63 (s, 1H). HRMS (ESI positive) m/z calcd for C_17_H_16_NO_2_ (M + 1): 266.1181, found 266.1180.

##### (2-(3-(benzyloxy)-4-methoxyphenyl)-6-methylquinolin-4-yl)methanol (G5)

Pale solid (28 mg), yield  =  60%. ^1^H-NMR (400 MHz, DMSO-*d*_6_): δ 2.52 (s, 3H), 3.87 (s, 3H), 5.05 (d, J = 5.6 Hz, 2H), 5.23 (s, 2H), 5.59 (d, J = 5.5 Hz, 1H), 7.16 (d, J = 8.5 Hz, 1H), 7.36 (t, J = 7.3 Hz, 1H), 7.47–7.39 (m, 2H), 7.54 (d, J = 7.5 Hz, 2H), 7.59 (d, J = 8.7 Hz, 1H), 7.79–7.81 (m, 2H), 7.94 (d, J = 8.5 Hz, 1H), 7.98 (s, 1H), 8.07 (s, 1H). HRMS (ESI positive) m/z calcd for C_25_H_24_NO_3_ (M + 1): 386.1756, found 386.1745.

##### 5-(4-(Hydroxymethyl)-6-methylquinolin-2-yl)-2-methoxyphenol (G6)

Pale solid (11 mg), yield  =  67%. ^1^H-NMR (400 MHz, DMSO-*d*_6_): δ 2.50 (s, 3H), 3.85 (s, 3H), 5.04 (d, J = 5.6 Hz, 2H), 5.59 (t, J = 5.6 Hz, 1H), 7.07 (d, J = 8.5 Hz, 1H), 7.57 (d, J = 8.6 Hz, 1H), 7.64 (d, J = 8.3 Hz, 1H), 7.77 (d, J = 7.6 Hz, 2H), 7.92 (d, J = 8.6 Hz, 1H), 8.01 (s, 1H), 9.24 (s, 1H). HRMS (ESI positive) m/z calcd for C_18_H_18_NO_3_ (M + 1): 296.1287, found 296.1273.

##### (6-Methyl-2-phenylquinolin-4-yl)methanol (G7)

Pale solid (22 mg), yield  =  73%. ^1^H-NMR (400 MHz, DMSO-*d*_6_): δ 2.53 (s, 3H), 5.06 (s, 2H), 5.62 (s, 1H), 7.47–7.62 (m, 4H), 7.83 (s, 1H), 7.99 (d, J = 8.5 Hz, 1H), 8.11 (s, 1H), 8.23 (d, J = 7.6 Hz, 2H). ^13^C-NMR (100 MHz, CDCl_3_): 21.90, 62.10, 116.19, 121.65, 124.75, 127.46, 128.77, 129.21, 130.02, 131.70, 136.36, 139.64, 145.72, 146.64, 156.40. HRMS (ESI positive) m/z calcd for C_17_H_16_NO (M + 1): 250.1232, found 250.1229.

##### (2-(P-tolyl)quinolin-4-yl)methanol (G8)

Pale solid (23 mg), yield  =  76%. 1H-NMR (400 MHz, CDCl_3_): δ 2.43 (s, 3H), 5.23 (s, 2H), 7.30 (d, J = 7.9 Hz, 2H), 7.57–7.49 (m, 1H), 7.77–7.69 (m, 1H), 7.90 (d, J = 8.4 Hz, 1H), 7.98 (s, 1H), 8.06 (d, J = 7.9 Hz, 2H), 8.25 (d, J = 8.5 Hz, 1H). ^13^C-NMR (100 MHz, CDCl_3_): 21.31, 61.92, 115.98, 122.61, 124.68, 126.15, 127.42, 129.41, 129.50, 130.11, 136.67, 139.50, 146.53, 147.99, 157.26. HRMS (ESI positive) m/z calcd for C_17_H_16_NO (M + 1): 250.1232, found 250.1227.

##### (2-(4-Chlorophenyl)quinolin-4-yl)methanol (G9)

Brown solid (18 mg), yield  =  56%. ^1^H-NMR (400 MHz, DMSO-*d*_6_): δ 5.09 (d, J = 5.5 Hz, 2H), 5.67 (t, J = 5.5 Hz, 1H), 7.64 (br, 3H), 7.79 (t, J = 7.7 Hz, 1H), 8.06–8.11 (m, 2H), 8.17 (s, 1H), 8.29 (d, J = 8.4 Hz, 2H). ^13^C-NMR (100 MHz, CDCl_3_): 60.83, 114.59, 121.56, 123.73, 125.59, 127.78, 127.96, 128.65, 129.29, 134.62, 136.91, 145.75, 146.99, 154.96. HRMS (ESI positive) m/z calcd for C_16_H_13_ClNO (M + 1): 270.0686, found 270.0691.

##### (2-(3,4,5-Trimethoxyphenyl)quinolin-4-yl)methanol (G10)

White solid (25 mg), yield  =  64%. ^1^H-NMR (400 MHz, DMSO-*d*_6_): δ 3.75 (s, 3H), 3.93 (s, 6H), 5.09 (d, J = 4.7 Hz, 2H), 5.64 (t, J = 4.7 Hz, 1H), 7.55 (s, 2H), 7.56–7.62 (m, 1H), 7.77 (t, J = 8.0 Hz, 1H), 8.09 (m, 2H), 8.16 (s, 1H). HRMS (ESI positive) m/z calcd for C_19_H_20_NO_4_ (M + 1): 326.1392, found 326.1393.

##### (5-Fluoro-2-(3,4,5-trimethoxyphenyl)quinolin-4-yl)methanol (G11)

Pale solid (23 mg), yield  =  59%. ^1^H-NMR (400 MHz, DMSO-*d*_6_): δ 3.76 (s, 5H), 3.93 (s, 6H), 5.15 (d, J = 4.9 Hz, 2H), 5.70 (t, J = 4.9 Hz, 1H), 7.38 (dd, J  =  12.1 Hz, 7.5 Hz, 1H), 7.52 (s, 2H), 7.77 − 7.70 (m, 1H), 7.94 (d, J = 8.0 Hz, 1H), 8.27 (s, 1H). HRMS (ESI positive) m/z calcd for C_19_H_19_FNO_4_ (M + 1): 344.1298, found 344.1290.

##### (6-Fluoro-2-(3,4,5-trimethoxyphenyl)quinolin-4-yl)methanol (G12)

Brown solid (21 mg), yield  =  51%. ^1^H-NMR (400 MHz, DMSO-*d*_6_): δ 3.75 (s, 3H), 3.93 (s, 6H), 5.03 (d, J = 4.8 Hz, 2H), 5.67 (t, J = 4.7 Hz, 1H), 7.53 (s, 2H), 7.67 (dt, J = 2.8 Hz, 9.0 Hz, 1H), 7.85 (dd, J  =  10.3 Hz, 2.8 Hz, 1H), 8.20–8.12 (m, 2H). HRMS (ESI positive) m/z calcd for C_19_H_19_FNO_4_ (M + 1): 344.1298, found 344.1290.

##### (6-Methyl-2-(3,4,5-trimethoxyphenyl)quinolin-4-yl)methanol (G13)

White solid (27 mg), yield  =  66%. ^1^H-NMR (400 MHz, DMSO-*d*_6_): δ 3.75 (s, 3H), 3.93 (s, 6H), 5.06 (d, J = 5.5 Hz, 2H), 5.67 (t, J = 5.5 Hz, 1H), 7.52 (s, 2H), 7.60 (d, *J* = 8.7 Hz, 1H), 7.85 (s, 1H), 7.98 (d, *J* = 8.4 Hz, 1H), 8.11 (s, 1H). HRMS (ESI positive) m/z calcd for C_20_H_22_NO_4_ (M + 1): 340.1549, found 340.1541.

##### (6-Methoxy-2-(3,4,5-trimethoxyphenyl)quinolin-4-yl)methanol (G14)

White solid (25 mg), yield  =  59%. ^1^H-NMR (400 MHz, DMSO-*d*_6_): δ 3.74 (s, 3H), 3.92 (s, 9H), 5.05 (d, J = 5.5 Hz, 2H), 5.62 (t, J = 5.5 Hz, 1H), 7.34 (d, J = 2.8 Hz, 1H), 7.41 (dd, J  =  9.2 Hz, 2.7 Hz, 1H), 7.50 (s, 2H), 8.00 (d, J = 9.2 Hz, 1H), 8.11 (s, 1H). ^13^C-NMR (100 MHz, DMSO-*d*_6_): 55.52, 56.02, 60.11, 60.29, 78.61, 102.04, 104.21, 115.78, 121.46, 125.68, 131.10, 134.64, 138.72, 143.18, 147.04, 153.06, 153.17, 157.04. HRMS (ESI positive) m/z calcd for C_20_H_22_NO_5_ (M + 1): 356.1498, found 356.1493.

##### (6-(Trifluoromethoxy)-2-(3,4,5-trimethoxyphenyl)quinolin-4-yl)methanol (G15)

Yellow solid (20 mg), yield  =  40%. ^1^H-NMR (400 MHz, DMSO-*d*_6_): δ 3.75 (s, 3H), 3.93 (s, 6H), 5.06 (d, J = 5.3 Hz, 2H), 5.71 (t, J = 5.3 Hz, 1H), 7.57 (s, 2H), 7.76 (d, J = 9.2 Hz, 1H), 8.08 (s, 1H), 8.20–8.25 (m, 2H). HRMS (ESI positive) m/z calcd for C_20_H_19_F_3_NO_5_ (M + 1): 410.1215, found 410.1202.

##### (7-Methoxy-2-(3,4,5-trimethoxyphenyl)quinolin-4-yl)methanol (G16)

White solid (22 mg), yield  =  51%. ^1^H-NMR (400 MHz, DMSO-*d*_6_): δ 3.75 (s, 3H), 3.93 (s, 6H), 3.95 (s, 3H), 5.03 (d, J = 5.5 Hz, 2H), 5.57 (t, J = 5.5 Hz, 1H), 7.22 (dd, J  =  9.0 Hz, 2.0 Hz, 1H), 7.45 (d, J = 2.0 Hz, 1H), 7.54 (s, 2H), 7.99 (d, J = 8.4 Hz, 1H), 8.00 (s, 1H). HRMS (ESI positive) m/z calcd for C_20_H_22_NO_5_ (M + 1): 356.1498, found 356.1492.

##### (8-Methoxy-2-(3,4,5-trimethoxyphenyl)quinolin-4-yl)methanol (G17)

Yellow solid (24 mg), yield  =  56%. ^1^H-NMR (400 MHz, CDCl_3_): δ 3.90 (s, 3H), 4.00 (s, 6H), 4.12 (s, 3H), 5.25 (s, 2H), 7.12 (d, J = 7.4 Hz, 1H), 7.38 (s, 2H), 7.44 − 7.54 (m, 2H), 8.10 (s, 1H). HRMS (ESI positive) m/z calcd for C_20_H_22_NO_5_ (M + 1): 356.1498, found 356.1490.

##### (6-Methoxy-2-(1H-pyrrol-2-yl)quinolin-4-yl)methanol (G18)

Brown solid (25 mg), Yield  =  81%. ^1^H-NMR (400 MHz, DMSO-*d*_6_): δ 3.90 (s, 3H), 4.98 (s, 2H), 5.58 (br, 1H), 6.19 (s, 1H), 6.84 (s, 1H), 6.92 (s, 1H), 7.25 (s, 1H), 7.35 (d, J = 9.1 Hz, 1H), 7.85–7.89 (m, 2H), 11.51 (s, 1H). HRMS (ESI positive) m/z calcd for C_15_H_15_N_2_O_2_ (M + 1): 255.1134, found 255.1133.

##### (2-(Furan-2-yl)-6-methylquinolin-4-yl)methanol (G19)

Yellow solid (24 mg), yield  =  83%. ^1^H-NMR (400 MHz, DMSO-*d*_6_): δ 5.03 (d, J = 5.3 Hz, 2H), 5.63 (t, J = 5.5 Hz, 1H), 6.72 (s, 1H), 7.28 (d, J = 3.3 Hz, 1H), 7.59 (d, J = 8.7 Hz, 1H), 7.77 (s, 1H), 7.89–7.92 (m, 2H), 7.97 (s, 1H). HRMS (ESI positive) m/z calcd for C_15_H_14_NO_2_ (M + 1): 240.1025, found 240.1032.

##### (2-(Benzofuran-2-yl)-6-methylquinolin-4-yl)methanol (G20)

Yellow solid (18 mg), yield  =  51%. ^1^H-NMR (400 MHz, DMSO-*d*_6_): δ 2.54 (s, 3H), 5.09 (s, 2H), 7.33 (t, J = 7.6 Hz, 1H), 7.43 (t, J = 7.8 Hz, 1H), 7.65 (d, J = 8.7 Hz, 1H), 7.72–7.81 (m, 3H), 7.84 (s, 1H), 8.01 (d, J = 8.5 Hz, 1H), 8.19 (s, 1H). HRMS (ESI positive) m/z calcd for C_19_H_16_NO_2_ (M + 1): 290.1181, found 290.1180.

##### 4-(Chloromethyl)-6-methyl-2–(3,4,5-trimethoxyphenyl)quinoline (G21)

To a solution of **G13** (20 mg, 0.059 mmol) in DCM (5 mL), SOCl_2_ (172 μL) was added and the mixture stirred at 0 °C for 3 h. After reaction, the solvent was evaporated under reduced pressure and the product **G21** was obtained as yellow solid (11 mg, yield  =  52%) by column chromatography. ^1^H-NMR (400 MHz, CDCl_3_): δ 2.66 (s, 3H), 3.96 (s, 3H), 4.11 (s, 6H), 5.16 (s, 2H), 7.60 (s, 2H), 7.83 (d, J = 8.1 Hz, 1H), 7.89 (s, 1H), 8.15 (s, 1H), 9.56 (d, J = 8.4 Hz, 1H). HRMS (ESI positive) m/z calcd for C_20_H_21_ClNO_3_ (M + 1): 358.1210, found 358.1201.

##### 4-(Bromomethyl)-6-methyl-2-(3,4,5-trimethoxyphenyl)quinoline (G22)

**G13** (40 mg, 0.12 mmol), NBS (43 mg, 0.24 mmol) and PPh_3_ (62 mg, 0.24 mmol) were added in THF (3 mL) at 0 °C. Then the solution was warmed to room temperature and stirred for 24 h. After reaction, the solvent was evaporated under reduced pressure and the product **G22** was obtained as yellow solid (26 mg, yield  =  54%) by column chromatography. ^1^H-NMR (400 MHz, CDCl_3_): δ 2.66 (s, 3H), 3.96 (s, 3H), 4.12 (s, 7H), 4.98 (s, 2H), 7.55 (s, 2H), 7.82–7.88 (m, 2H), 8.14 (s, 1H), 9.62 (d, J = 6.4 Hz, 1H). ^13^C-NMR (100 MHz, CDCl_3_): 21.95, 51.88, 56.39, 60.96, 104.92, 118.11, 121.71, 124.95, 130.24, 132.11, 135.04, 136.91, 139.67, 140.29, 147.01, 153.64, 155.96. HRMS (ESI positive) m/z calcd for C_20_H_21_BrNO_3_ (M + 1): 402.0705, found 402.0706.

##### 4-(Azidomethyl)-6-methyl-2-(3,4,5-trimethoxyphenyl)quinoline (G23)

A solution of **G22** (20 mg, 0.05 mmol), NaN_3_ (6.5 mg, 0.1 mmol) and H_2_O (30 μL) in DMF (1 mL) was stirred at room temperature for 24 h. After reaction, the solvent was evaporated under reduced pressure and the product **G23** was obtained as pale solid (11 mg, yield  =  60%) by column chromatography. ^1^H-NMR (400 MHz, CDCl_3_): δ 2.59 (s, 3H), 3.92 (s, 3H), 4.02 (s, 6H), 4.89 (s, 2H), 7.39 (s, 2H), 7.60 (d, J = 8.5 Hz, 1H), 7.71 (s, 1H), 7.79 (s, 1H), 8.11 (d, J = 8.6 Hz, 1H). HRMS (ESI positive) m/z calcd for C_20_H_21_N_4_O_3_ (M + 1): 365.1614, found 365.1610.

##### (6-Methyl-2-(3,4,5-trimethoxyphenyl)quinolin-4-yl)methanamine (G24)

A solution of **G23** (30 mg, 0.08 mmol), PPh_3_ (44 mg, 0.17 mmol) and H_2_O (200 μL) in THF (3 mL) was stirred at room temperature for 24 h. After reaction, the solvent was evaporated under reduced pressure and the product **G24** was obtained as white solid (12 mg, yield  =  44%) by column chromatography. ^1^H-NMR (400 MHz, DMSO-*d*_6_): δ 2.53 (s, 3H), 3.75 (s, 3H), 3.93 (s, 6H), 4.26 (s, 2H), 7.56–7.60 (m, 3H), 7.90 (s, 1H), 7.97 (d, J = 8.6 Hz, 1H), 8.14 (s, 1H). HRMS (ESI positive) m/z calcd for C_20_H_23_N_2_O_3_ (M + 1): 339.1709, found 339.1699.

##### 8-Methoxy-11H-indeno[1,2-b]quinoline-10-carboxylic acid (I1)

A solution of 5-methoxyindoline-2,3-dione (200 mg, 1.14 mmol), 2,3-dihydro-1*H*-inden-1-one (150 mg, 1.14 mmol) and KOH (191 mg, 3.41 mmol) in EtOH (15 mL) was refluxed at 80 °C for 24 h. Then hydrochloric acid solution was added to adjust pH to 2. Compound **I1** was obtained via filtration as brown solid (189 mg, yield  =  57%). The synthetic procedure for **I2** was similar to that of **I1**. ^1^H-NMR (400 MHz, DMSO-*d*_6_): δ 3.90 (s, 3H), 4.22 (s, 2H), 7.44 (d, J = 9.2 Hz, 1H), 7.52 (br, 2H), 7.70 (d, J = 6.4 Hz, 1H), 7.90 (s, 1H), 8.13–8.03 (m, 2H). HRMS (ESI positive) m/z calcd for C_18_H_14_NO_3_ (M + 1): 292.0974, found 292.0966.

##### 2,3-Dimethoxy-8-methyl-11H-indeno[1,2-b]quinoline-10-carboxylic acid (I2)

Brown solid (165 mg), yield  =  43%. ^1^H-NMR (400 MHz, DMSO-*d*_6_): δ 3.87 (s, 3H), 3.88 (s, 3H), 3.91 (s, 3H), 4.07 (s, 2H), 7.30 (s, 1H), 7.42 (d, J = 9.0 Hz, 1H), 7.55 (s, 1H), 7.86 (s, 1H), 7.99 (d, J = 9.0 Hz, 1H). HRMS (ESI positive) m/z calcd for C_20_H_18_NO_4_ (M + 1): 336.1236, found 336.1229.

##### (8-Methoxy-11H-indeno[1,2-b]quinolin-10-yl)methanol (J1)

To a solution of **I1** (37 mg, 0.127 mmol) and NaBH_4_ (14.2 mg, 0.375 mmol) in THF (3 mL), BF_3_·OEt_2_ (60 μL) was added and the mixture was stirred at 0 °C for 15 min. Then the solution was warmed to room temperature and stirred for additional 16 h. After reaction, NaOH (20 mg, 0.5 mmol) in H_2_O (2 mL) was added and the mixture was stirred for 3 h. The solvent was evaporated under reduced pressure and product **J1** was obtained as yellow solid (11 mg, yield  =  31%) by column chromatography. The synthetic procedure for **J2** was similar to that of **J1**. ^1^H-NMR (400 MHz, DMSO-*d*_6_): δ 3.94 (s, 3H), 4.19 (s, 2H), 5.07 (s, 2H), 5.46 (br, 1H), 7.40 (dd, J  =  2.8 Hz, 9.0 Hz, 1H), 7.53–7.49 (m, 2H), 7.59 (d, J = 2.8 Hz, 1H), 7.71–7.68 (m, 1H), 8.02 (d, J = 9.1 Hz, 1H), 8.06–8.10 (m, 1H). ^13^C-NMR (100 MHz, DMSO-*d*_6_): 32.88, 55.44, 58.18, 103.49, 120.30, 120.86, 125.71, 126.65, 127.29, 129.54, 130.51, 133.12, 139.90, 140.14, 143.69, 144.69, 156.73, 158.43. HRMS (ESI positive) m/z calcd for C_18_H_16_NO_2_ (M + 1): 278.1181, found 278.1178.

##### (2,3-Dimethoxy-8-methyl-11H-indeno[1,2-b]quinolin-10-yl)methanol (J2)

Pale solid (12 mg), yield  =  29%. ^1^H-NMR (400 MHz, DMSO-*d*_6_): δ 2.53 (s, 3H), 3.88 (s, 3H), 3.91 (s, 3H), 4.06 (s, 3H), 5.03 (s, 2H), 7.31 (s, 1H), 7.51–7.58 (m, 2H), 7.94 (d, J = 8.5 Hz, 1H), 8.01 (s, 1H). HRMS (ESI positive) m/z calcd for C_20_H_20_NO_3_ (M + 1): 322.1443, found 322.1438.

### Biological evaluation

#### In vitro antitumour assay

*In vitro* antitumour assay was performed using CCK-8 method. A549, HCT116 and MDA-MB-231 cells were used in this assay. Cells were cultured in the 96-well plates with a density of 8 × 10^3^ cells per well, and incubated in 5% CO_2_ incubator at 37 °C for 24 h. Then fresh medium containing compounds was added, and the cells were cultured for additional 72 h. After the treatment, 10 μL of CCK-8 was added to the well, and the plate was kept at 37 °C for 1 h. The absorbance (OD) at 450 nm was determined using a SYNERGY|LX multi-mode reader (BioTeK). IC_50_ values were calculated using the logit method.

#### Wound healing assay

MDA-MB-231 cells were cultured in six-well plates at a density of 5 × 10^5^ cells per well, and incubated in 5% CO_2_ incubator at 37 °C for 24 h. A wound was made using a sterile 200 μL pipet tip and washed three times with PBS to remove cell debris. The fresh medium containing compounds was added, and the wound area was captured by the inverted microscope at 0 h and 24 h, and measured by Image J software.

#### Trans/ invasion assay

The matrigel (Corning) was mixed with serum-free medium at a ratio of 1: 8. Then the mixture (10 μL) was added on the upper surface of the chamber membrane. The plate was kept in 5% CO_2_ incubator at 37 °C for 2 h. MDA-MB-231 cells (3 × 10^4^) suspended in RPMI-1640 medium containing 1% foetal bovine serum (FBS) and compounds were seeded in the upper chamber. To the lower chamber of the 24-well plate, 800 μL of medium containing 20% FBS was added. After the incubation for 24 h, the cells were fixed with 4% paraformaldehyde, and stained with 0.1% crystal violet. The cells on the upper surface of the membrane was removed using a cotton swab. The invade cells were captured by the inverted microscope, and counted by Image J software.

#### Endothelial tube formation assay

The matrigel (Corning) was mixed with serum-free medium at the ratio of 1: 1. Then the mixture (10 μL) was added to the 24-well plate, and the plate was kept in 5% CO_2_ incubator at 37 °C for 1 h. Then HUVECs (5 × 10^4^) suspended in 200 μL ECM medium containing compounds was added to the plate, and cultured at 37 °C for 6 h. The capillary networks were captured by the inverted microscope.

#### Tubulin polymerisation inhibition assay

This assay was performed according to our previous reports^34^. Tubulin protein isolated from pig brain was dissolved in PEM buffer containing 1 mM GTP, 5% glycerol and compounds. Colchicine was used as the positive control. The absorbance of the mixture at 340 nm was recorded at 37 °C using a SPECTRA MAX 190 spectrophotometer. The plateau absorbance values were used for the calculation. This assay was performed twice, and IC_50_ values were calculated accordingly.

#### Cell cycle assay

MDA-MB-231 cells were cultured in the six-well plate with a density of 2 × 10^5^ cells per well, and incubated in 5% CO_2_ incubator at 37 °C for 24 h. Then fresh medium containing compounds was added, and the cells were cultured for additional 24 h. Cells were treated using PI/RNase staining kit (Elabscience) following the manufacturer’s protocol and subjected to flow-cytometric analysis using Beckman Coulter EPICS XL/XL-MCL instrument.

#### Cell apoptosis assay

MDA-MB-231 cells were cultured in the six-well plate with a density of 2 × 10^5^ cells per well, and incubated in 5% CO_2_ incubator at 37 °C for 24 h. Then fresh medium containing compounds was added, and the cells were cultured for additional 24 h. Cells were treated with annexin V-FITC/PI apoptosis detection kit (Elabscience) following the manufacturer’s instruction and subjected to flow-cytometric analysis using Beckman Coulter EPICS XL/XL-MCL instrument.

#### Cellular reactive oxygen species detection assay

MDA-MB-231 cells were cultured in the six-well plate with a density of 2 × 10^5^ cells per well, and incubated in 5% CO_2_ incubator at 37 °C for 24 h. Then fresh medium containing compounds was added, and the cells were cultured for additional 24 h. Cells were treated with ROS detection kit (Elabscience, E-BC-K138-F) following the manufacturer’s protocol, and subjected to flow-cytometric analysis using Beckman Coulter EPICS XL/XL-MCL instrument.

#### Mitochondrial membrane potential assay

MDA-MB-231 cells were cultured in the six-well plate with a density of 2 × 10^5^ cells per well, and incubated in 5% CO_2_ incubator at 37 °C for 24 h. Then the medium was replaced by fresh medium containing compounds, and the cells were cultured for additional 24 h. Mitochondrial membrane depolarisation was determined using JC-1 detection kit (Solarbio, M8650) following the manufacturer’s protocol. The cells were subjected to flow-cytometric analysis using Beckman Coulter EPICS XL/XL-MCL instrument.

#### Transmission electron microscopy assay

MDA-MB-231 cells were cultured in the six-well plate at a density of 2 × 10^5^ cells per well, and incubated in 5% CO_2_ incubator at 37 °C for 24 h. Then the medium was replaced by fresh medium containing compounds, and the cells were cultured for additional 48 h. The cells were digested with trypsin, and washed three times by PBS. The cells were fixed by 2.5% glutaraldehyde (500 μL), and submitted for TEM observation (JEM-1400, Japan Tokyo).

#### Immunofluorescent assay

This assay was performed according to our previous report^34^. Briefly, 2 × 10^5^ MDA-MB-231 cells were cultured on glass coverslips in the six-well plate at 37 °C for 24 h. Then compound **G13** or 0.1% DMSO were added, and the cells were incubated for additional 24 h. Colchicine was used as the positive control. Anti-α-tubulin antibody (CST, #2125) and Alexa Fluor 594 donkey anti-rabbit IgG (Yeasen, 34212ES60) were used to visualise the microtubules. The nucleus was visualising using DAPI with prolong gold antifade reagent (CST, #8961). The photographs of cells were captured using the Zeiss 810 Confocal microscope.

#### In vivo antitumour assay

MDA-MB-231 xenograft nude mice model was used to evaluate *in vivo* antitumour potency of **G13**. About 3 × 10^6^ MDA-MB-231 cells were subcutaneously inoculated into the right flank of nude mice. About seven days after the implantation, the tumour grew larger than 100 mm^3^. Then the mice were randomly separated into three groups (*n* = 4), and administrated with **G13** (30 mg/kg) dissolved in 5% DMF/1% Tween-80/94% saline or paclitaxol (10 mg/kg) dissolved in EtOH/Cremophor EL/saline once three days via i.p. injection. The mice in the control group were administrated with 0.9% saline via i.p. injection. The tumour size and body weight of nude mice were recorded once four days. About 16 days after the treatment, the mice were sacrificed and the tumours were excised, weighted and performed H&E and TUNEL staining. The tumour volume was calculated using the following formula: V = a × b^2^/2, a and b represent the length and the width of tumour, respectively. This assay was conducted under the supervision of the Ethics Committee of the Air Force Medical University.

### Molecular docking

Molecular docking was performed according to our previous report^34^. The glide docking program with *SP* precision mode in Schrodinger software was used for the molecular simulation. Briefly, the crystal structure of colchicine in complex with tubulin (PDB code: 4O2B) was downloaded from RCSB. The binding pocket was defined as all the residues within 12 Å around the centroid of colchicine in tubulin. The other parameters were used without any change.

## Supplementary Material

Supplemental Material
