# Supplementary material for: Discovery of potent tubulin inhibitors targeting the colchicine binding site via structure-based lead optimization and antitumor evaluation
Source: J Enzyme Inhib Med Chem. 2023 Jan 11;38(1):2155815. doi: 10.1080/14756366.2022.2155815 (PMC9848350; doi:10.1080/14756366.2022.2155815)
Supplement: Supplemental Material [file IENZ_A_2155815_SM2793.pdf]

Discovery of Potent Tubulin Inhibitors Targeting the Colchicine Binding Site via Structure-based Lead Optimization and Antitumor Evaluation

Wei Liu[a, 1], Youyou He[a, b, 1], Zhongjie Guo[b, 1], Miaomiao Wang[a], Xiaodong Han[b], Hairui Jia[a], Jin He[b], Shanshan Miao[b], Shengzheng Wang[b, *]

[a] *Faculty of Pharmacy, School of Food and Biological Engineering, Shaanxi University of Science and Technology, Xi'an, Shaanxi Province, China*

[b] *Department of Medicinal Chemistry and Pharmaceutical Analysis, School of Pharmacy, Fourth Military Medical University, Xi'an, Shaanxi Province, China*

Content:

^{1}H NMR, ^{13}C NMR and HRMS spectra

1. Spectra of compound **C1** Page 4.

2. Spectra of compound **D1** Page 5.

3. Spectra of compound **D2** Page 6.

4. Spectra of compound **D3** Page 7.

5. Spectra of compound **F1** Page 8.

6. Spectra of compound **F2** Page 9.

7. Spectra of compound **F3** Page 10.

8. Spectra of compound **F4** Page 11.

9. Spectra of compound **F5** Page 13.

10. Spectra of compound **F6** Page 14.

11. Spectra of compound **F7** Page 15.

12. Spectra of compound **G1** Page 16.

13. Spectra of compound **G2** Page 17.

14. Spectra of compound **G3** Page 18.

15. Spectra of compound **G4** Page 20.

16. Spectra of compound **G5** Page 21.

17. Spectra of compound **G6** Page 22.

18. Spectra of compound **G7** Page 23.

19. Spectra of compound **G8** Page 25.

20. Spectra of compound **G9** Page 27.

21. Spectra of compound **G10** Page 29.

22. Spectra of compound **G11** Page 30.

23. Spectra of compound **G12** Page 31.

24. Spectra of compound **G13** Page 32.

25. Spectra of compound **G14** Page 34.

26. Spectra of compound **G15** Page 36.

27. Spectra of compound **G16** Page 37.

28. Spectra of compound **G17** Page 38.

29. Spectra of compound **G18** Page 39.

30. Spectra of compound **G19** Page 40.

31. Spectra of compound **G20** Page 41.

32. Spectra of compound **G21** Page 42.

33. Spectra of compound **G22** Page 43.

34. Spectra of compound **G23** Page 45.

35. Spectra of compound **G24** Page 46.

36. Spectra of compound **I1** Page 47.

37. Spectra of compound **I2** Page 48.

38. Spectra of compound **J1** Page 49.

39. Spectra of compound **J2** Page 50.

Compound **C1**

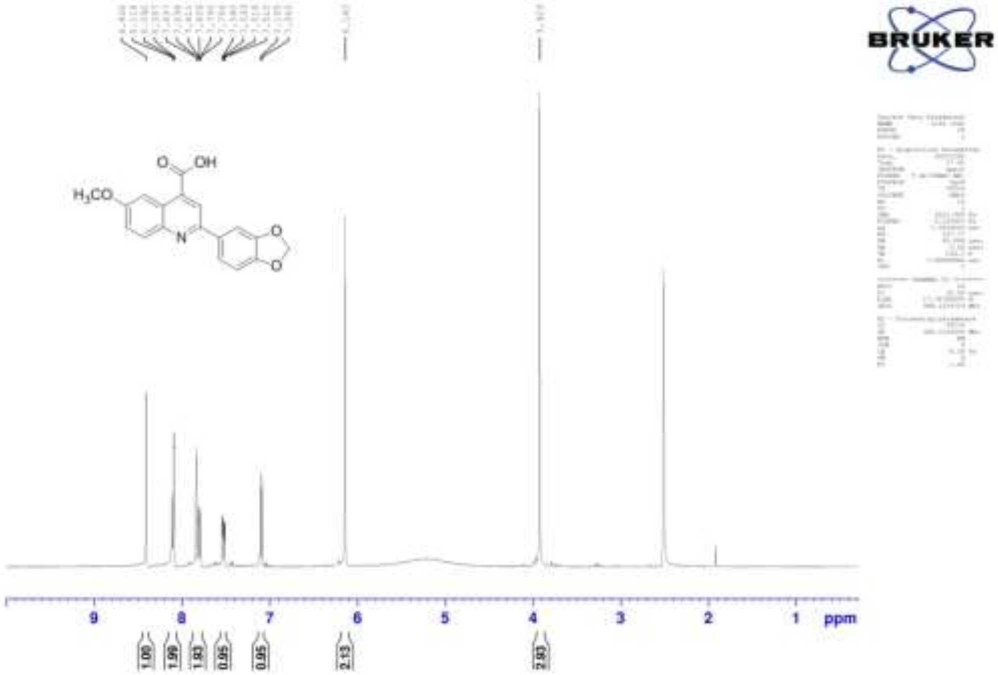

BRUKER
O OH
H_3CO
N
O
9 8 7 6 5 4 3 2 1 ppm
1.00 1.99 1.93 0.95 0.95 2.13 2.93

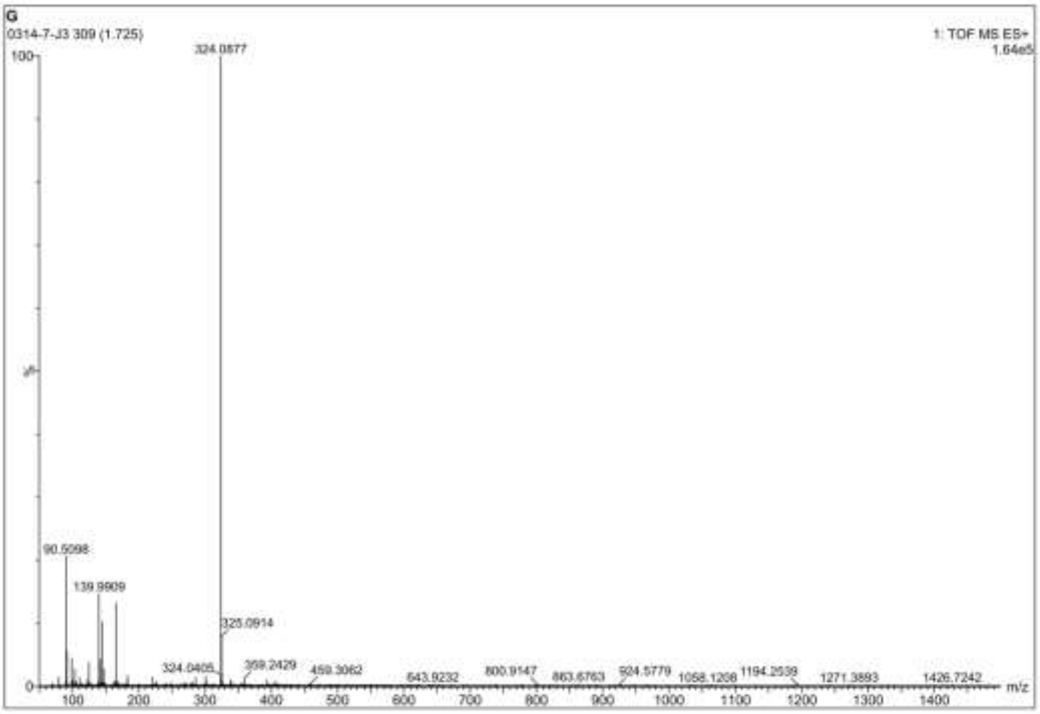

G
0314-7-J3 309 (1.725)
1: TOF MS ES+
1.64e5
324.0877
90.5098
139.9909
325.0914
324.0405
359.2429
459.3062
643.9232
800.9147
863.6763
924.5779
1058.1208
1194.2539
1271.3893
1426.7242
m/z
%
100
0

Compound **D1**

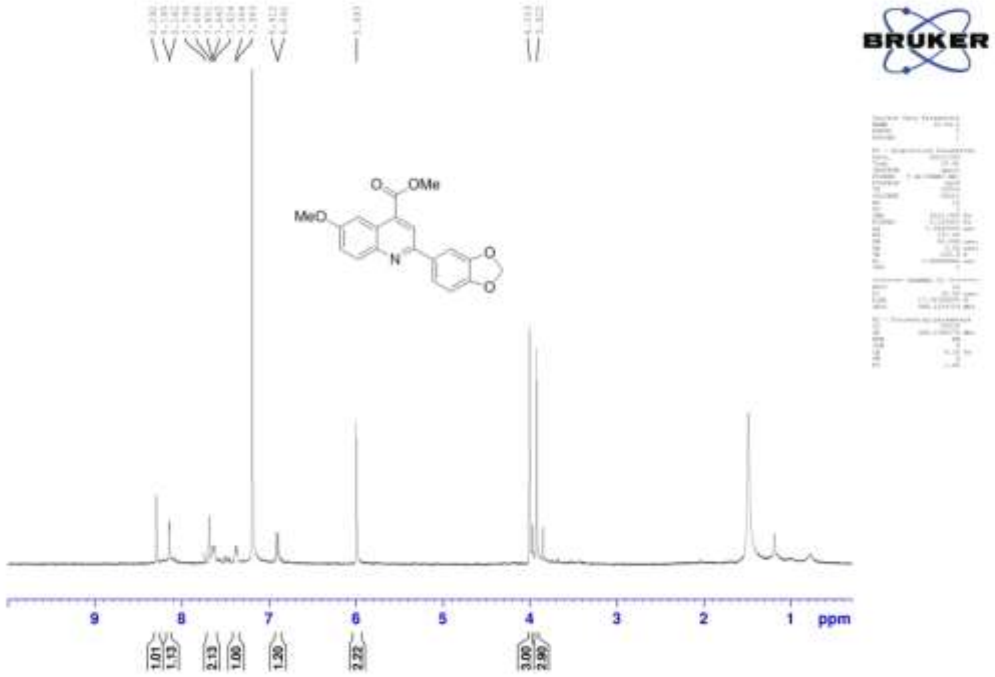

O
OMe
MeO
N
O
O
BRUKER
9
8
7
6
5
4
3
2
1
ppm
1.01
1.13
2.13
1.06
1.20
2.22
3.00
2.90

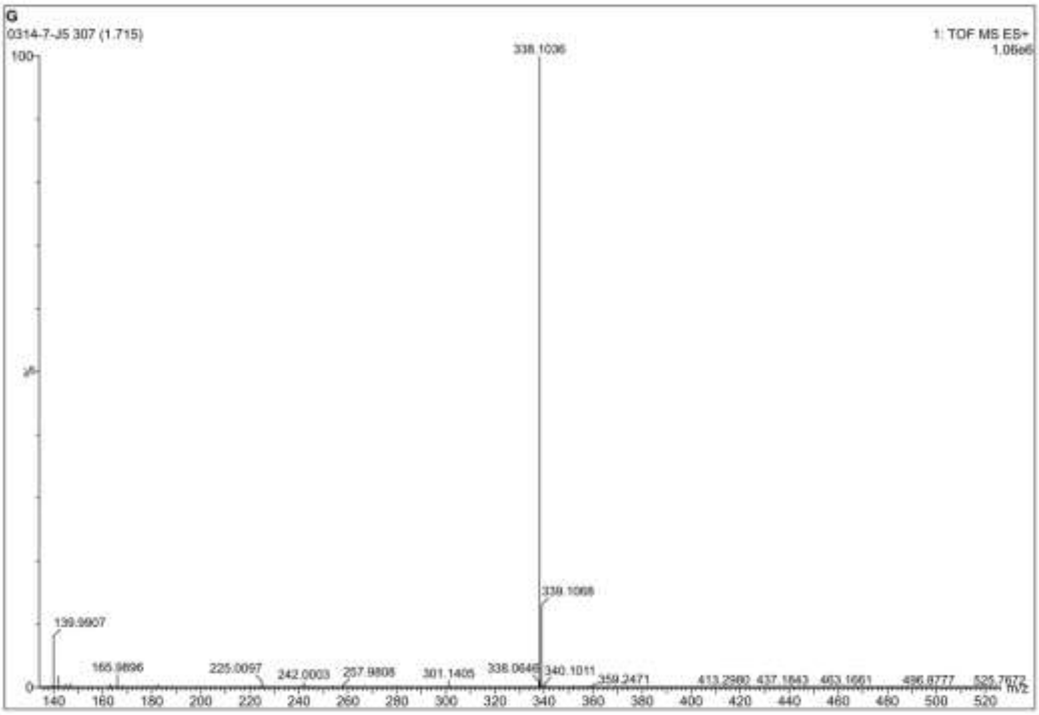

G
0314-7-J5 307 (1.715)
1: TOF MS ES+
1.06e6
100
%
0
338.1036
339.1068
139.9907
165.9896
225.0097
242.0003
257.9808
301.1405
338.0646
340.1011
359.2471
413.2980
437.1843
463.1661
496.8777
525.7672
m/z
140
160
180
200
220
240
260
280
300
320
340
360
380
400
420
440
460
480
500
520

Compound **D2**

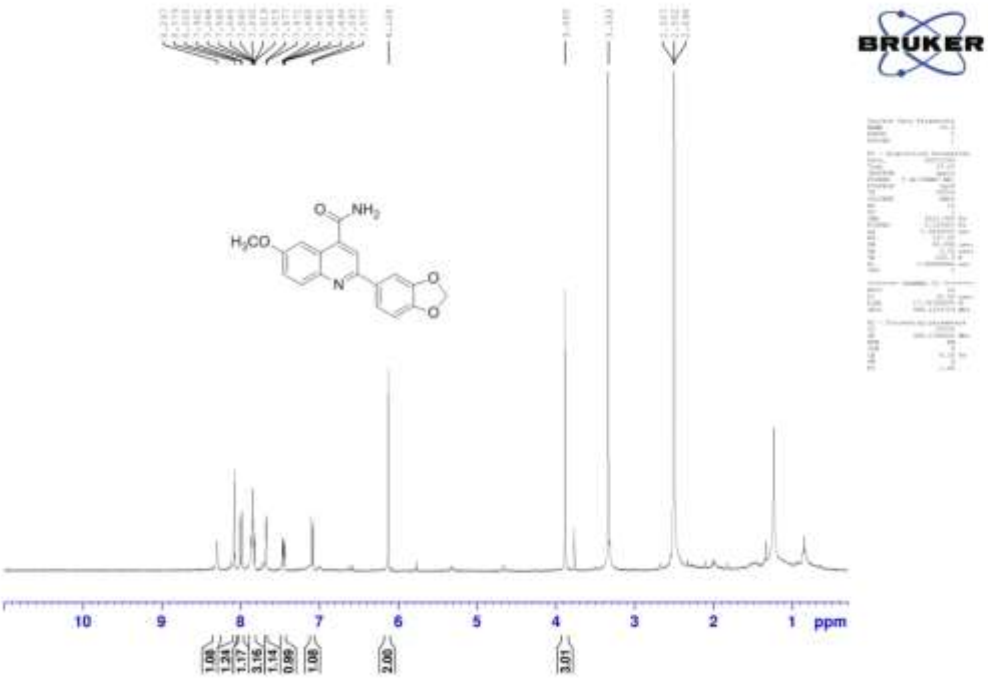

BRUKER
O NH2
H3CO
N
O
O
10 9 8 7 6 5 4 3 2 1 ppm
1.08
1.24
1.17
3.16
1.14
0.99
1.08
2.00
3.01

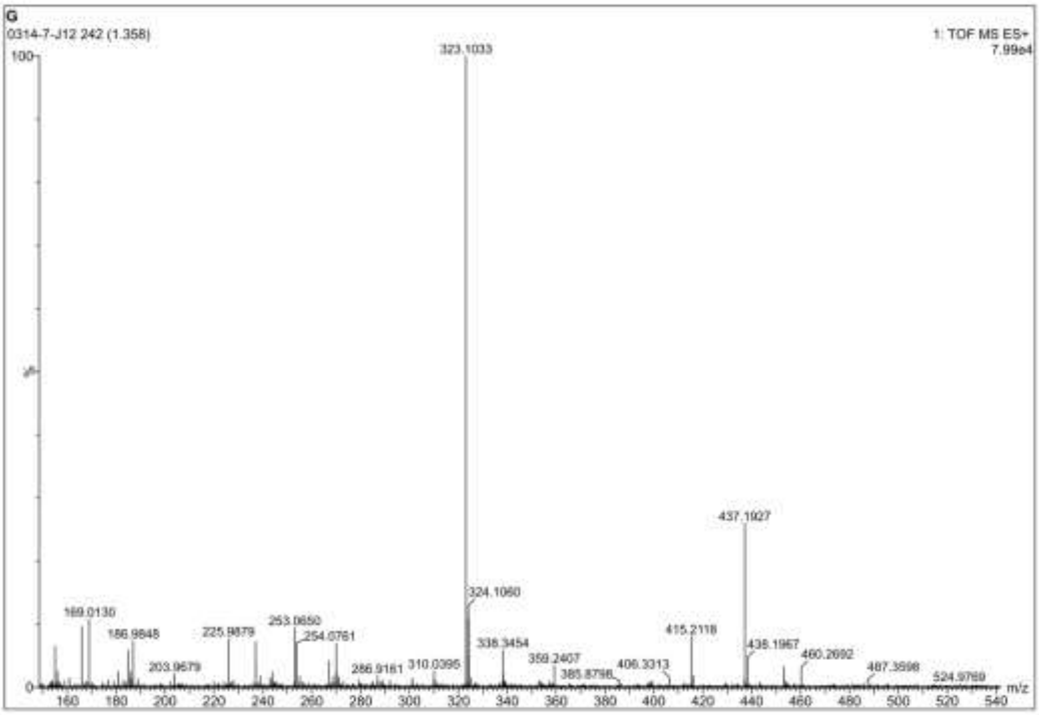

G
0314-7-J12 242 (1.358)
1: TOF MS ES+
7.99e4
100
%
0
323.1033
437.1927
324.1060
169.0130
186.9848
225.9879
253.0650
254.0761
203.9679
286.9161
310.0395
338.3454
359.2407
385.8798
406.3313
415.2118
438.1967
460.2692
487.3598
524.9769
m/z
160 180 200 220 240 260 280 300 320 340 360 380 400 420 440 460 480 500 520 540

Compound **D3**

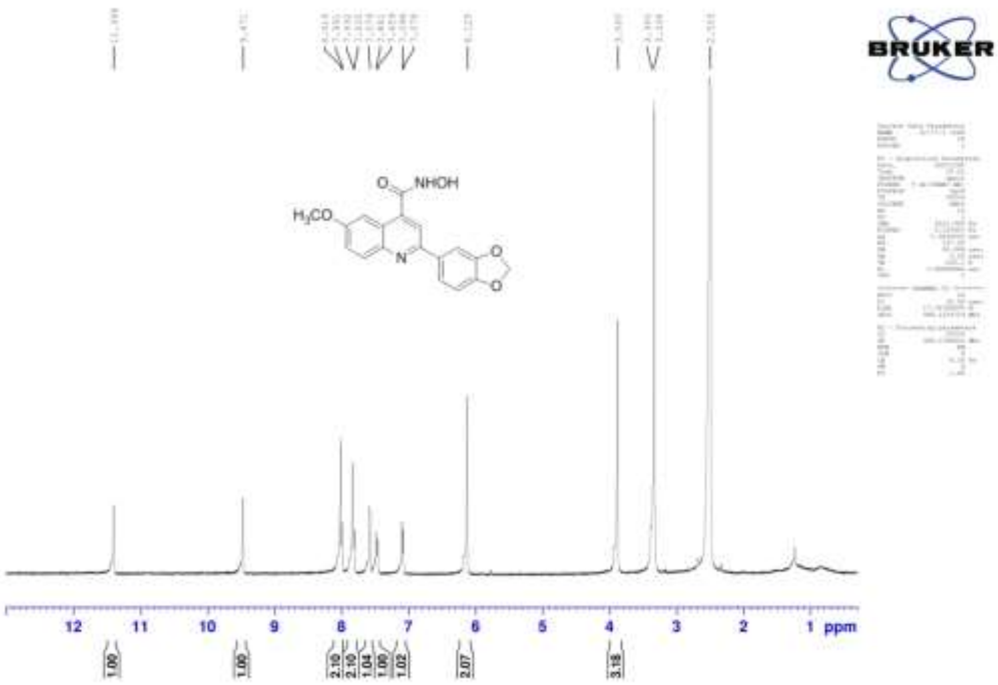

O NHOH
H3CO
N
O
O
BRUKER
12 11 10 9 8 7 6 5 4 3 2 1 ppm
1.00
1.00
2.10 2.10 1.04 1.00 1.02
2.07
3.18

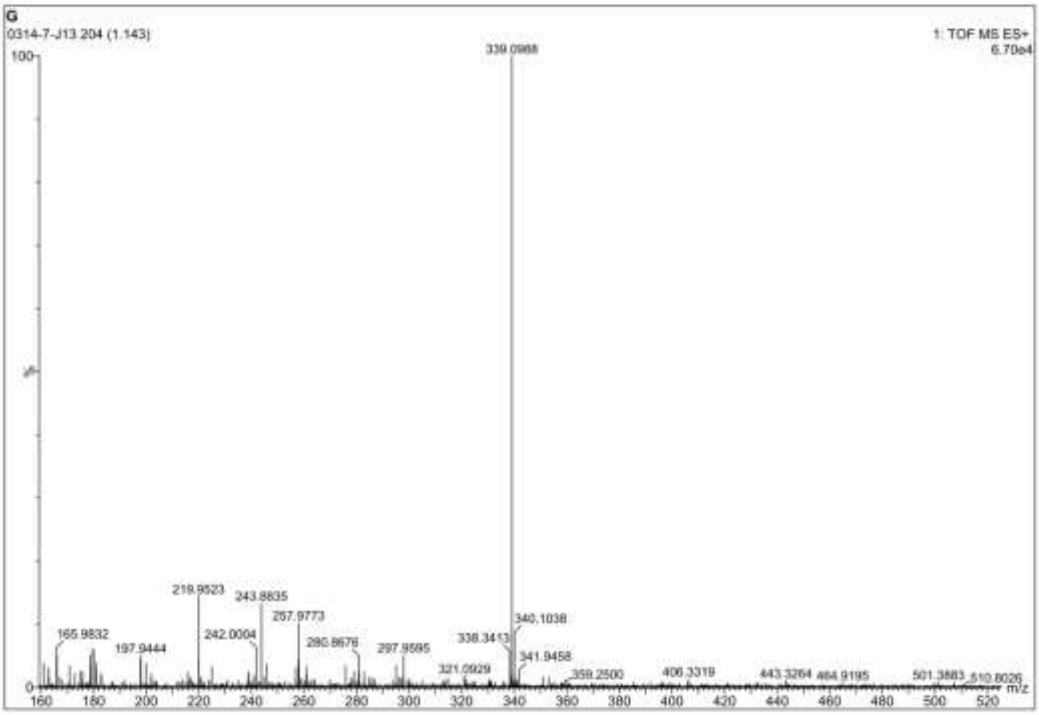

G
0314-7-J13 204 (1.143)
1: TOF MS ES+
6.70e4
339.0988
100
%
0
219.9523
243.8835
257.9773
340.1038
165.9832
242.0004
280.8676
297.9595
338.3413
197.9444
341.9458
321.0929
359.2500
406.3319
443.3264
464.9195
501.3883
510.8026
m/z
160 180 200 220 240 260 280 300 320 340 360 380 400 420 440 460 480 500 520

Compound **F1**

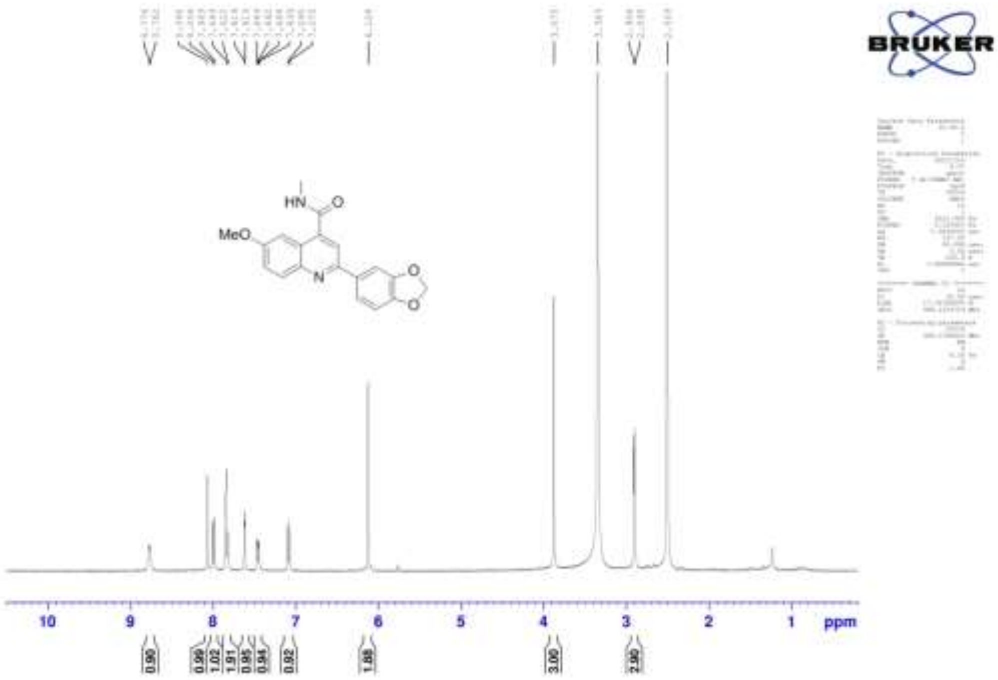
MeO
HN
O
N
ppm
0.90
0.99
1.02
1.91
0.95
0.94
0.92
1.88
3.00
2.90

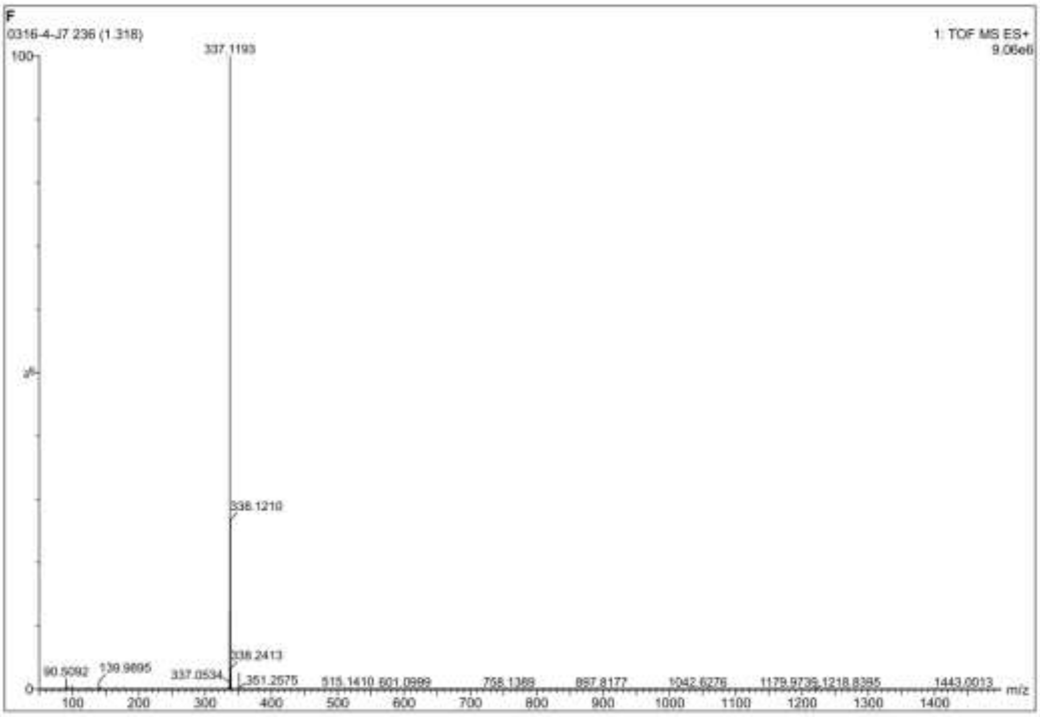
F
0316-4-J7 236 (1.318)
1: TOF MS ES+
9.06e6
337.1193
338.1210
338.2413
90.5092
139.9895
337.0534
351.2575
515.1410
601.0989
758.1389
887.8177
1042.6276
1179.9739
1218.8395
1443.0013
m/z

Compound **F2**

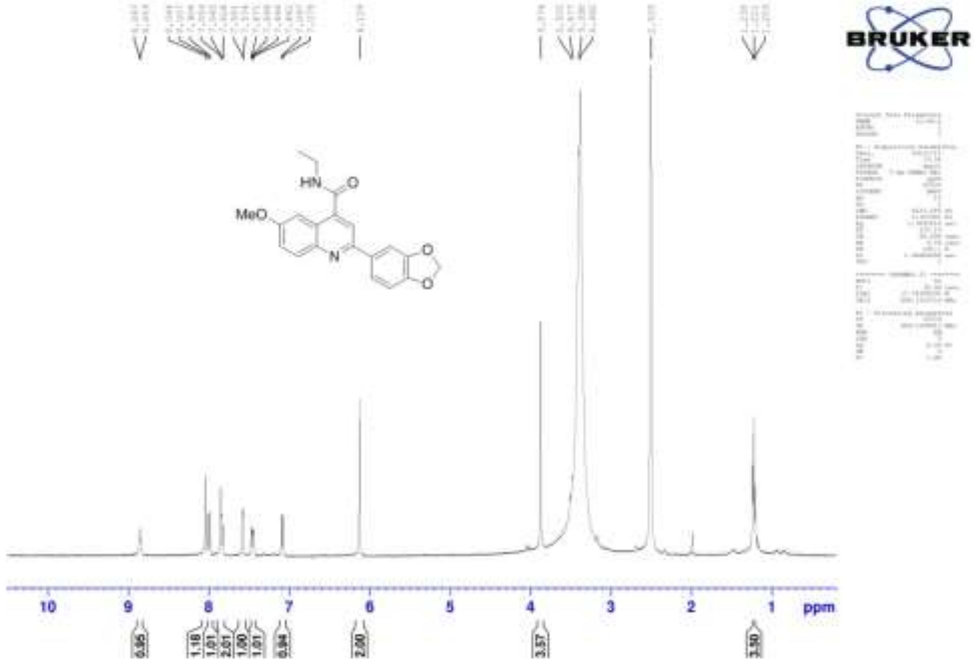
HN
O
MeO
N
O
O
BRUKER
10 9 8 7 6 5 4 3 2 1 ppm
0.95
1.10
1.01
2.01
1.00
1.01
0.94
2.00
3.57
3.50

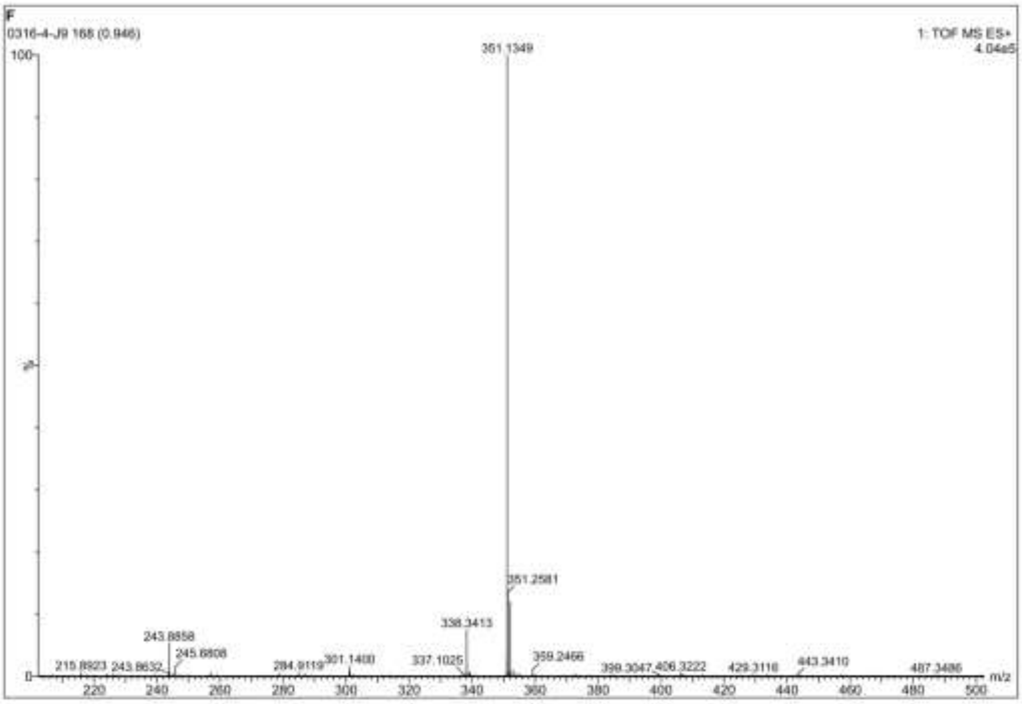
F
0316-4-J9 168 (0.946)
1: TOF MS ES+
4.04e5
351.1349
100
%
0
351.2581
338.3413
243.8858
245.6808
215.8923
243.8632
284.9119
301.1400
337.1025
359.2466
399.3047
406.3222
429.3116
443.3410
487.3486
220 240 260 280 300 320 340 360 380 400 420 440 460 480 500
m/z

Compound **F3**

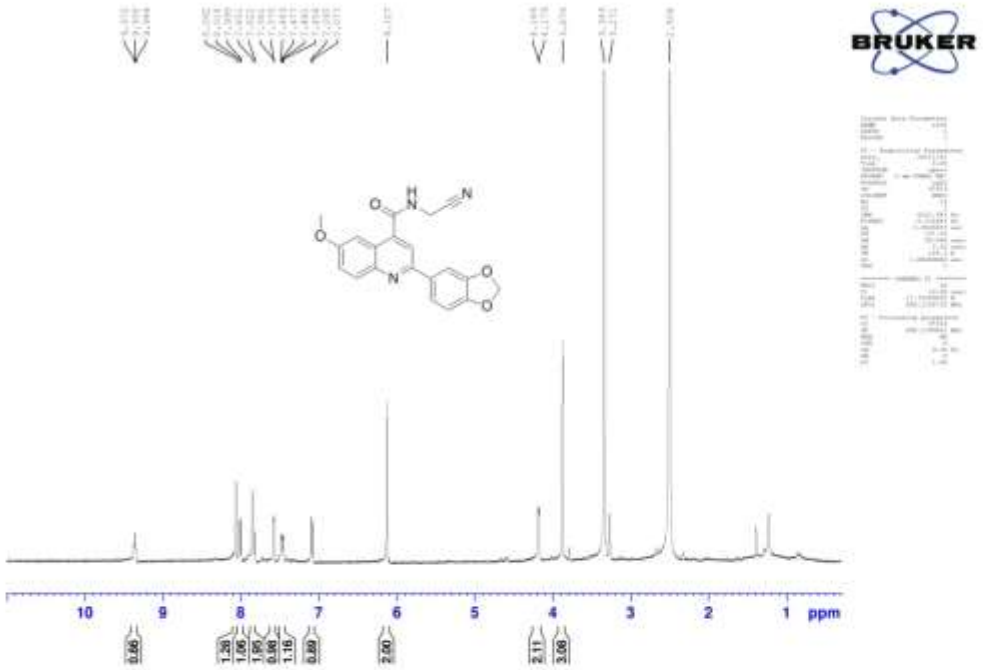

BRUKER
10
9
8
7
6
5
4
3
2
1
ppm
0.66
1.28
1.06
1.95
0.98
1.16
0.89
2.00
2.11
3.06

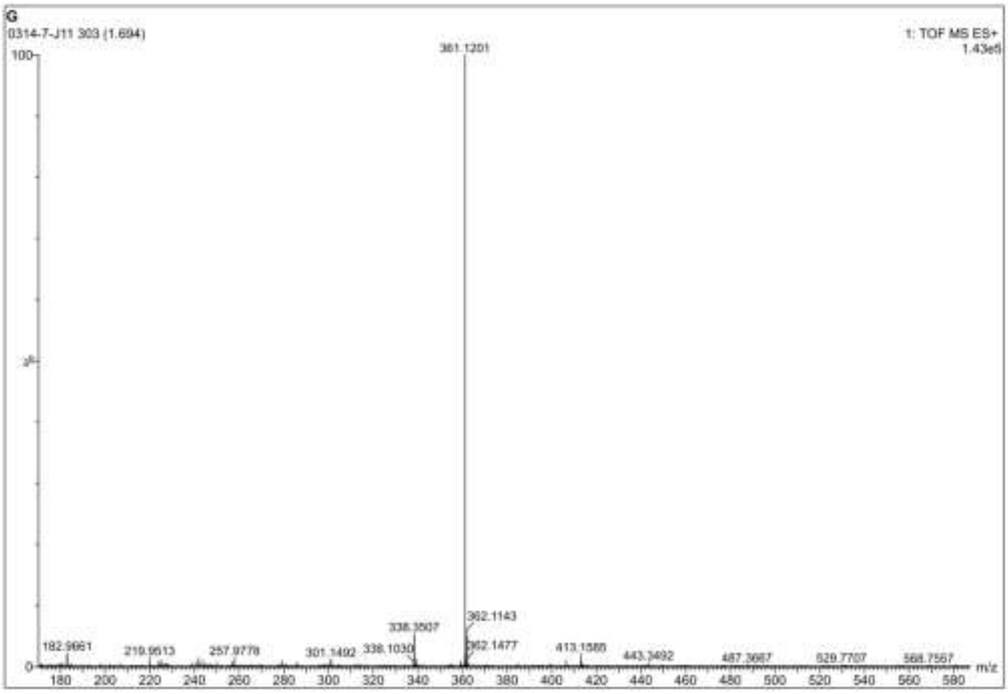

G
0314-7-J11 303 (1.694)
1: TOF MS ES+
1.43e5
100
%
0
361.1201
362.1143
338.3507
362.1477
182.9661
219.9513
257.9778
301.1492
338.1030
413.1585
443.3492
487.3667
529.7707
568.7567
m/z

Compound **F4**

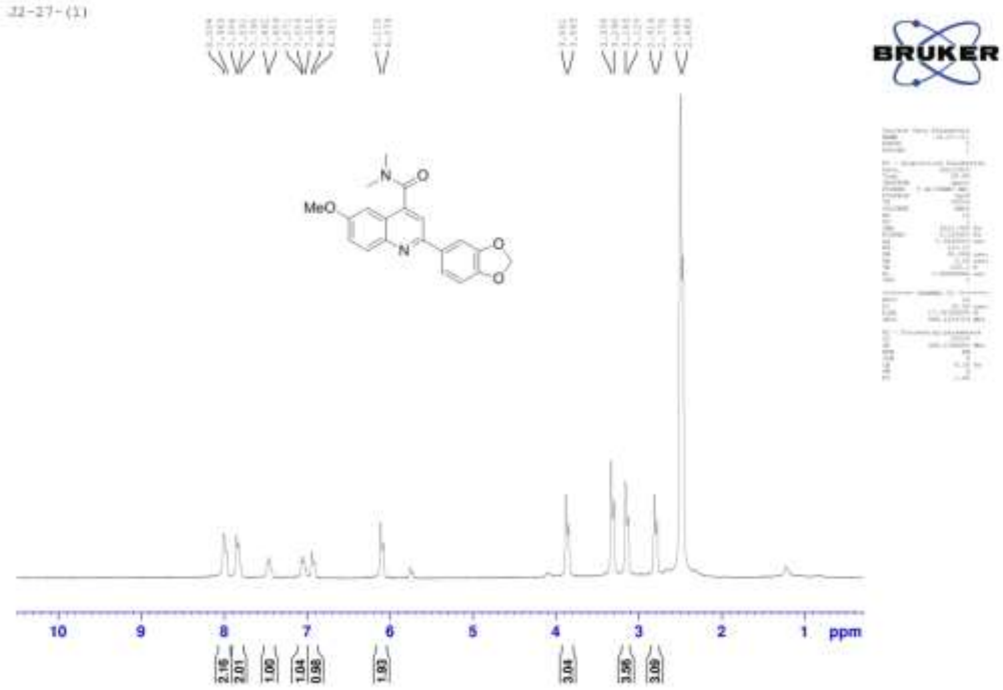

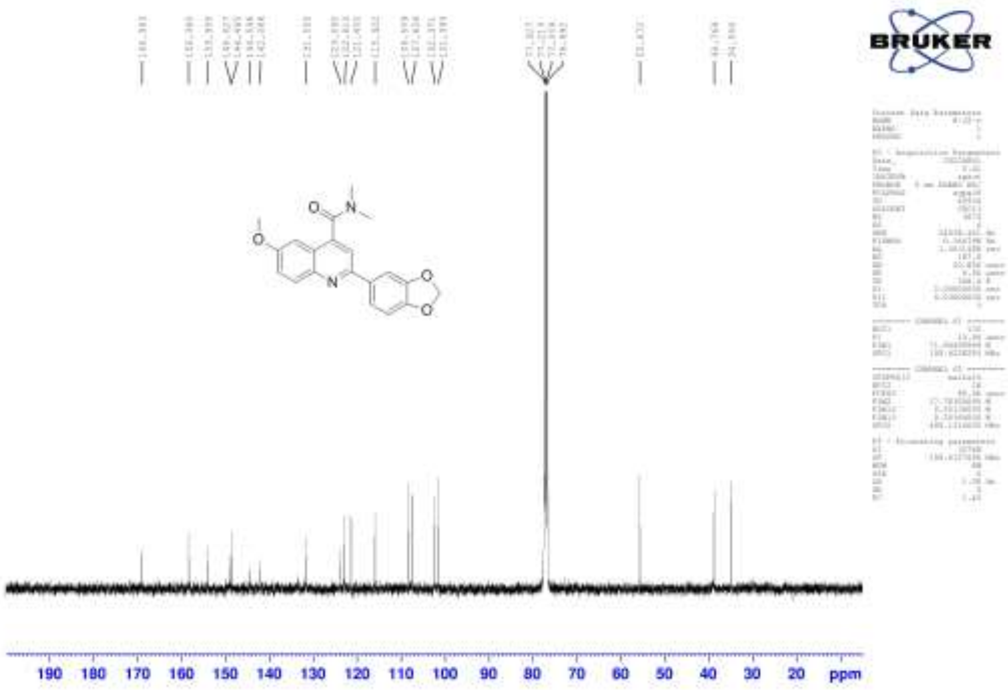

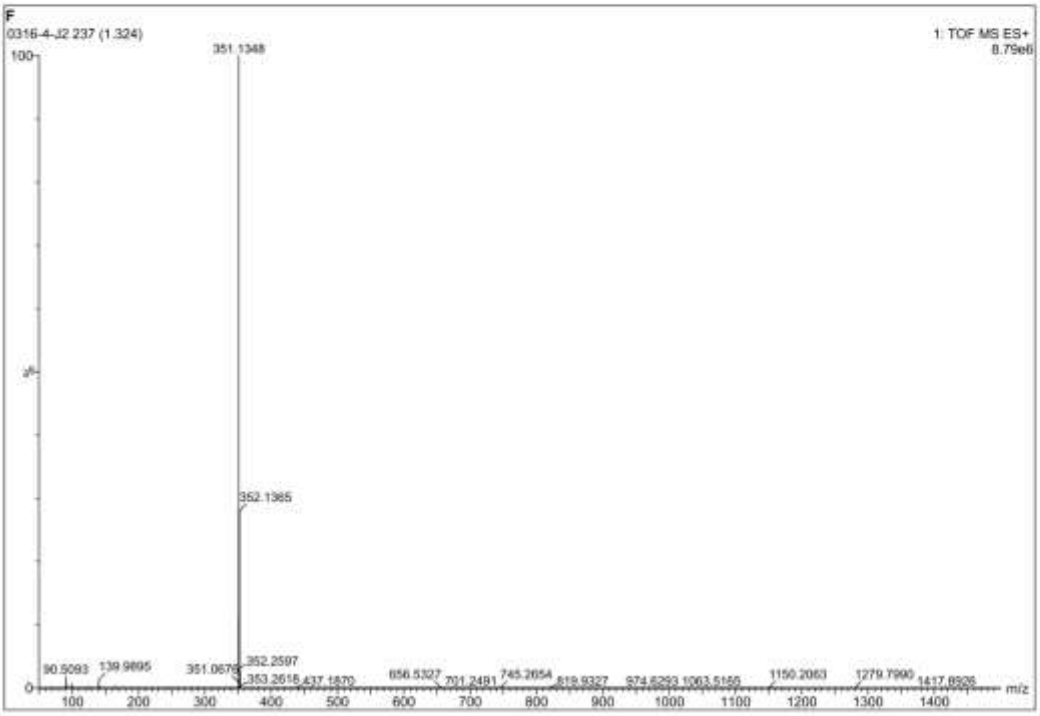
F
0316-4-J2 237 (1.324)
1: TOF MS ES+
8.79e6
100
%
0
351.1348
352.1365
352.2597
351.0678
353.2618
90.5093
139.9895
437.1870
656.5327
701.2491
745.2654
819.9327
974.6293
1063.5165
1150.2063
1279.7990
1417.8926
m/z
100
200
300
400
500
600
700
800
900
1000
1100
1200
1300
1400

Compound **F5**

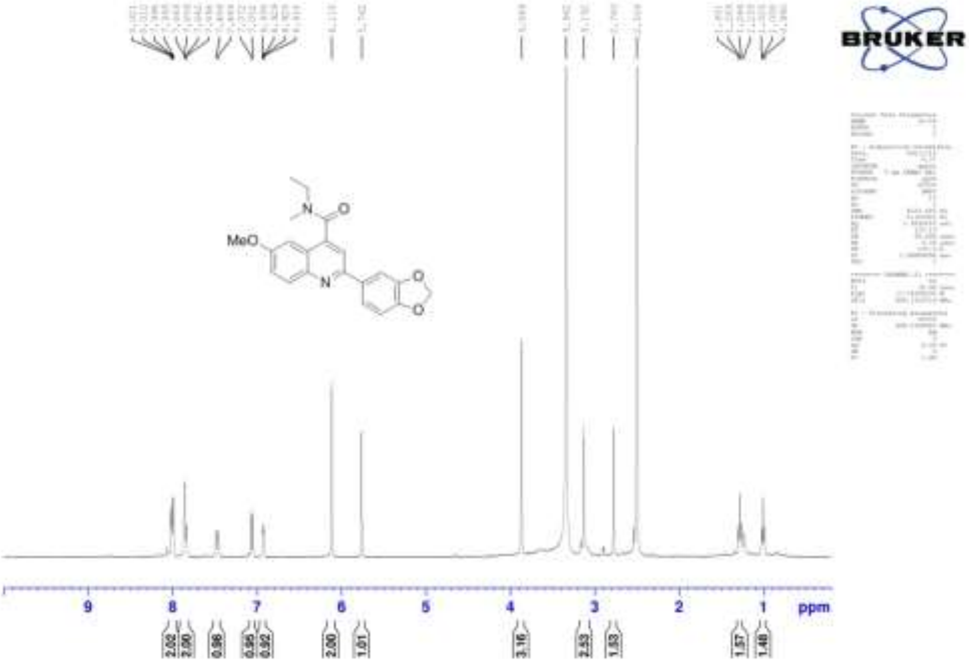
MeO
N
O
BRUKER
9
8
7
6
5
4
3
2
1
ppm
2.02
2.00
0.96
0.95
0.92
2.00
1.01
3.16
2.53
1.53
1.57
1.48

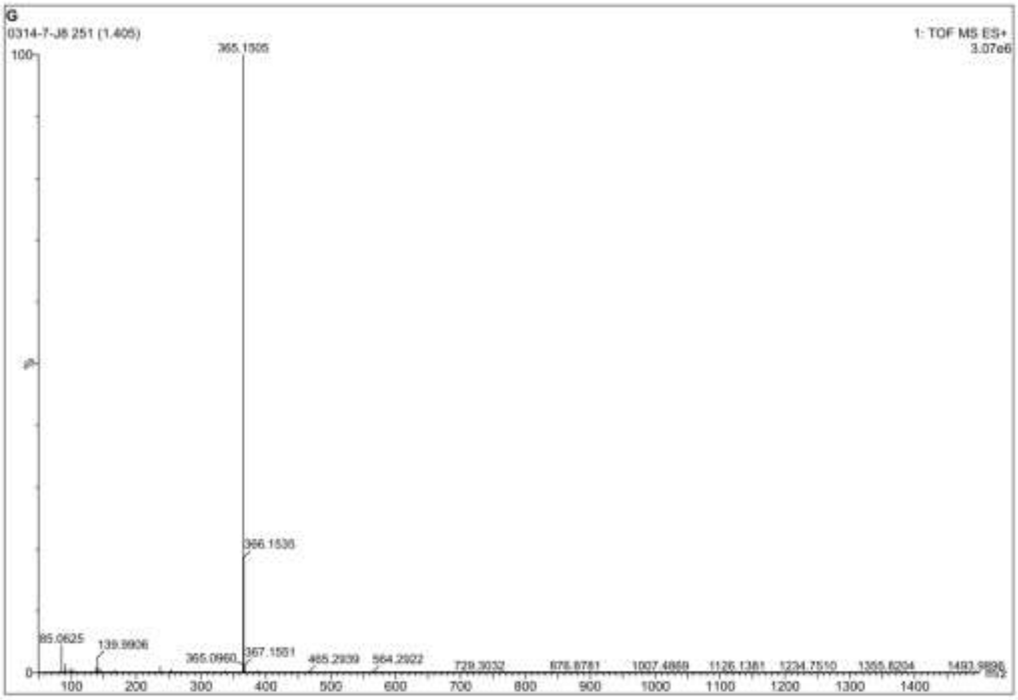
G
0314-7-J8 251 (1.405)
1: TOF MS ES+
3.07e6
100
%
0
365.1505
366.1536
85.0625
139.9906
365.0960
367.1551
465.2939
564.2922
729.3032
876.8781
1007.4869
1126.1381
1234.7510
1355.8204
1493.9896
m/z
100
200
300
400
500
600
700
800
900
1000
1100
1200
1300
1400

Compound **F6**

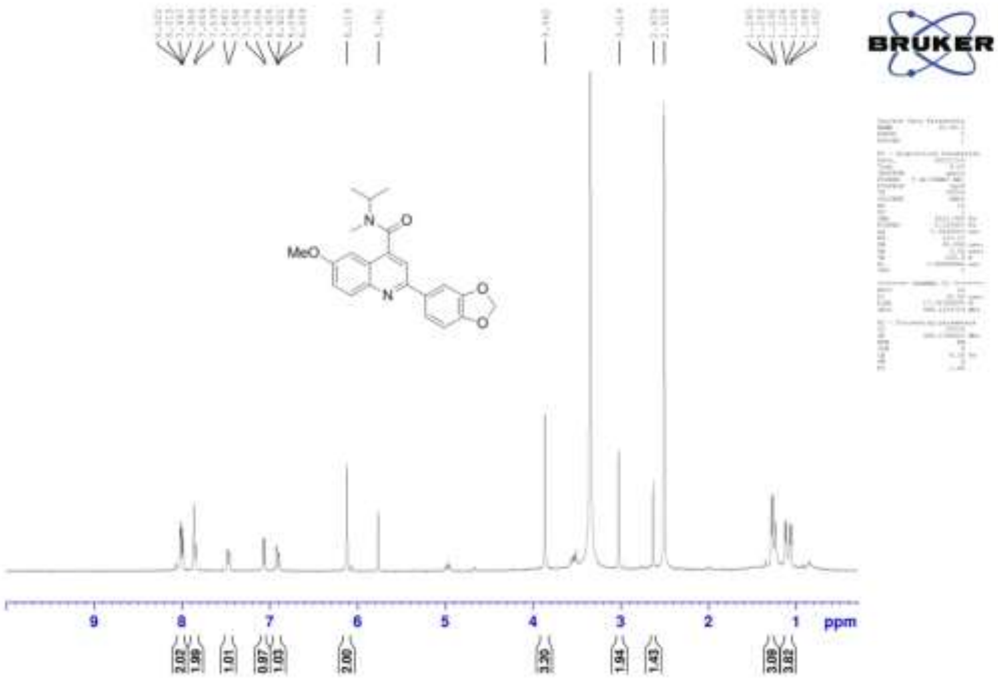
MeO
N
O
ppm
9
8
7
6
5
4
3
2
1
2.02
1.99
1.01
0.97
1.03
2.00
3.20
1.94
1.43
3.09
3.82

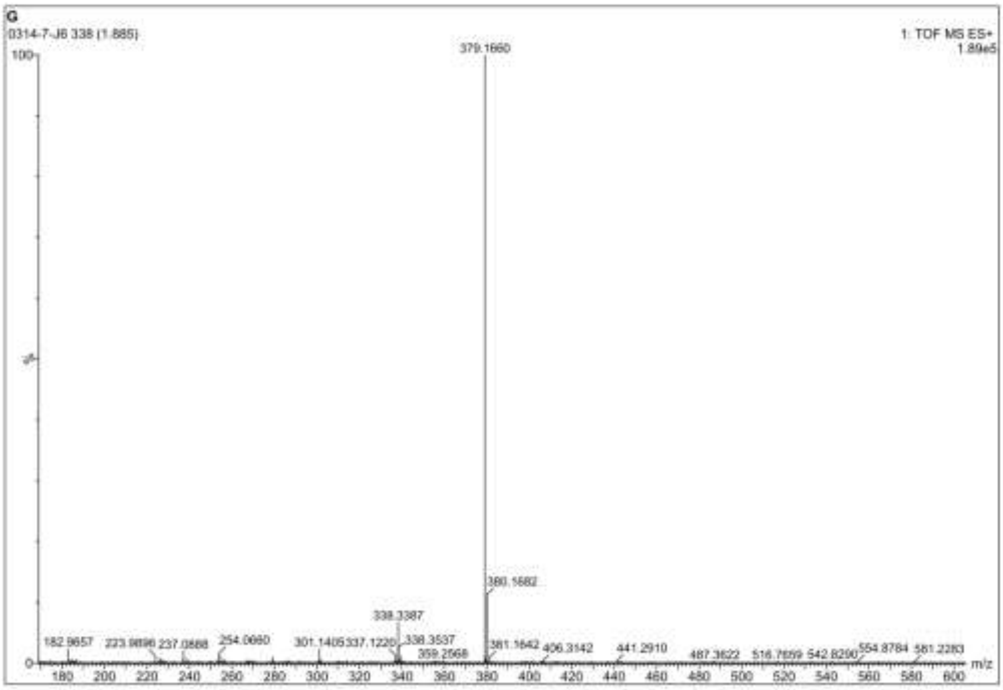
G
0314-7-J6 338 (1.885)
1: TOF MS ES+
1.89e5
100
%
0
379.1660
380.1682
338.3387
182.9657
223.9896
237.0888
254.0660
301.1405
337.1220
338.3537
359.2568
381.1642
406.3142
441.2910
487.3622
516.7659
542.8290
554.8784
581.2283
m/z
180
200
220
240
260
280
300
320
340
360
380
400
420
440
460
480
500
520
540
560
580
600

Compound **F7**

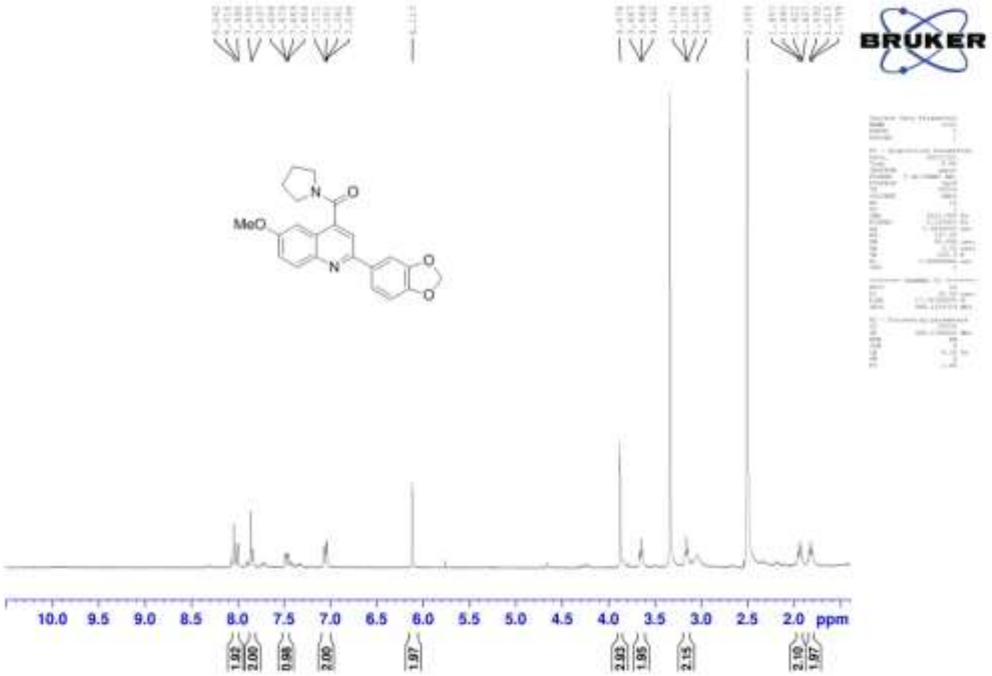
MeO
N
O
BRUKER
10.0 9.5 9.0 8.5 8.0 7.5 7.0 6.5 6.0 5.5 5.0 4.5 4.0 3.5 3.0 2.5 2.0 ppm
1.92 2.00 0.98 2.00 1.97 2.93 1.95 2.15 2.10 1.97

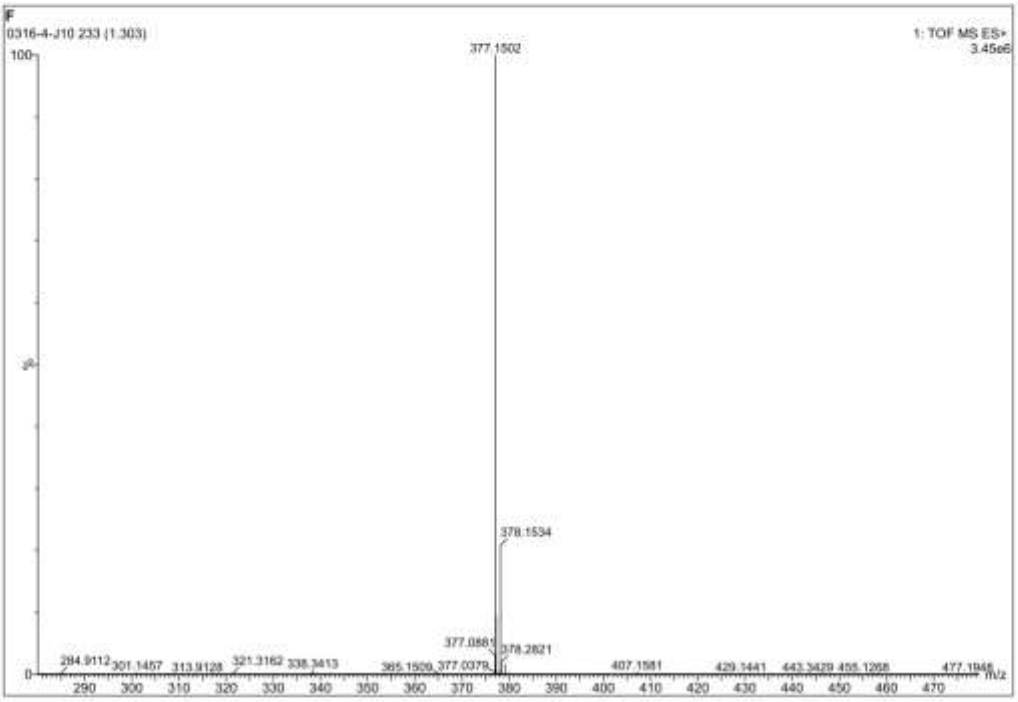
F
0316-4-J10 233 (1.303)
1: TOF MS ES+
3.45e6
100
%
0
377.1502
378.1534
377.0881
378.2821
284.9112 301.1457 313.9128 321.3162 338.3413 365.1509 377.0379 407.1581 429.1441 443.3429 455.1268 477.1946
290 300 310 320 330 340 350 360 370 380 390 400 410 420 430 440 450 460 470
m/z

Compound **G1**

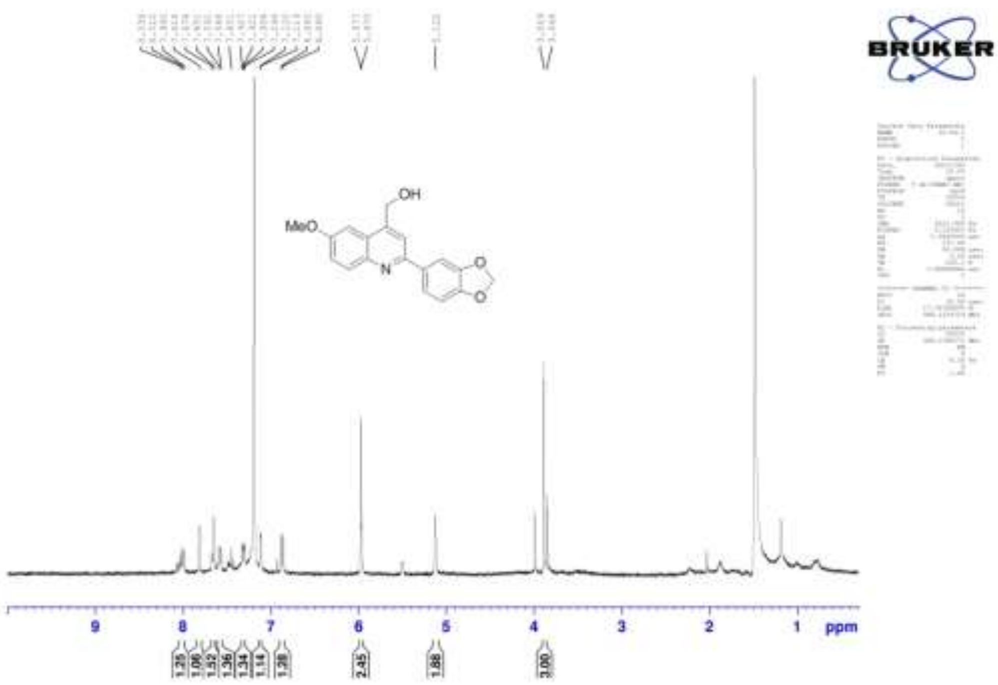

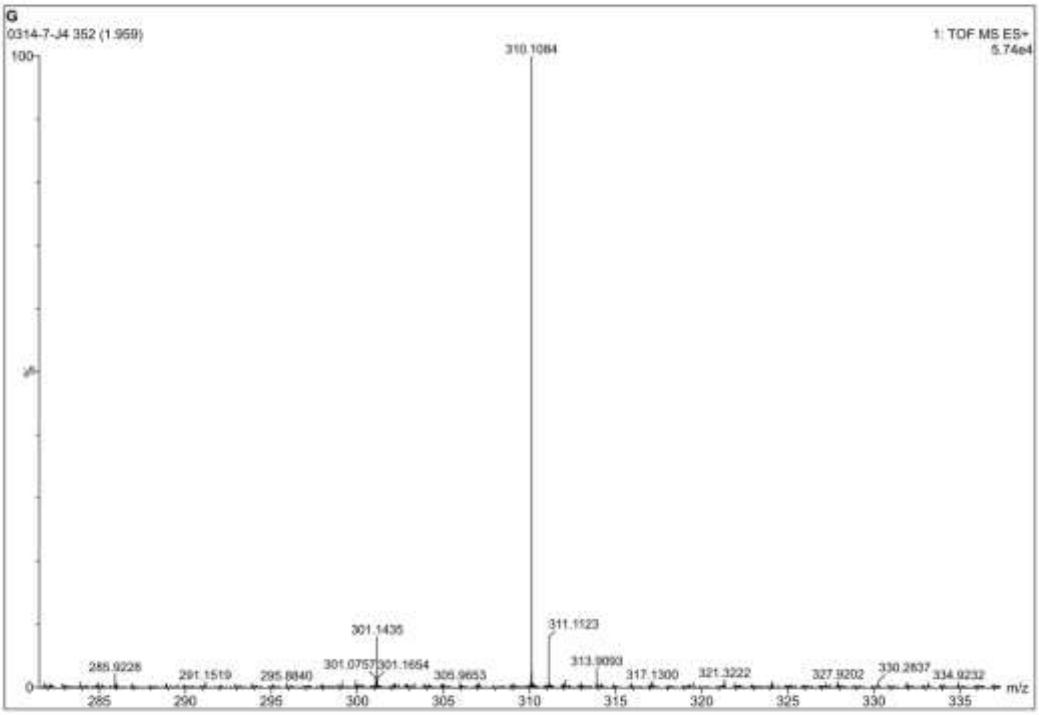
G
0314-7-J4 352 (1.959)
1: TOF MS ES+
5.74e4
310.1084
311.1123
301.1436
285.9228
291.1519
295.8840
301.0757
301.1654
305.9653
313.9093
317.1300
321.3222
327.9202
330.2837
334.9232
m/z

Compound **G2**

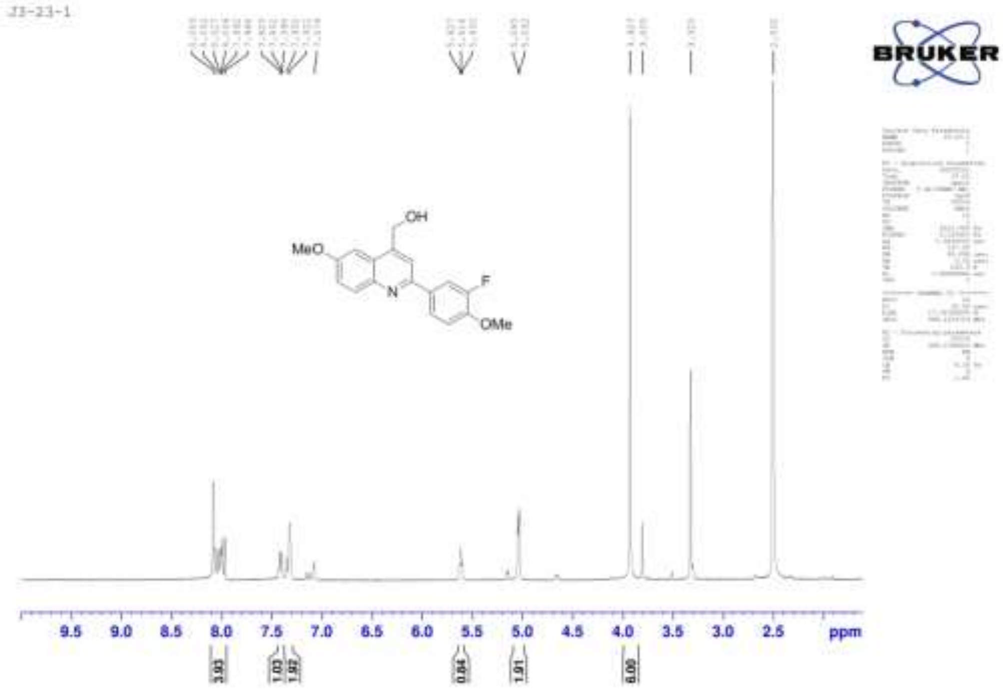

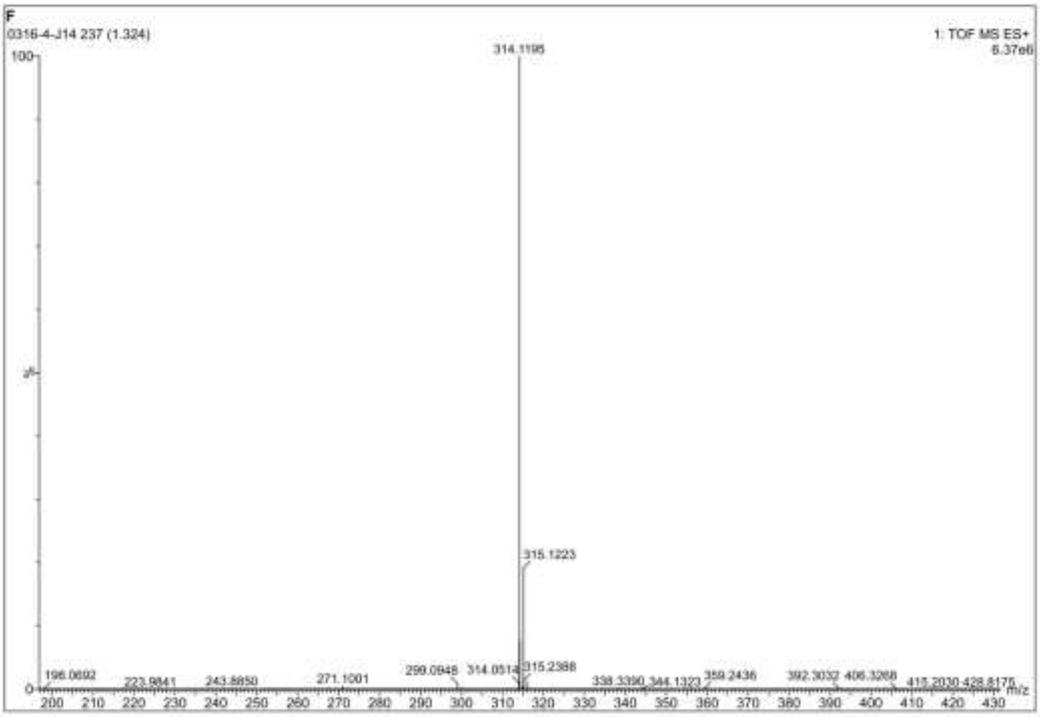

Compound **G3**

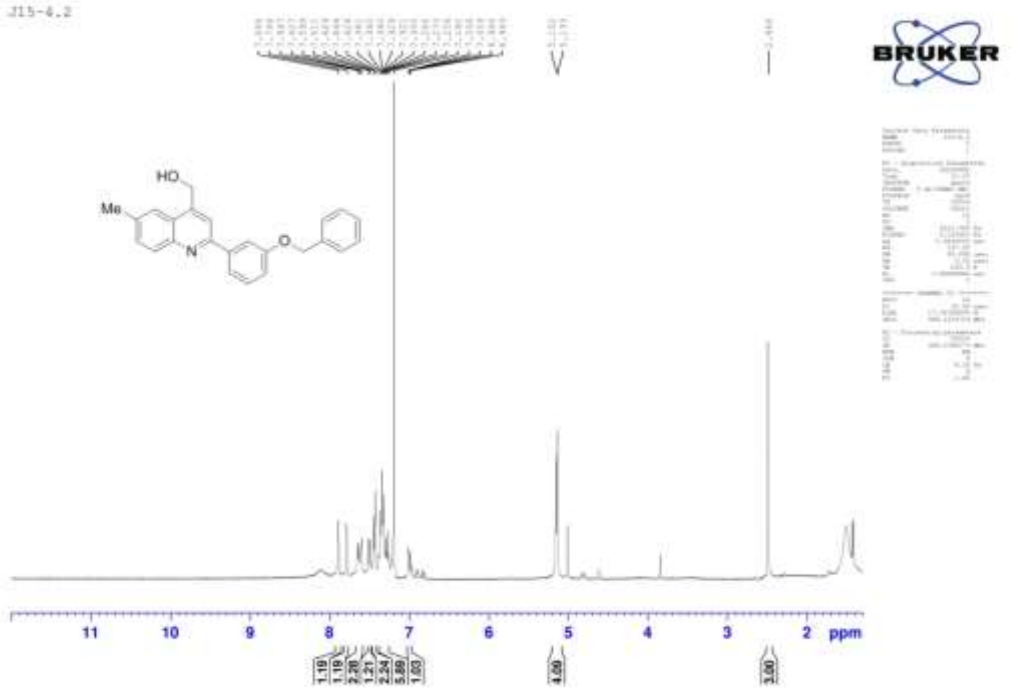

J15-4.2
BRUKER
HO
Me
N
O
1.19
1.19
2.26
1.21
2.34
5.89
1.03
4.09
3.00
11 10 9 8 7 6 5 4 3 2 ppm

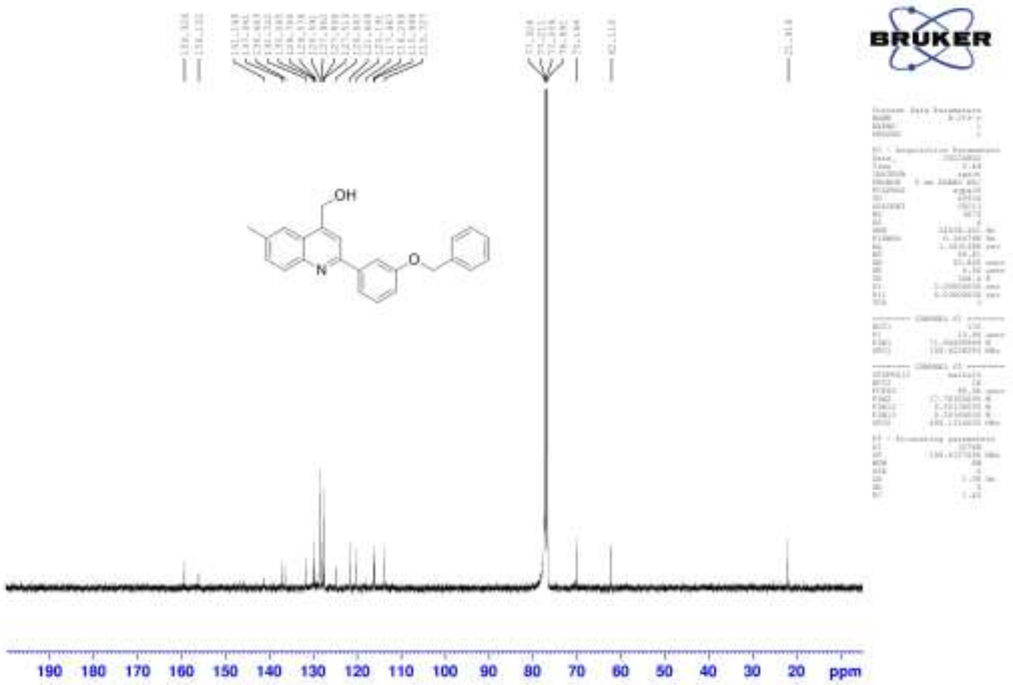

BRUKER
OH
N
O
190 180 170 160 150 140 130 120 110 100 90 80 70 60 50 40 30 20 ppm

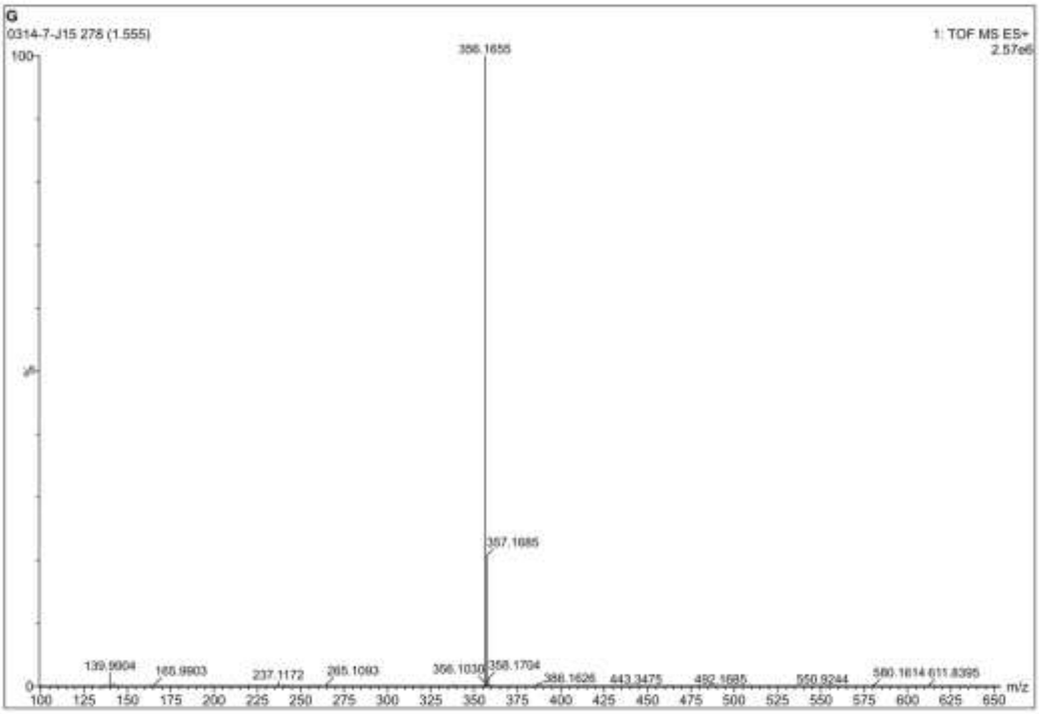
G
0314-7-J15 278 (1.555)
1: TOF MS ES+
2.57e6
356.1655
357.1685
139.9904
165.9903
237.1172
265.1093
356.1030
358.1704
386.1626
443.3475
492.1685
550.9244
580.1614
611.8395
%
m/z
100
0
100 125 150 175 200 225 250 275 300 325 350 375 400 425 450 475 500 525 550 575 600 625 650

Compound **G4**

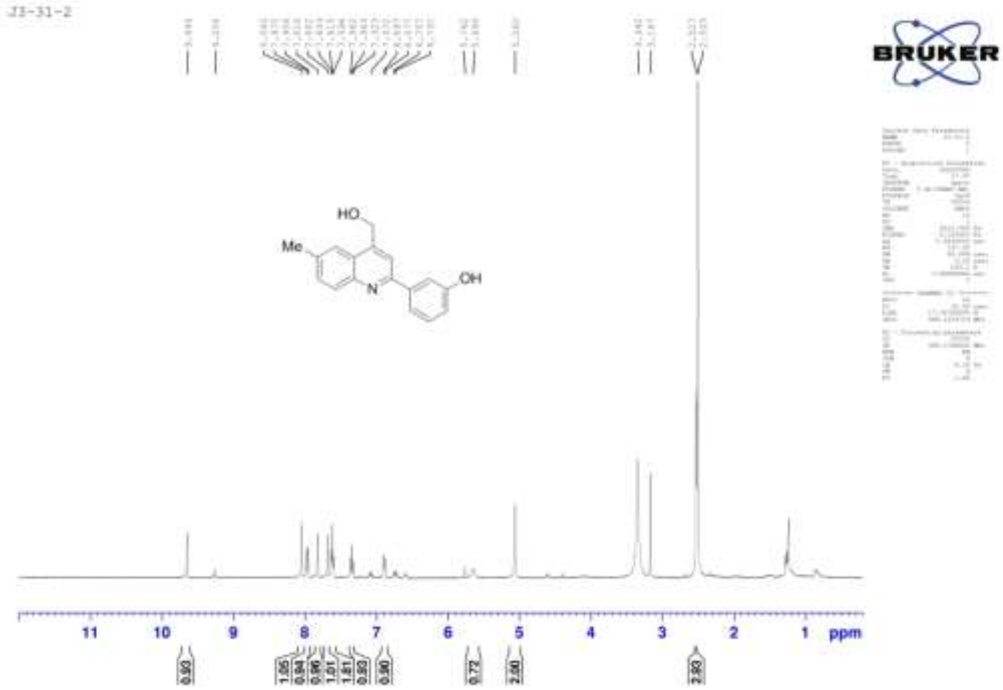

J3-31-2
HO
Me
N
OH
BRUKER
11 10 9 8 7 6 5 4 3 2 1 ppm

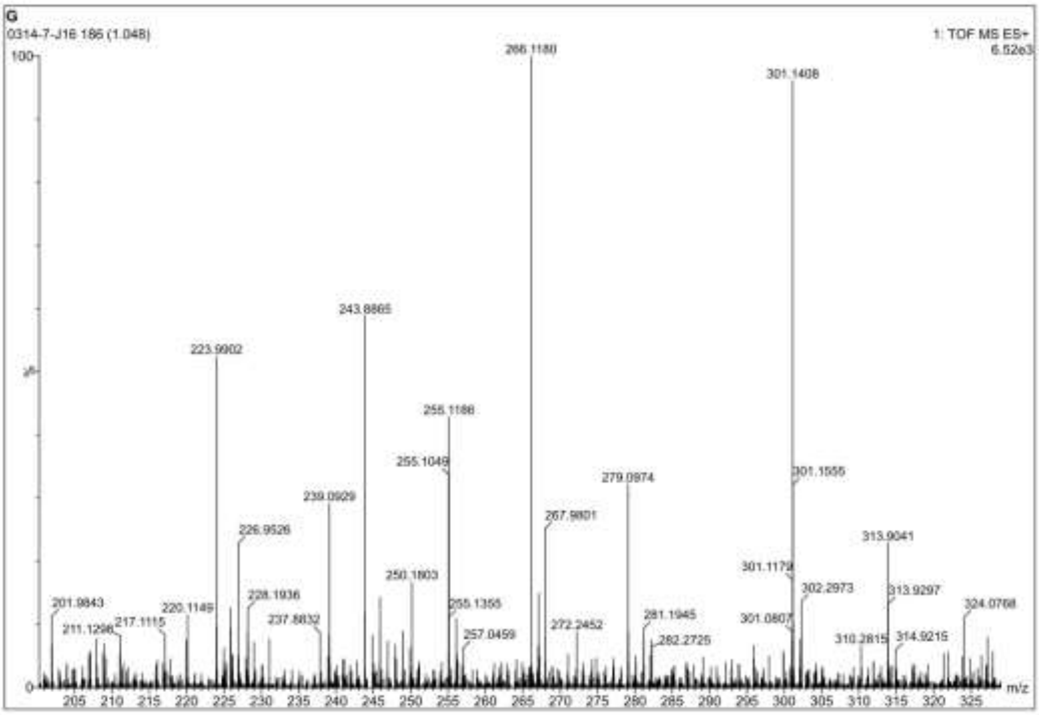

G
0314-7-J16 186 (1.048)
1: TOF MS ES+
6.52e3
266.1180
301.1408
243.8865
223.9902
256.1186
255.1049
301.1555
279.0974
239.0929
267.9801
226.9526
313.9041
301.1179
250.1803
302.2973
313.9297
228.1936
201.9843
220.1149
255.1355
324.0768
217.1115
211.1296
237.8832
281.1945
301.0807
272.2452
257.0459
310.2815
314.9215
282.2725
m/z

Compound **G5**

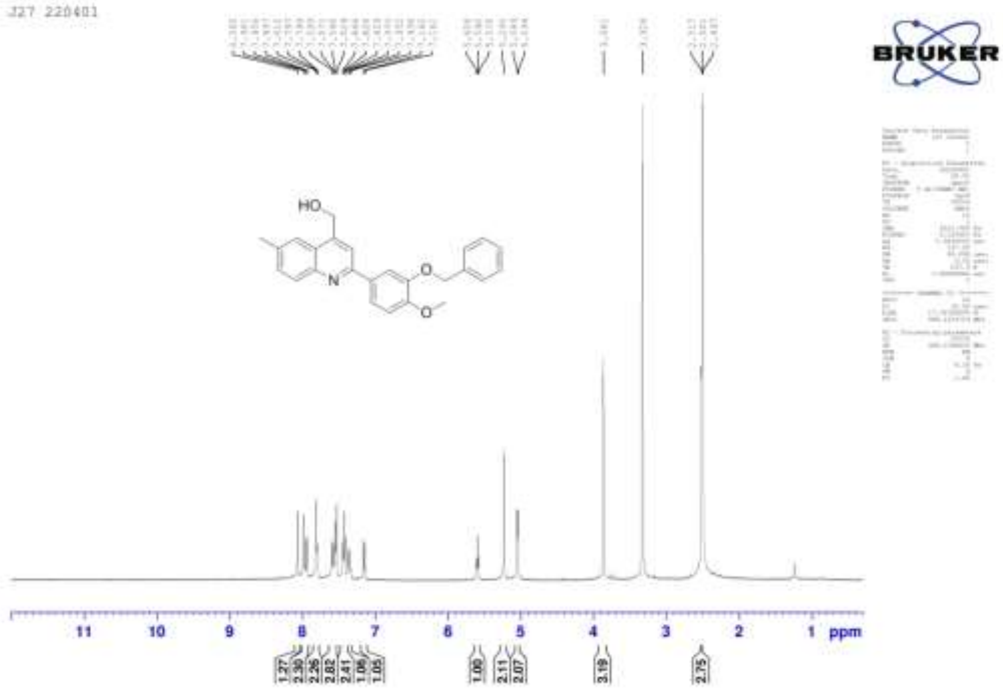
J27 220401
BRUKER
HO
N
O
O
11
10
9
8
7
6
5
4
3
2
1
ppm
1.27
2.30
2.26
2.82
2.41
1.06
1.05
1.00
2.11
2.07
3.19
2.75

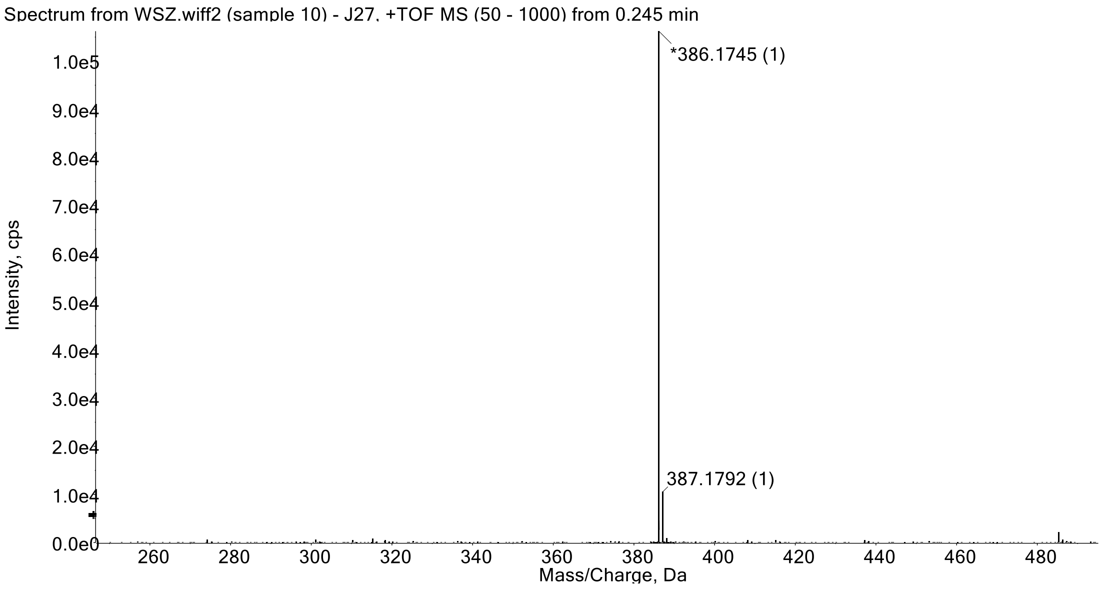
Spectrum from WSZ.wiff2 (sample 10) - J27, +TOF MS (50 - 1000) from 0.245 min
*386.1745 (1)
387.1792 (1)
Intensity, cps
1.0e5
9.0e4
8.0e4
7.0e4
6.0e4
5.0e4
4.0e4
3.0e4
2.0e4
1.0e4
0.0e0
260
280
300
320
340
360
380
400
420
440
460
480
Mass/Charge, Da

Compound **G6**

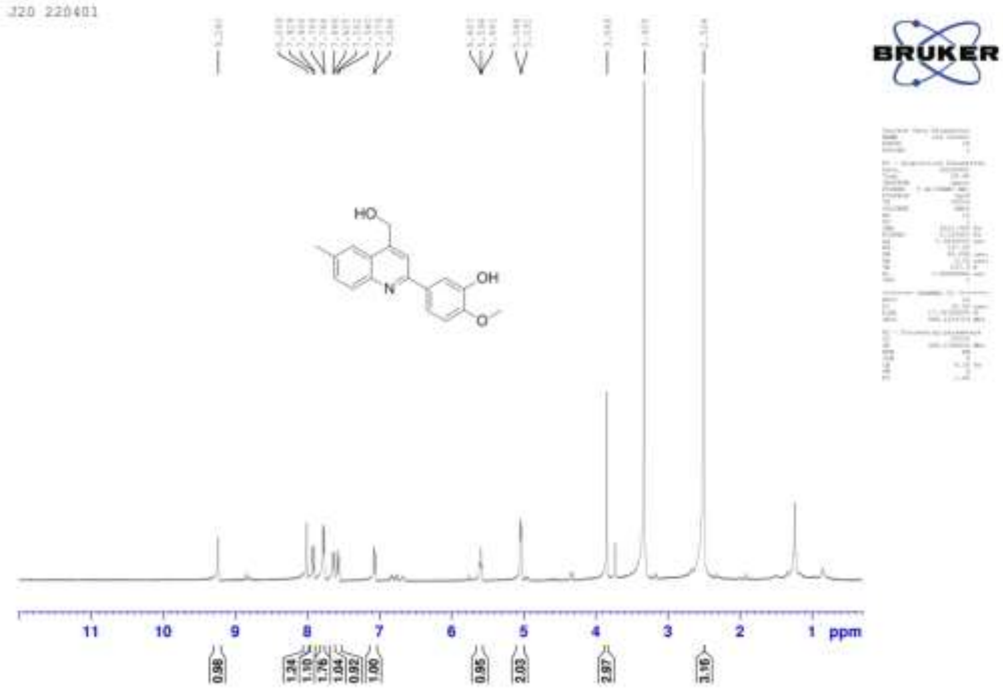
J20 220401
BRUKER
HO
OH
N
O
11 10 9 8 7 6 5 4 3 2 1 ppm
0.98
1.24
1.10
1.76
1.04
0.92
1.00
0.95
2.03
2.97
3.16

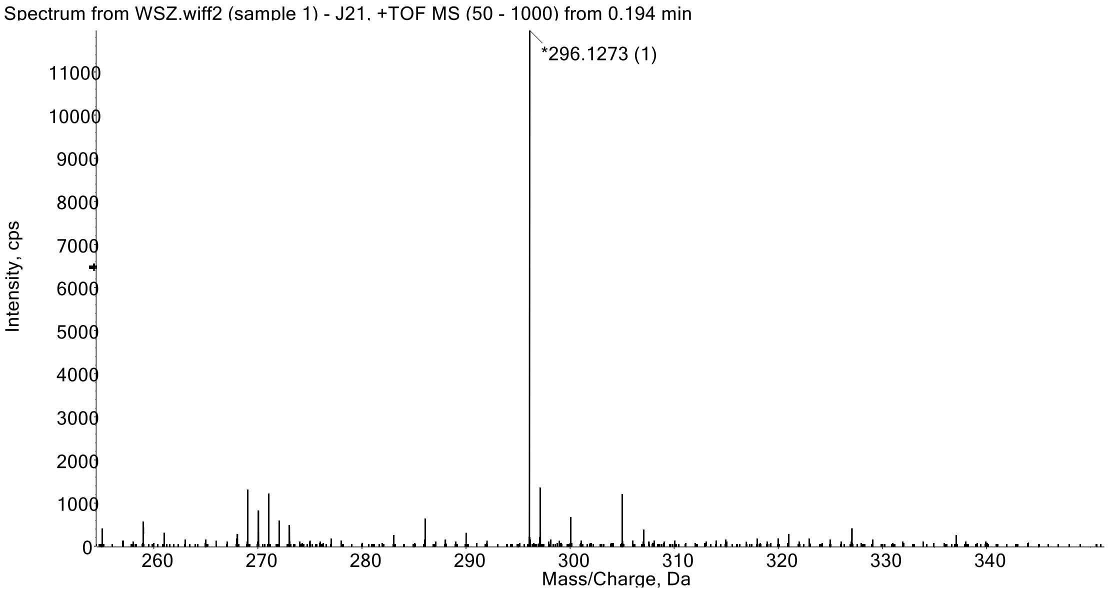
Spectrum from WSZ.wiff2 (sample 1) - J21, +TOF MS (50 - 1000) from 0.194 min
*296.1273 (1)
Intensity, cps
0
1000
2000
3000
4000
5000
6000
7000
8000
9000
10000
11000
260
270
280
290
300
310
320
330
340
Mass/Charge, Da

Compound **G7**

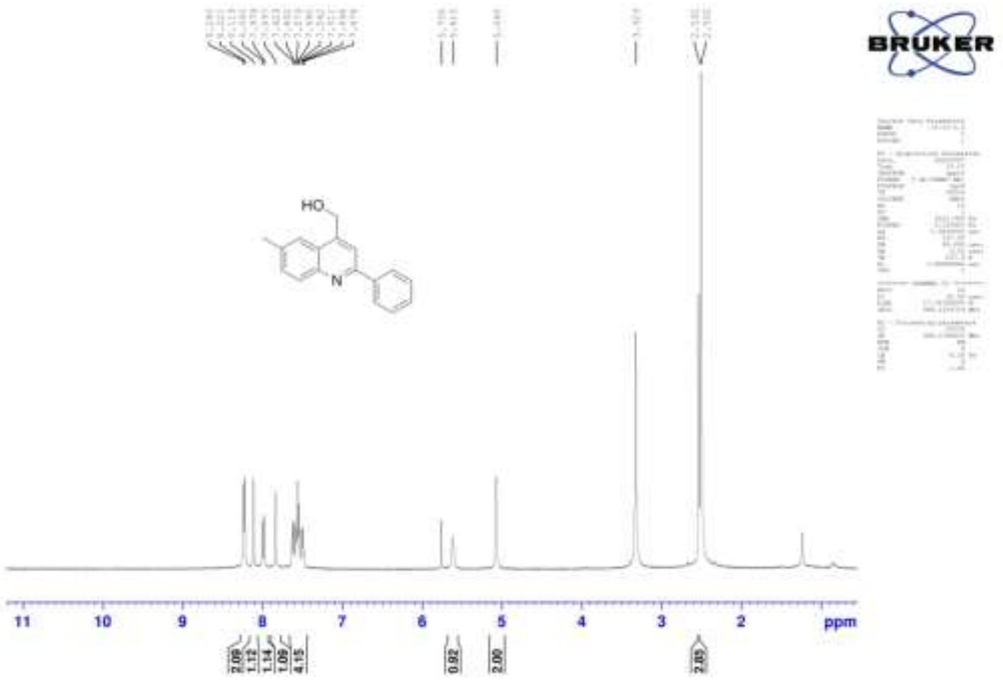
BRUKER
HO
N
11 10 9 8 7 6 5 4 3 2 ppm
2.09 1.12 1.14 1.09 4.15
0.92
2.00
2.85

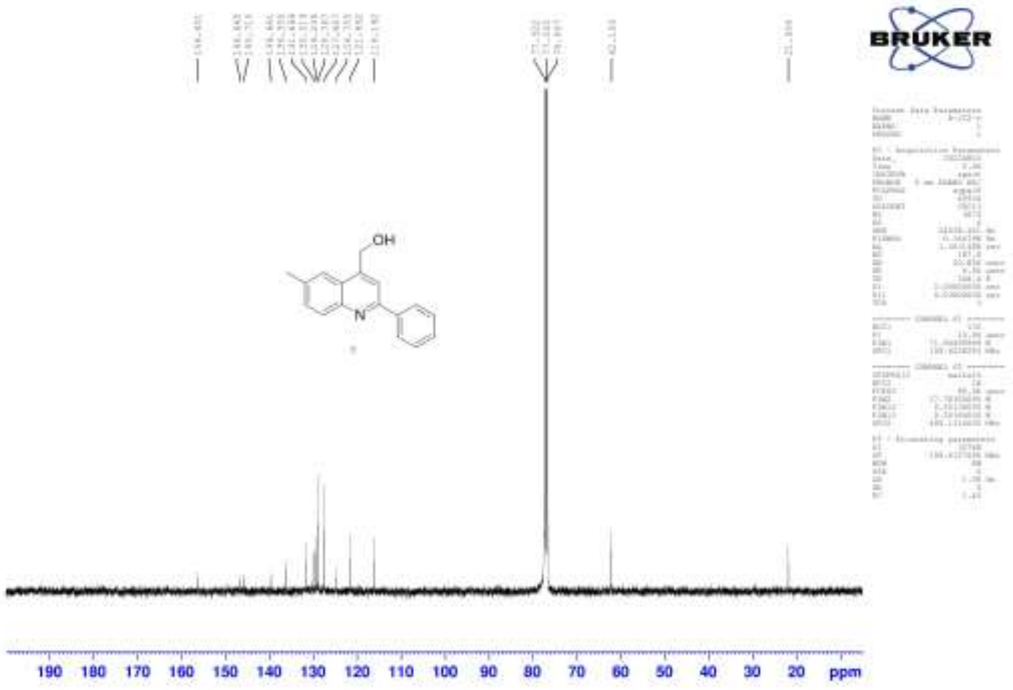
BRUKER
OH
N
190 180 170 160 150 140 130 120 110 100 90 80 70 60 50 40 30 20 ppm

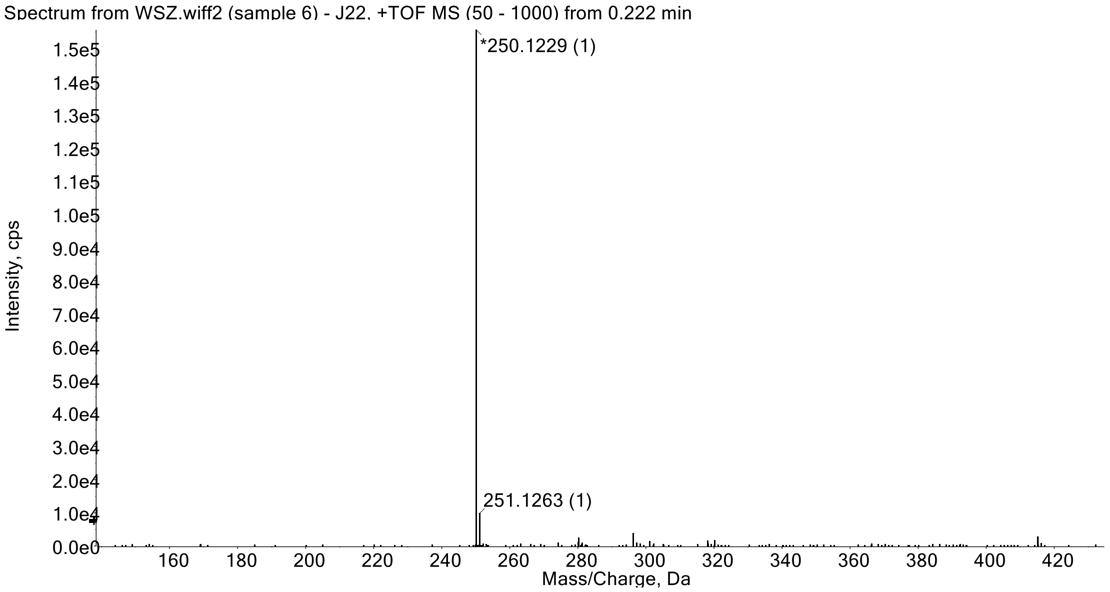
Spectrum from WSZ.wiff2 (sample 6) - J22, +TOF MS (50 - 1000) from 0.222 min
*250.1229 (1)
251.1263 (1)
Intensity, cps
1.5e5
1.4e5
1.3e5
1.2e5
1.1e5
1.0e5
9.0e4
8.0e4
7.0e4
6.0e4
5.0e4
4.0e4
3.0e4
2.0e4
1.0e4
0.0e0
160
180
200
220
240
260
280
300
320
340
360
380
400
420
Mass/Charge, Da

Compound **G8**

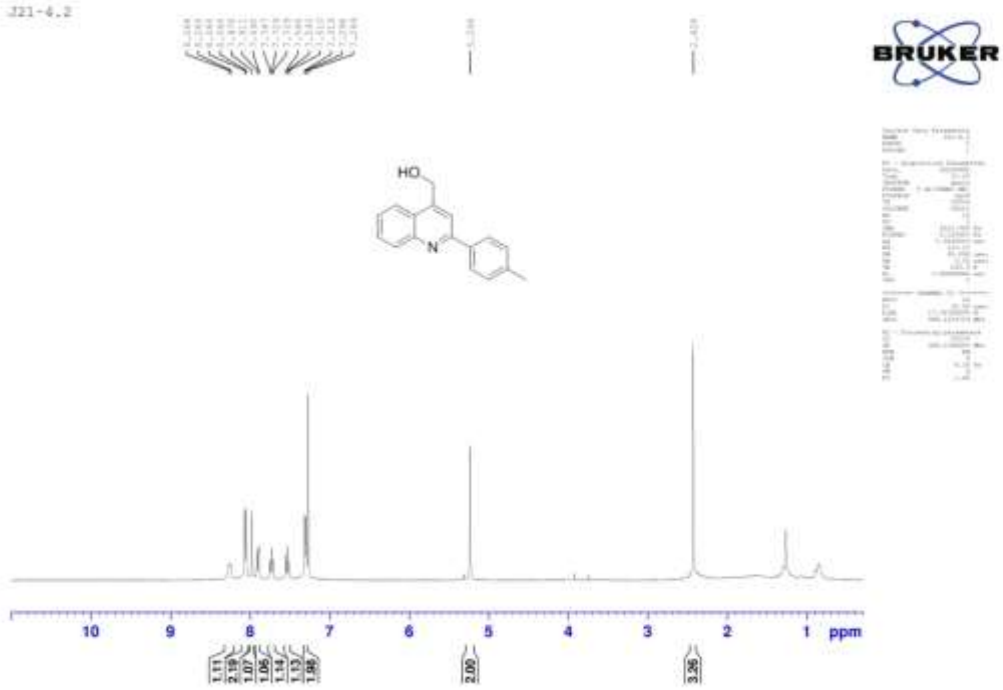

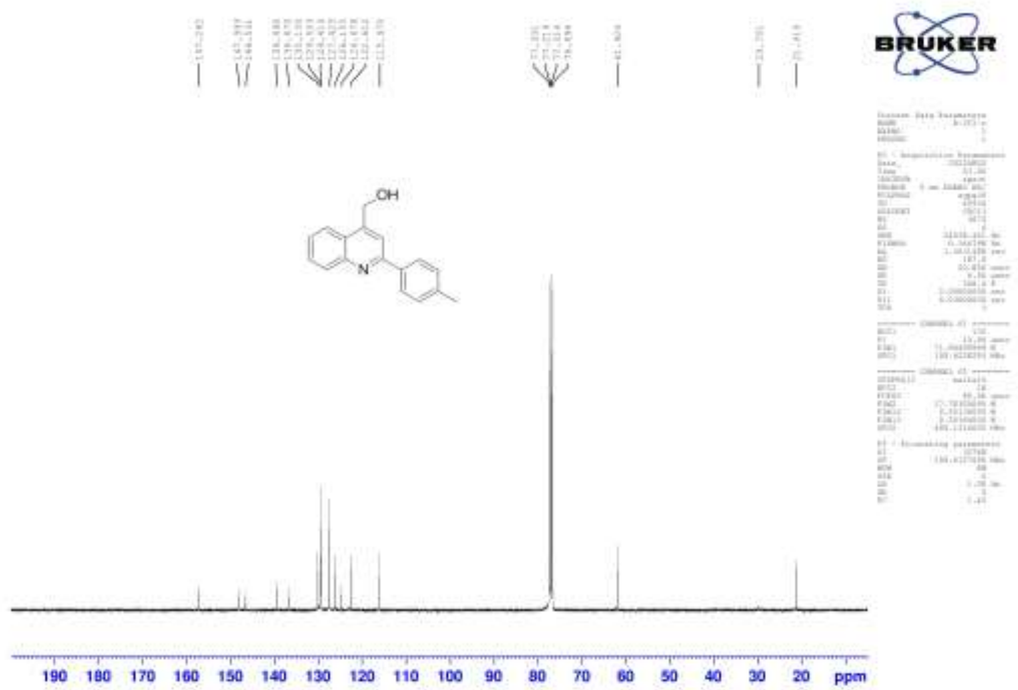

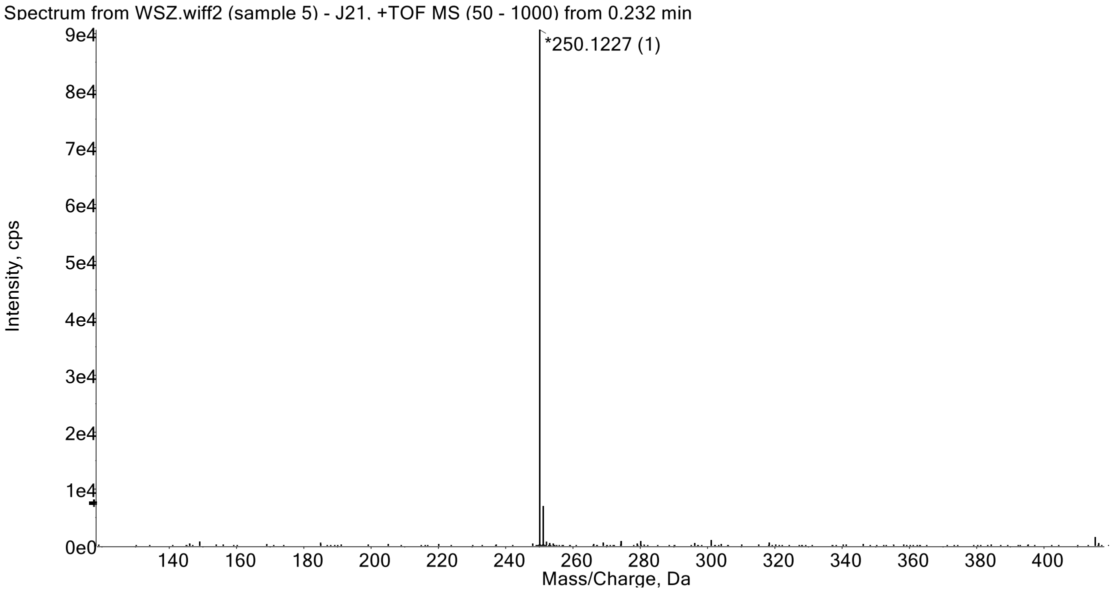
Spectrum from WSZ.wiff2 (sample 5) - J21, +TOF MS (50 - 1000) from 0.232 min
*250.1227 (1)
Intensity, cps
9e4
8e4
7e4
6e4
5e4
4e4
3e4
2e4
1e4
0e0
140
160
180
200
220
240
260
280
300
320
340
360
380
400
Mass/Charge, Da

Compound **G9**

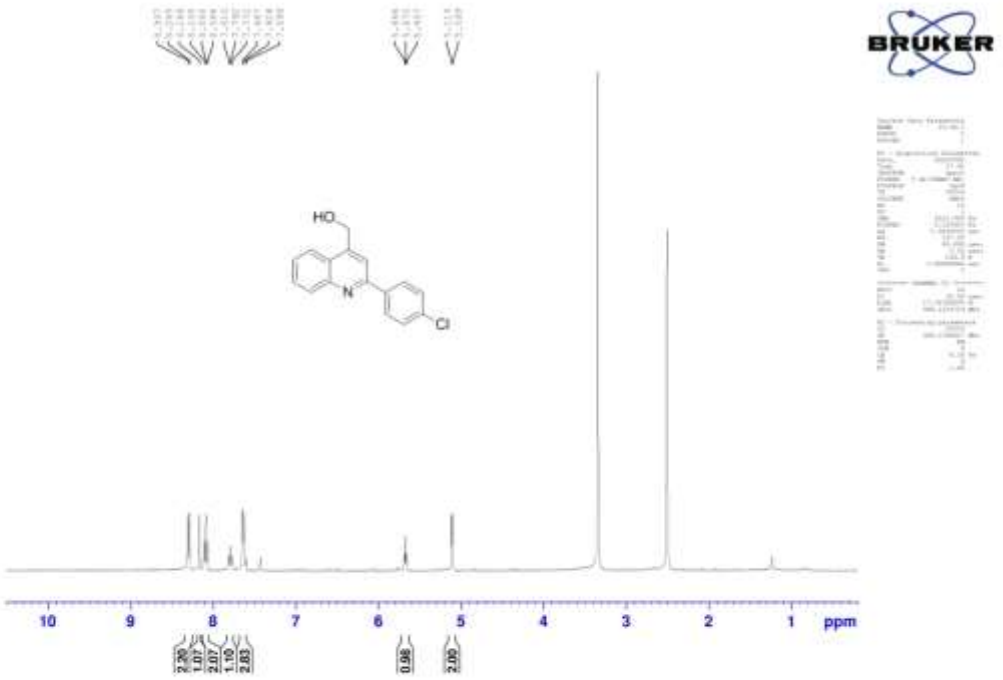
BRUKER
HO
N
Cl
10 9 8 7 6 5 4 3 2 1 ppm
2.20
1.07
2.07
1.10
2.83
0.98
2.00

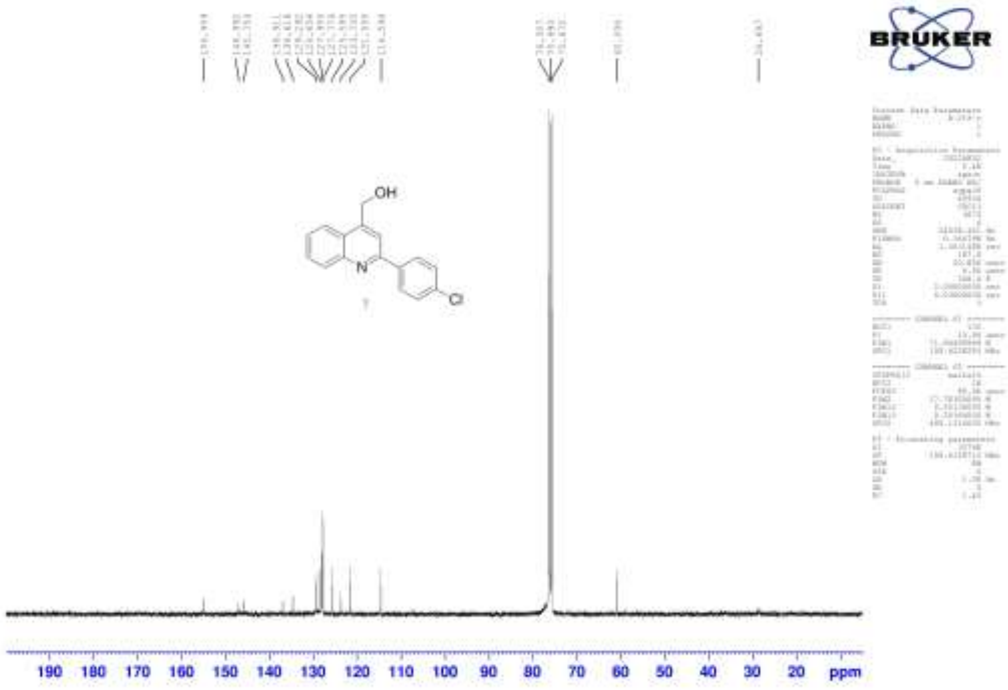
BRUKER
OH
N
Cl
190 180 170 160 150 140 130 120 110 100 90 80 70 60 50 40 30 20 ppm

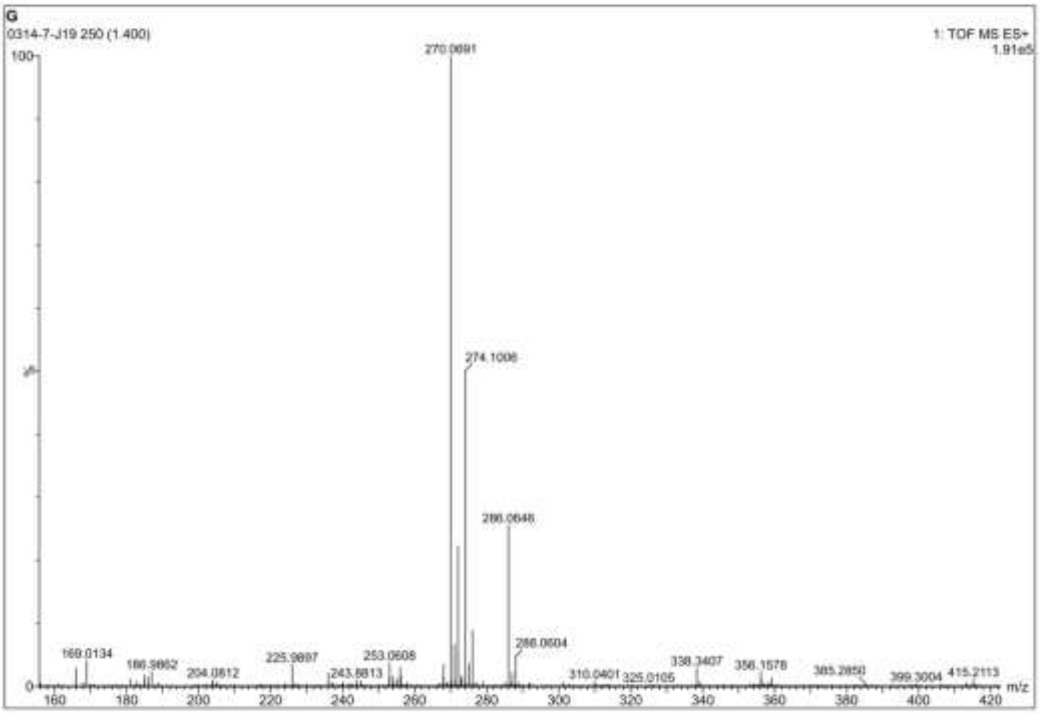
G
0314-7-J19 250 (1.400)
1: TOF MS ES+
1.91e5
100
%
0
270.0691
274.1006
286.0646
288.0604
169.0134
186.9862
204.0812
225.9897
243.8813
253.0608
310.0401
325.0105
338.3407
356.1578
385.2850
399.3004
415.2113
160
180
200
220
240
260
280
300
320
340
360
380
400
420
m/z

Compound **G10**

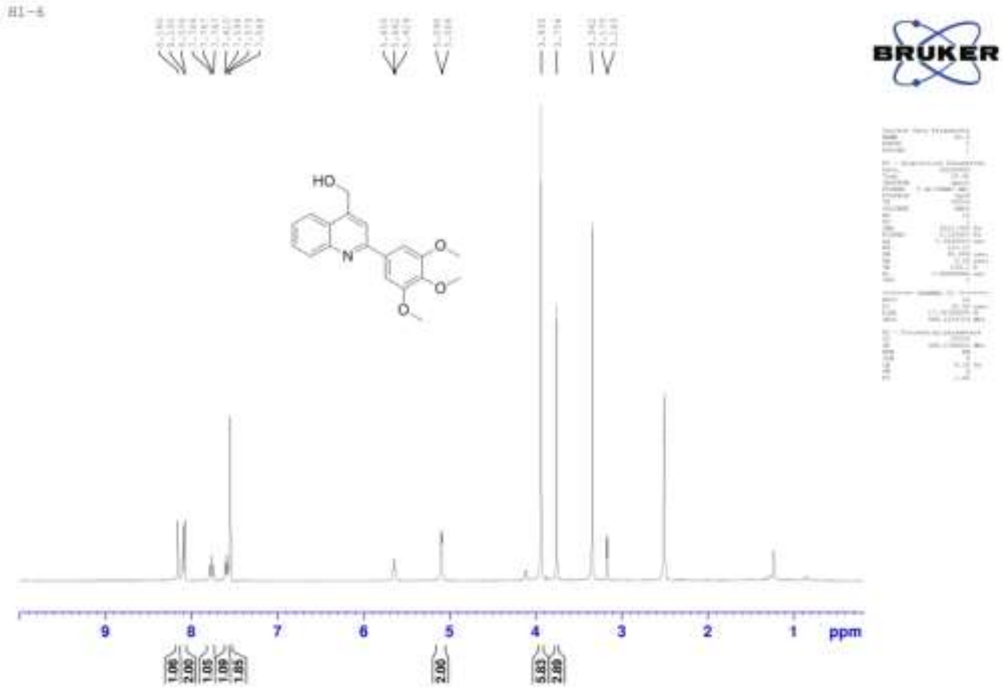

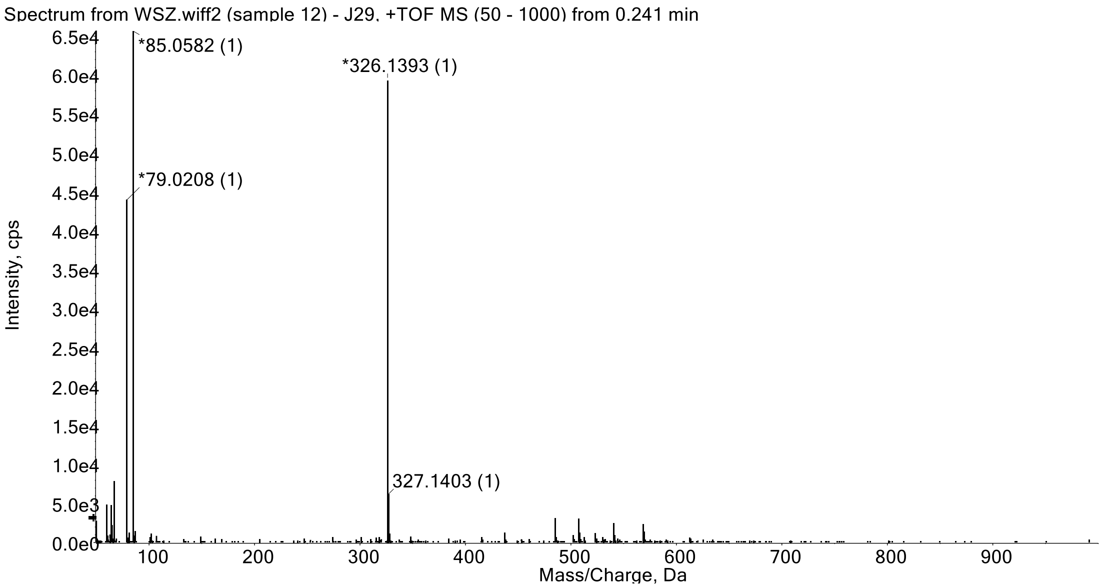

Compound **G11**

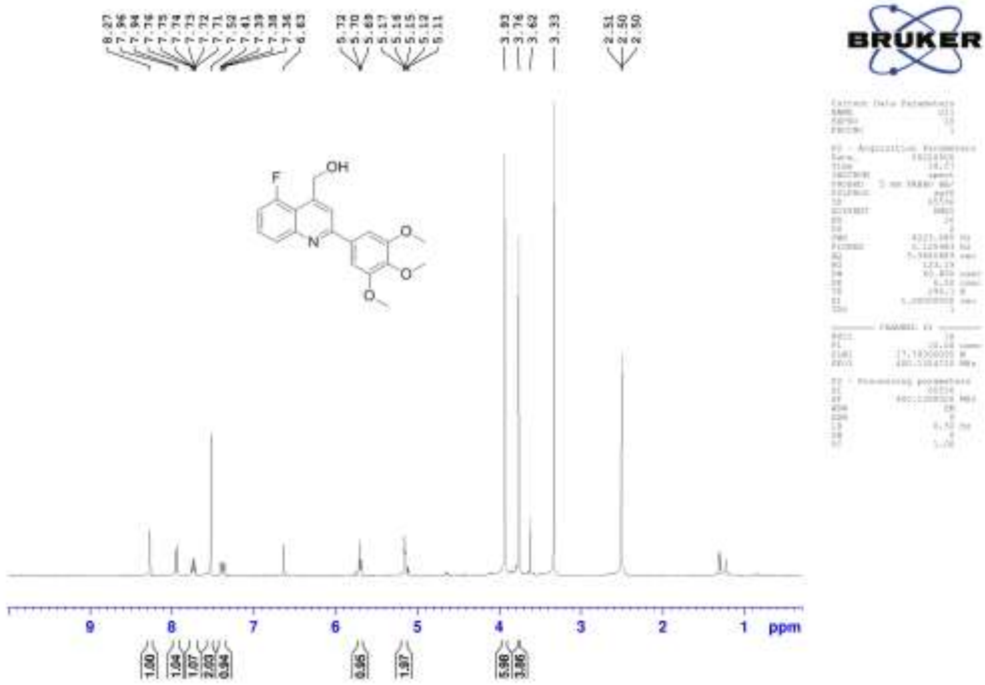

Spectrum from WSZ.wiff2 (sample 18) - J36, +TOF MS (50 - 1000) from 0.259 min

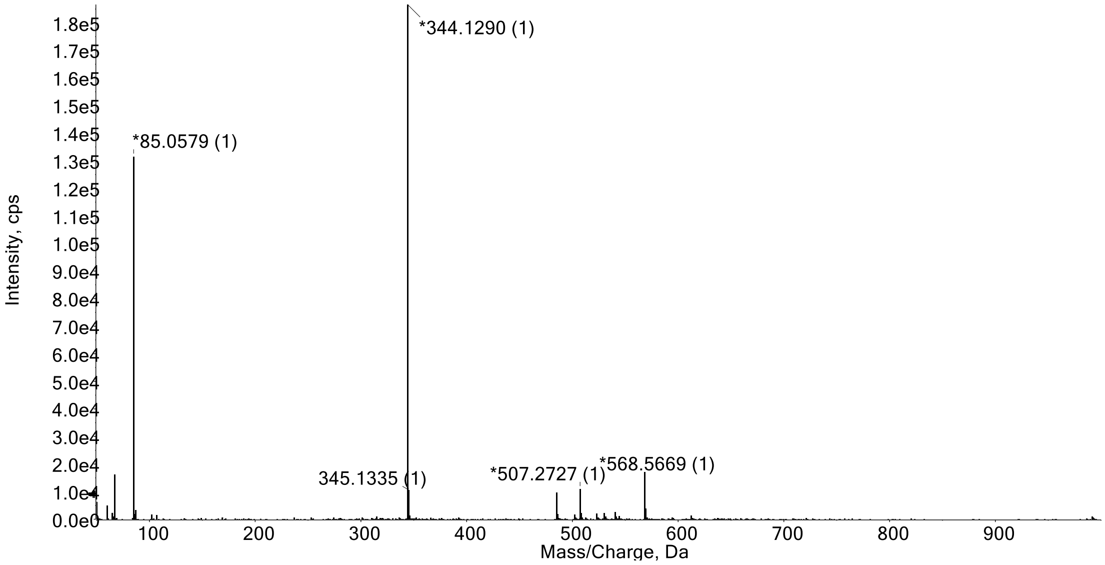

Compound **G12**

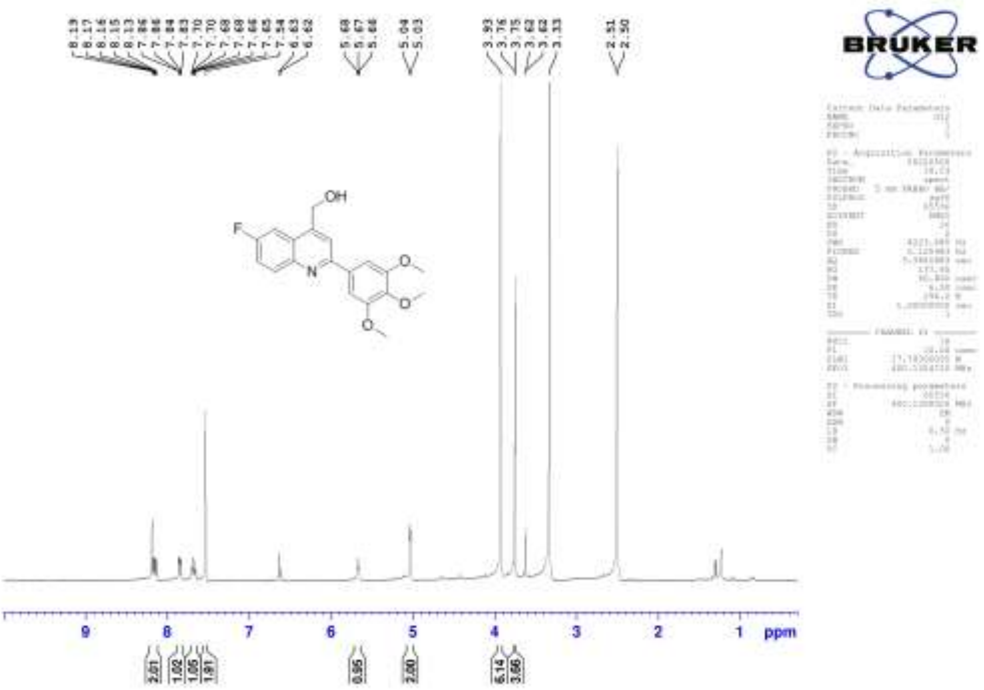

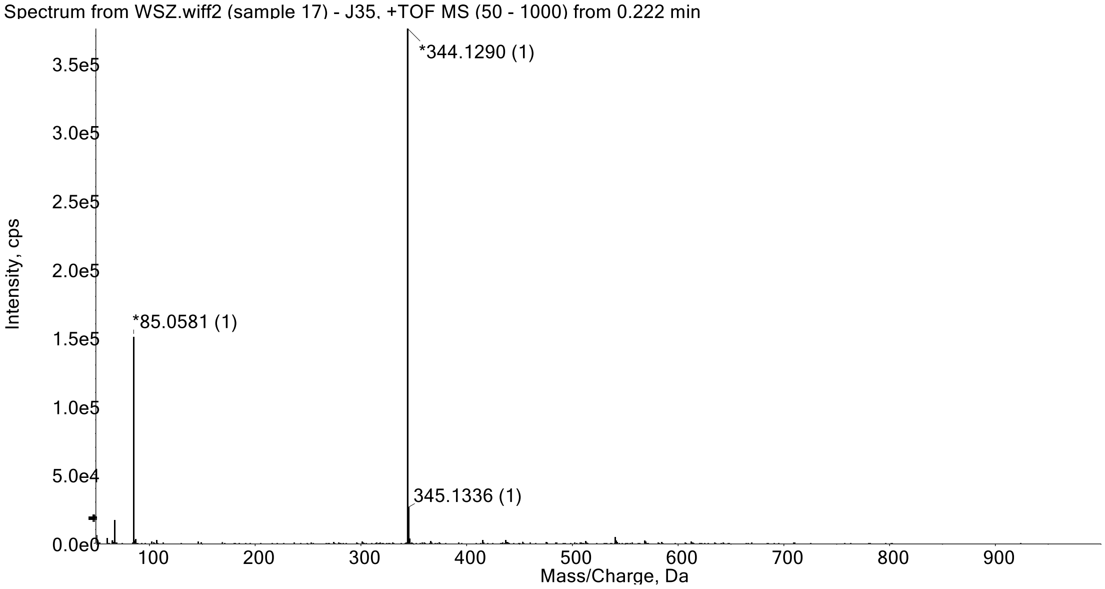

Compound **G13**

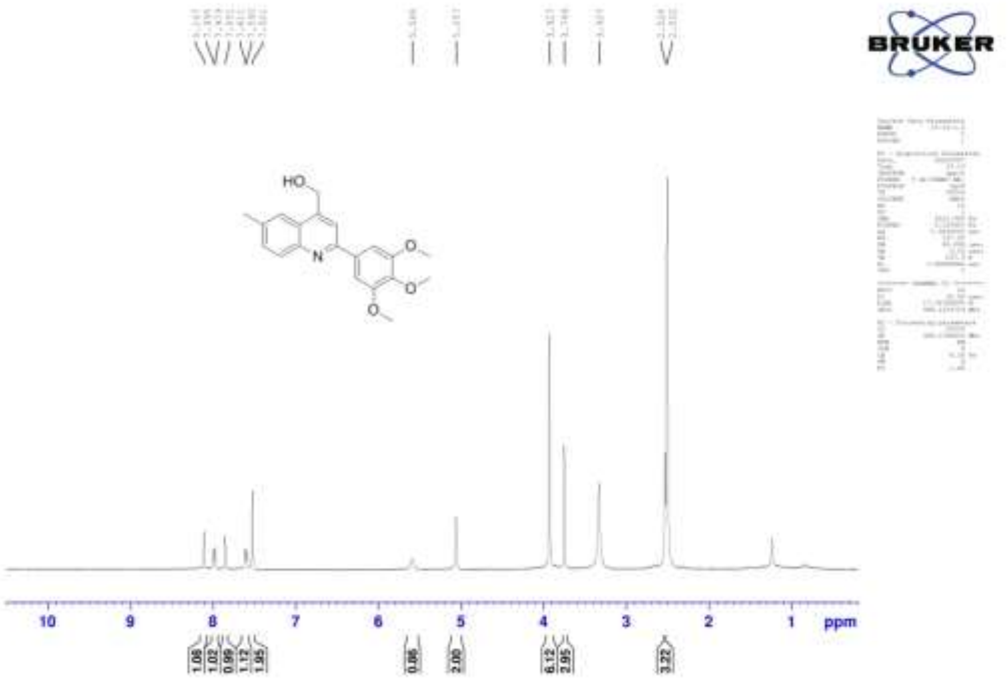

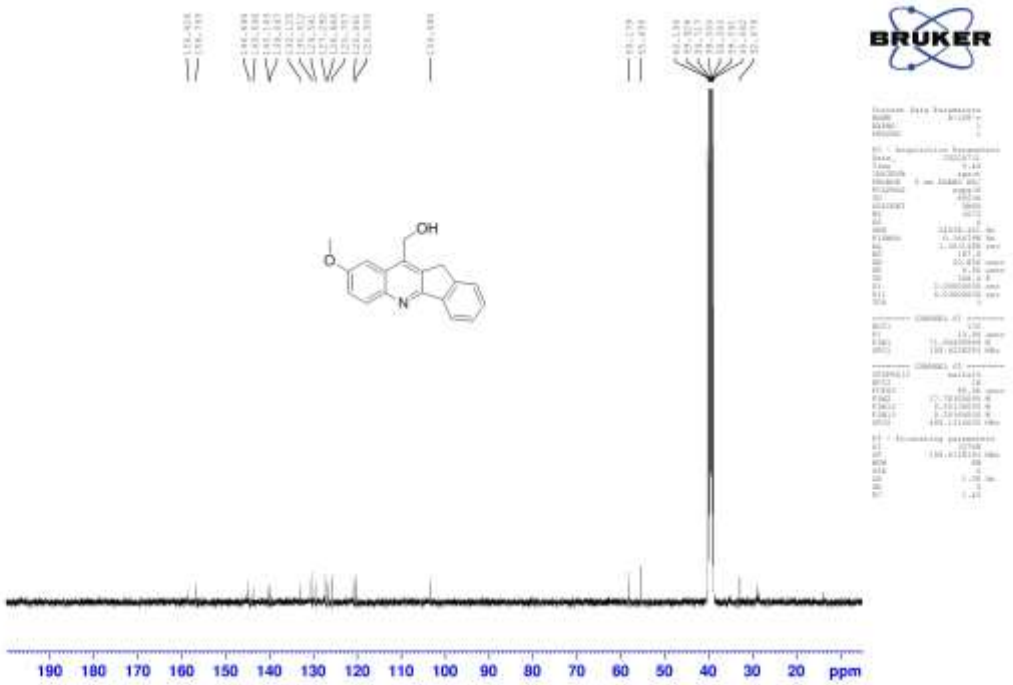

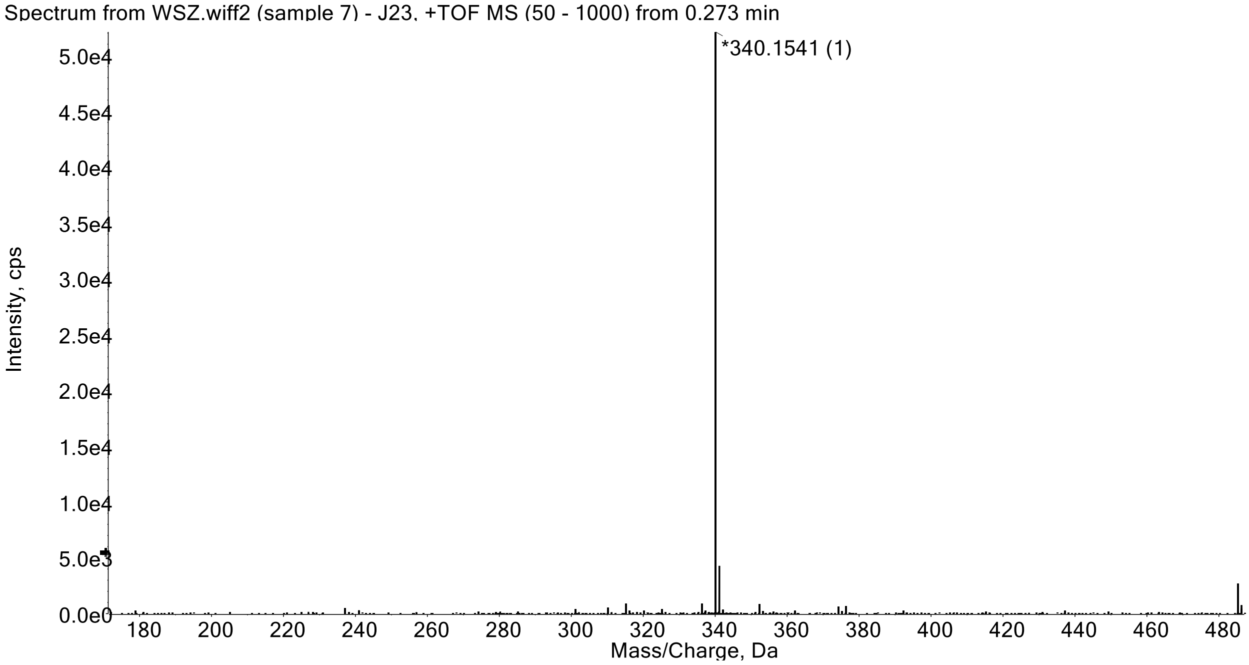
Spectrum from WSZ.wiff2 (sample 7) - J23, +TOF MS (50 - 1000) from 0.273 min
*340.1541 (1)
Intensity, cps
5.0e4
4.5e4
4.0e4
3.5e4
3.0e4
2.5e4
2.0e4
1.5e4
1.0e4
5.0e3
0.0e0
180
200
220
240
260
280
300
320
340
360
380
400
420
440
460
480
Mass/Charge, Da

Compound **G14**

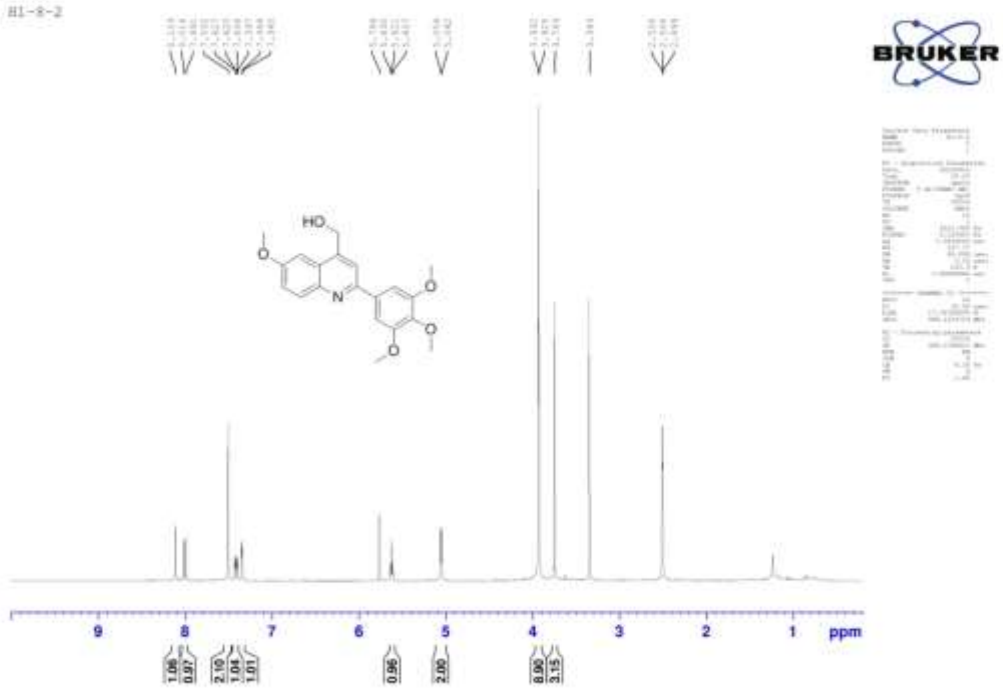

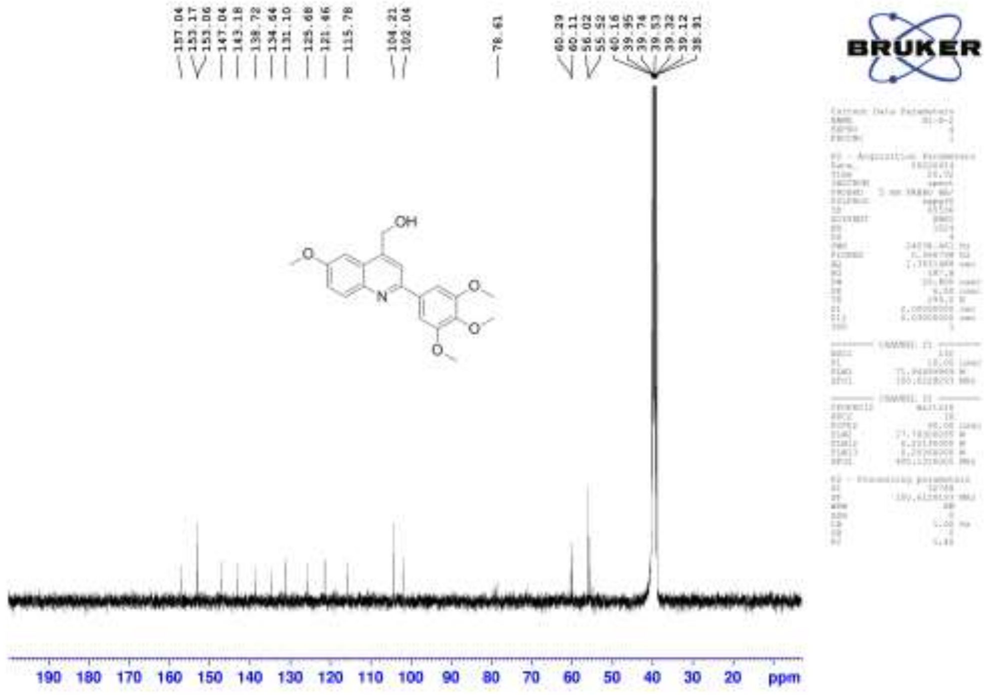

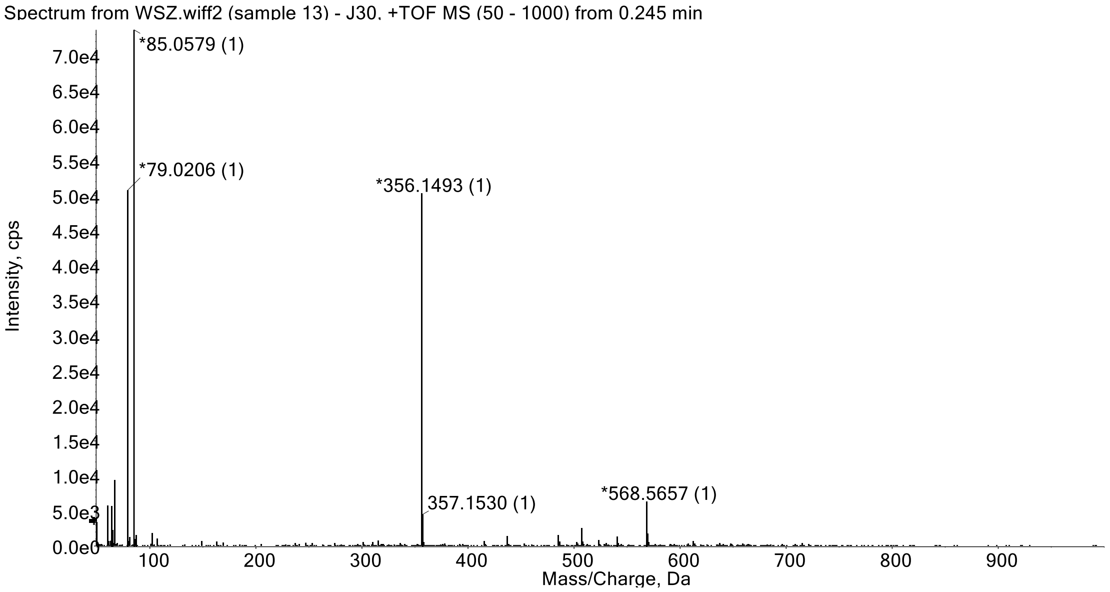

Spectrum from WSZ.wiff2 (sample 13) - J30, +TOF MS (50 - 1000) from 0.245 min
*85.0579 (1)
*79.0206 (1)
*356.1493 (1)
357.1530 (1)
*568.5657 (1)
Intensity, cps
Mass/Charge, Da

Compound **G15**

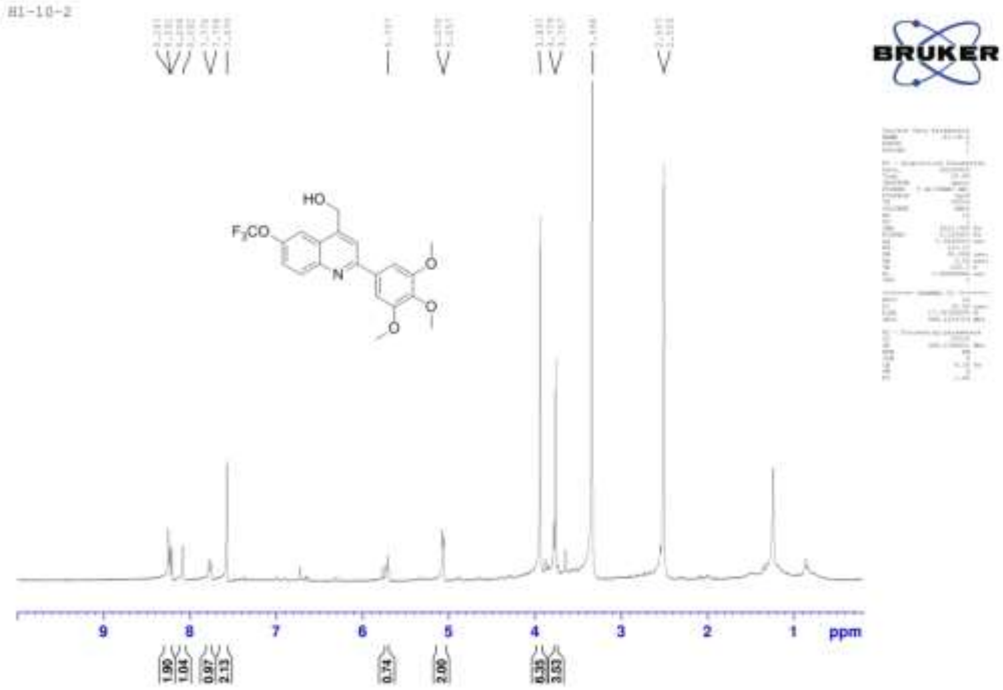

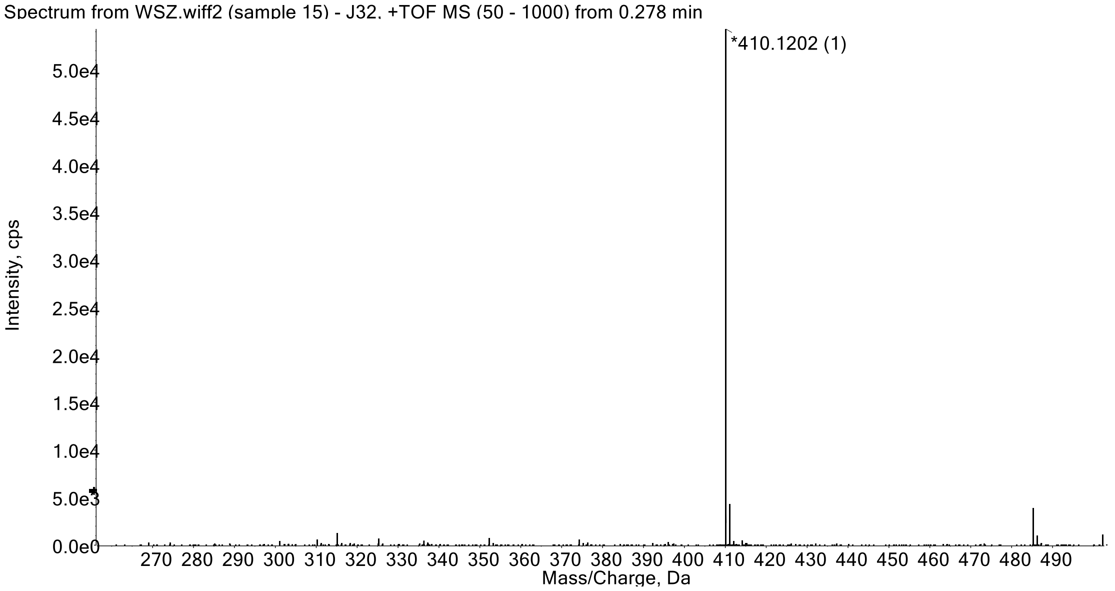

Compound **G16**

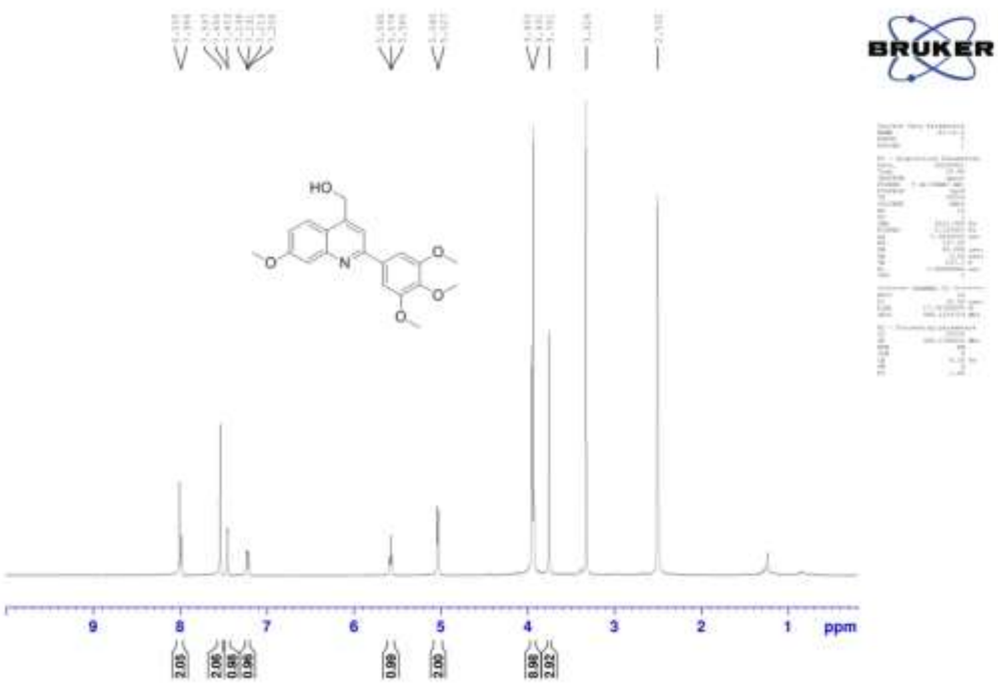

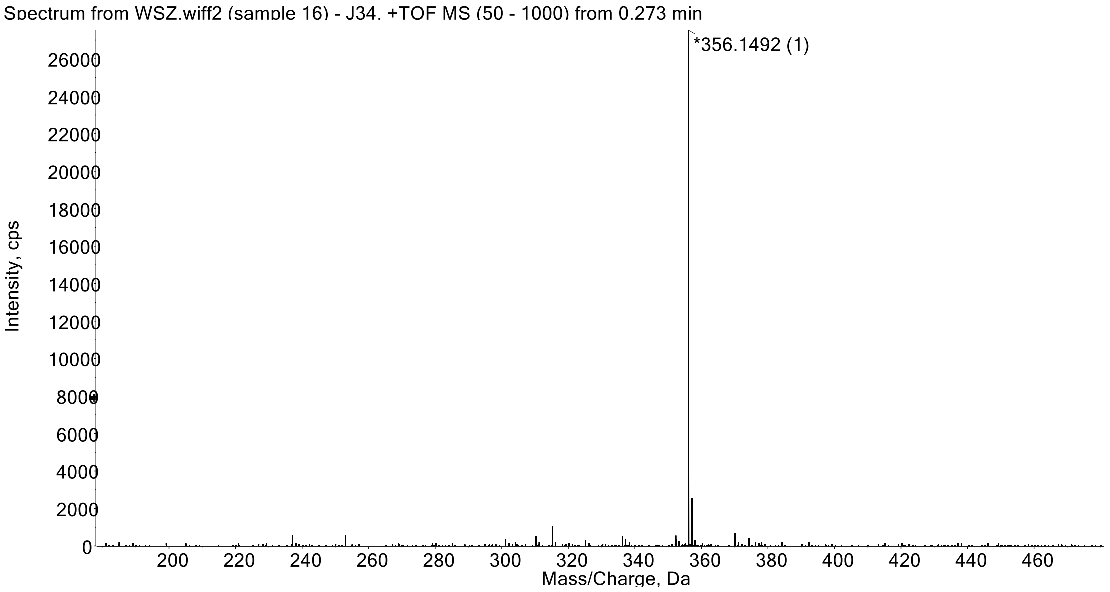

Compound **G17**

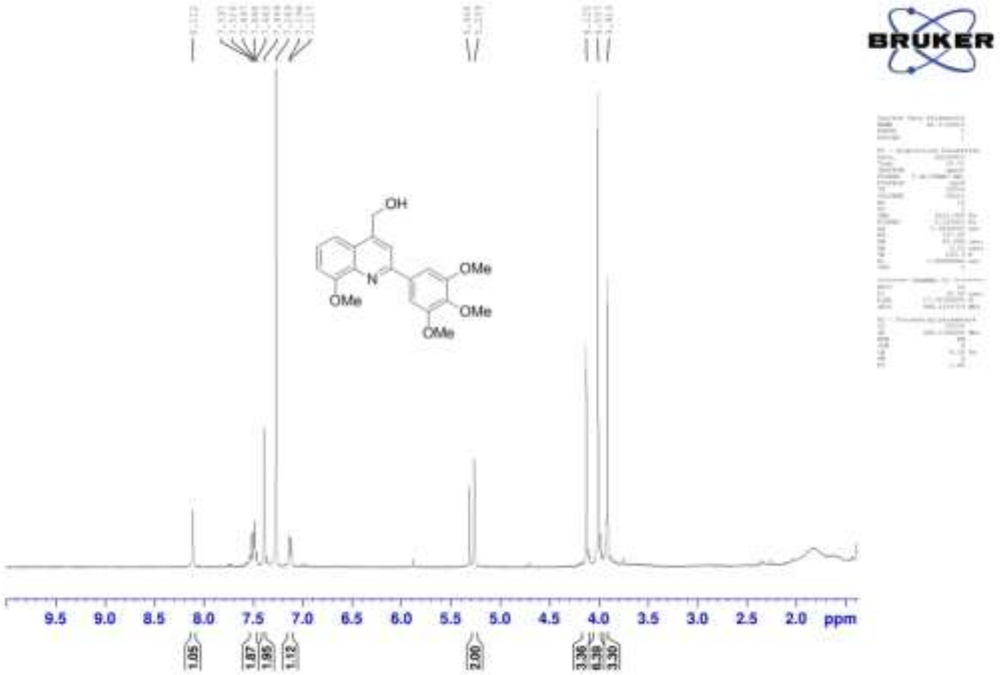

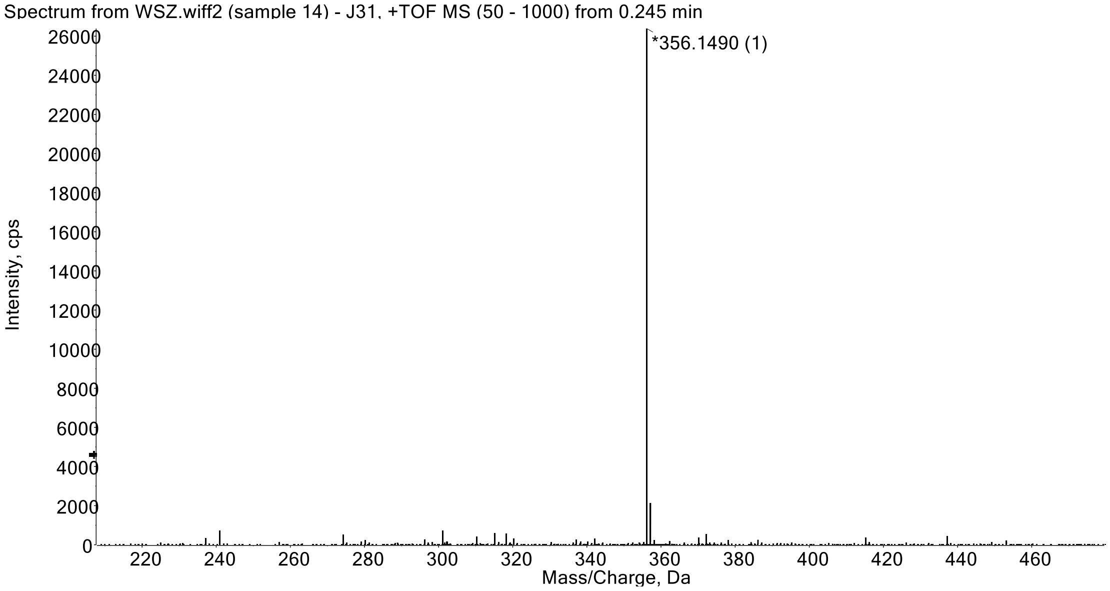

Compound **G18**

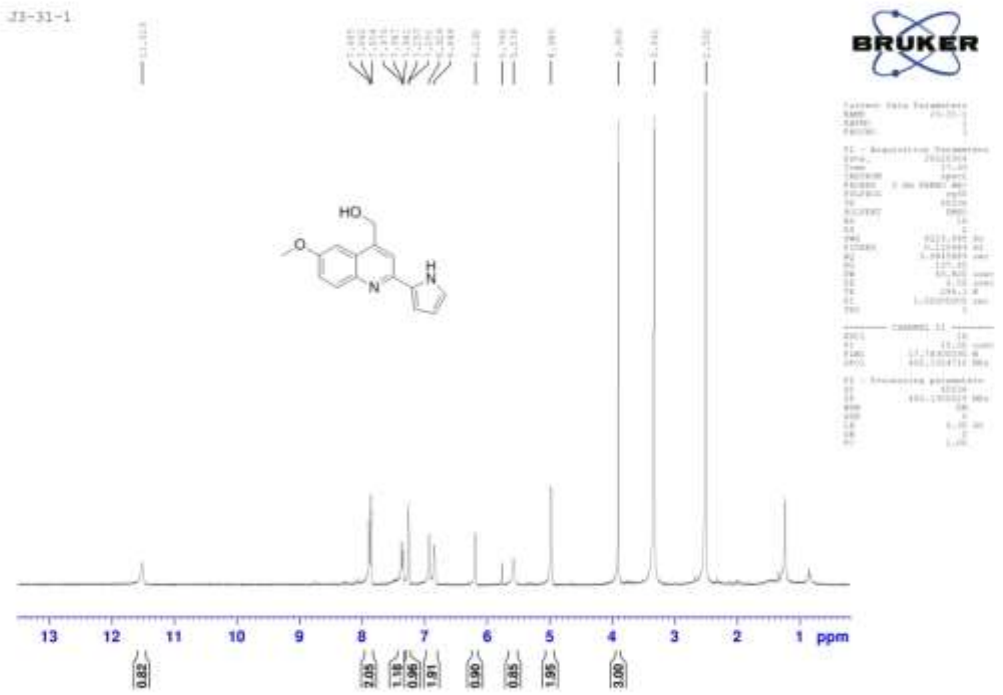

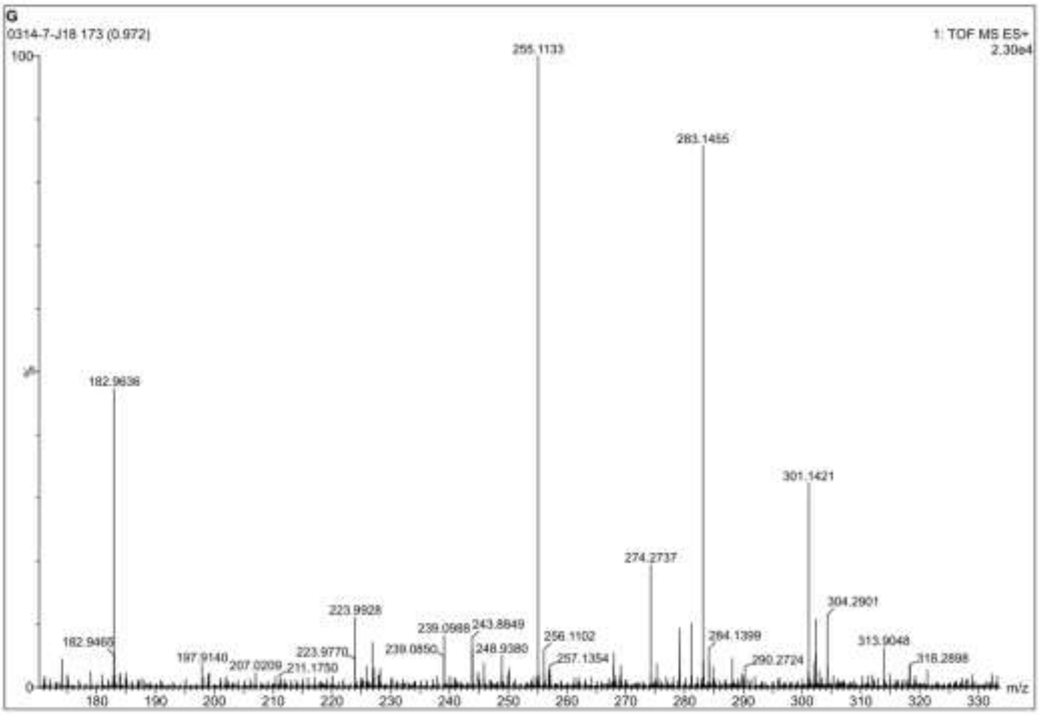

Compound **G19**

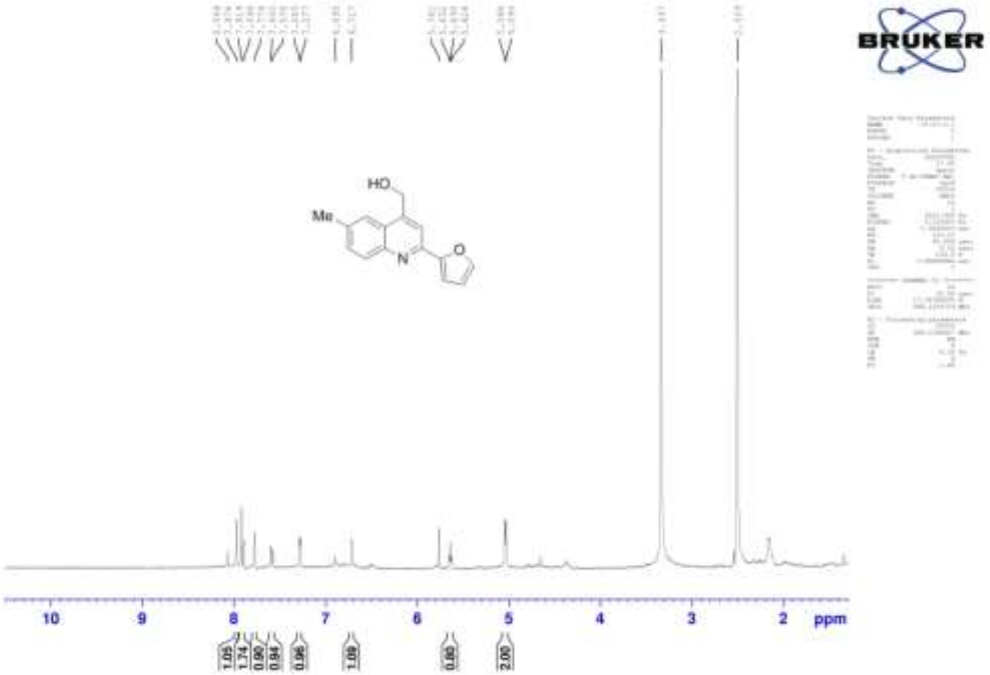

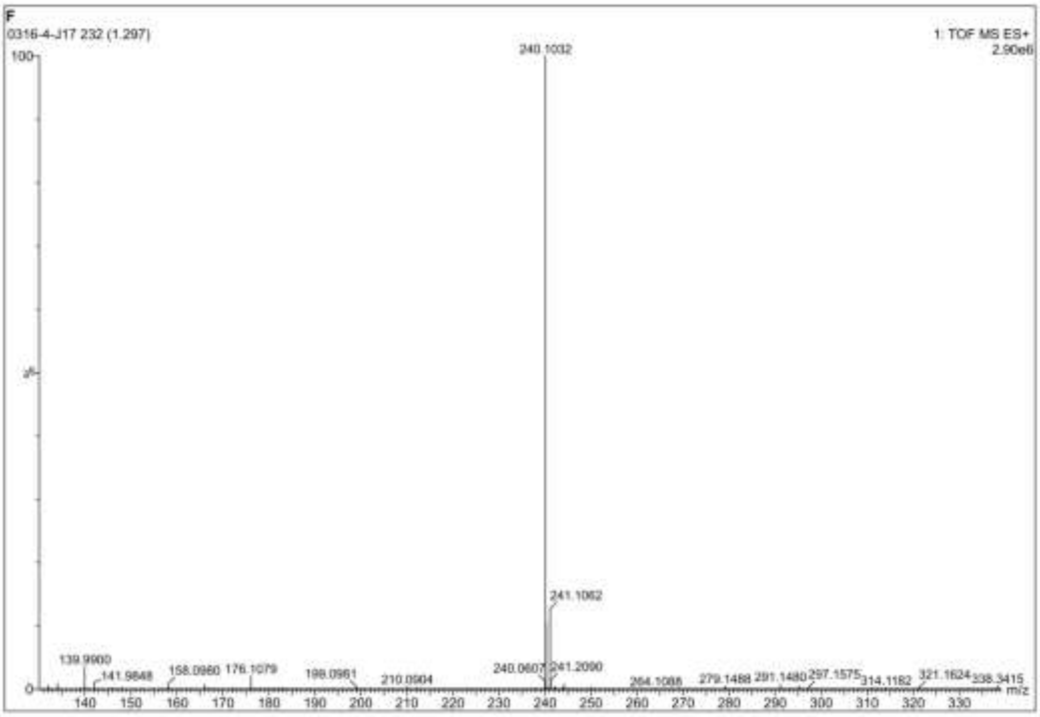

Compound **G20**

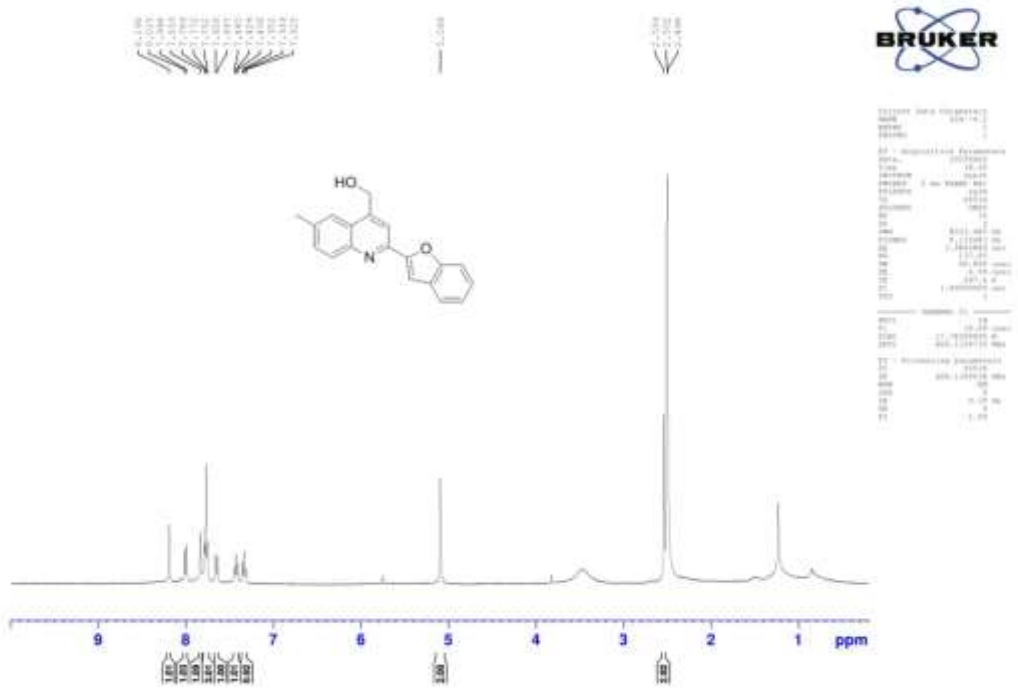

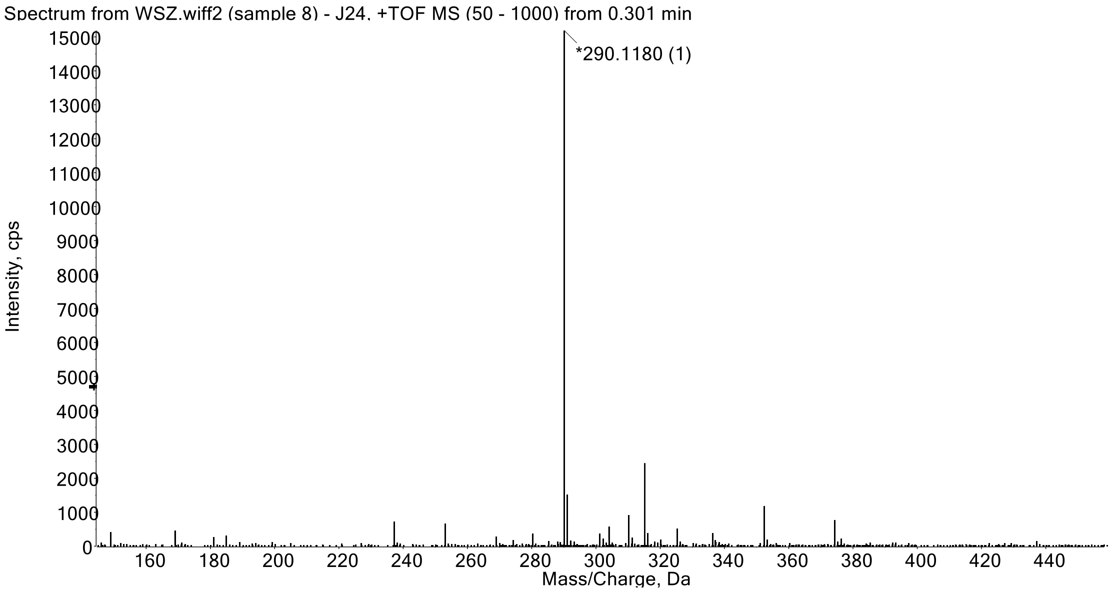

Compound **G21**

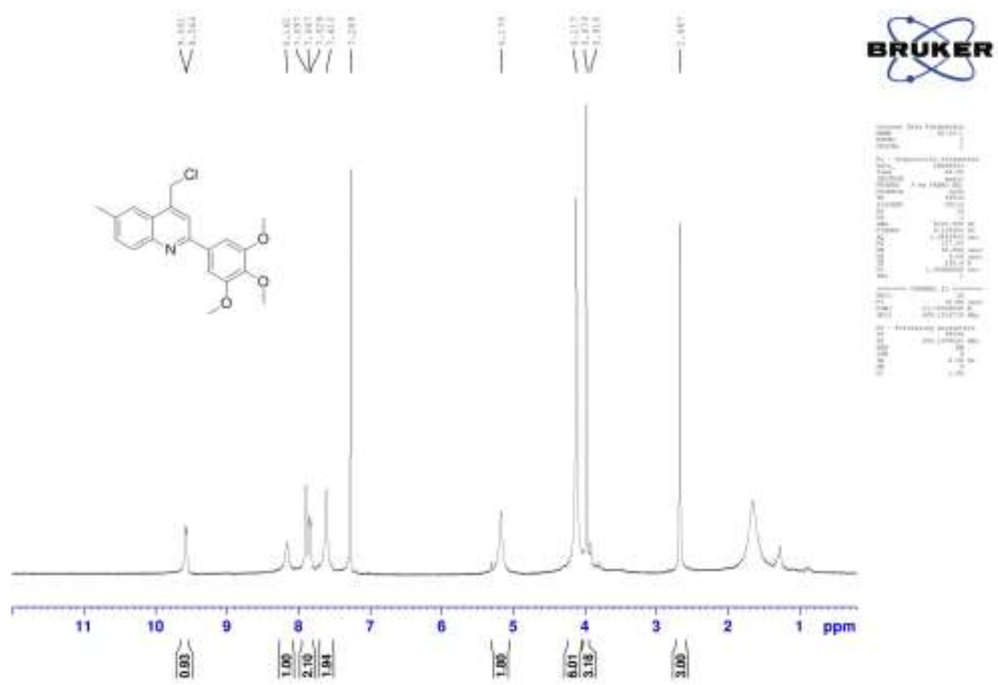

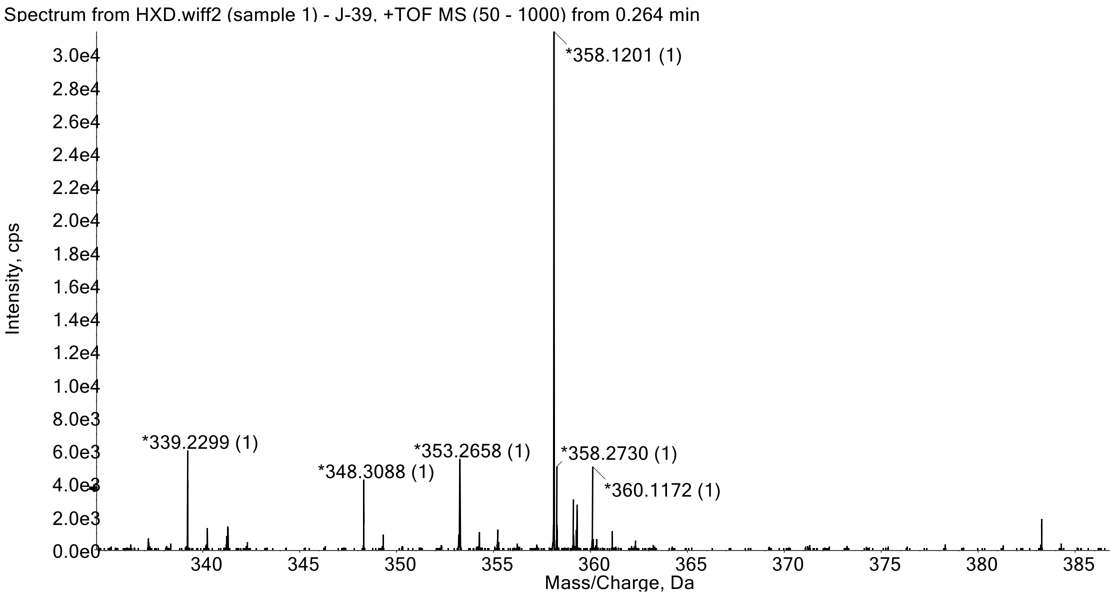

Compound **G22**

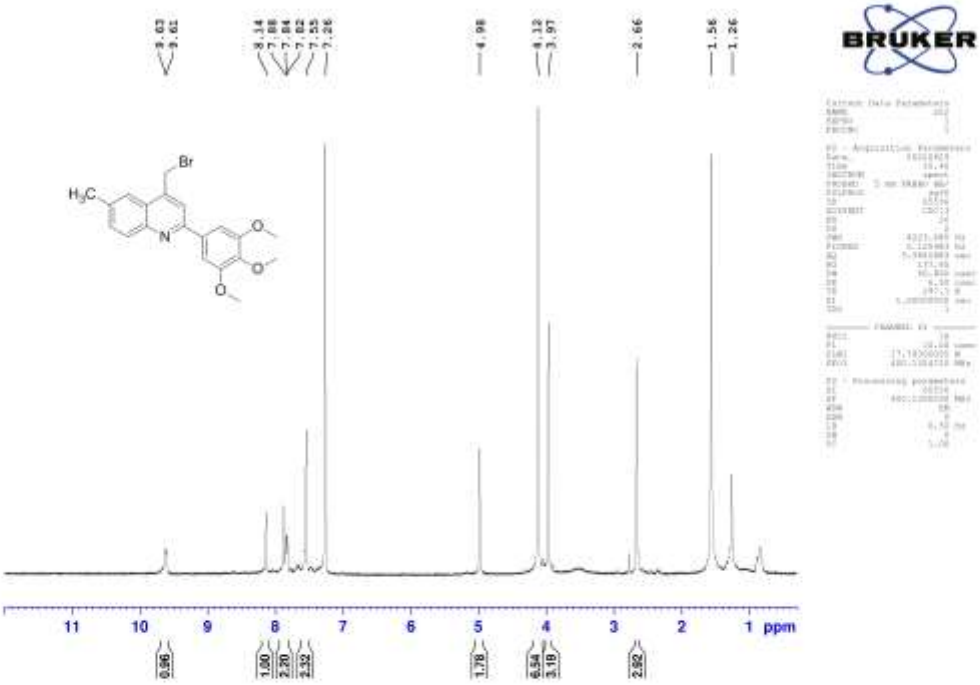

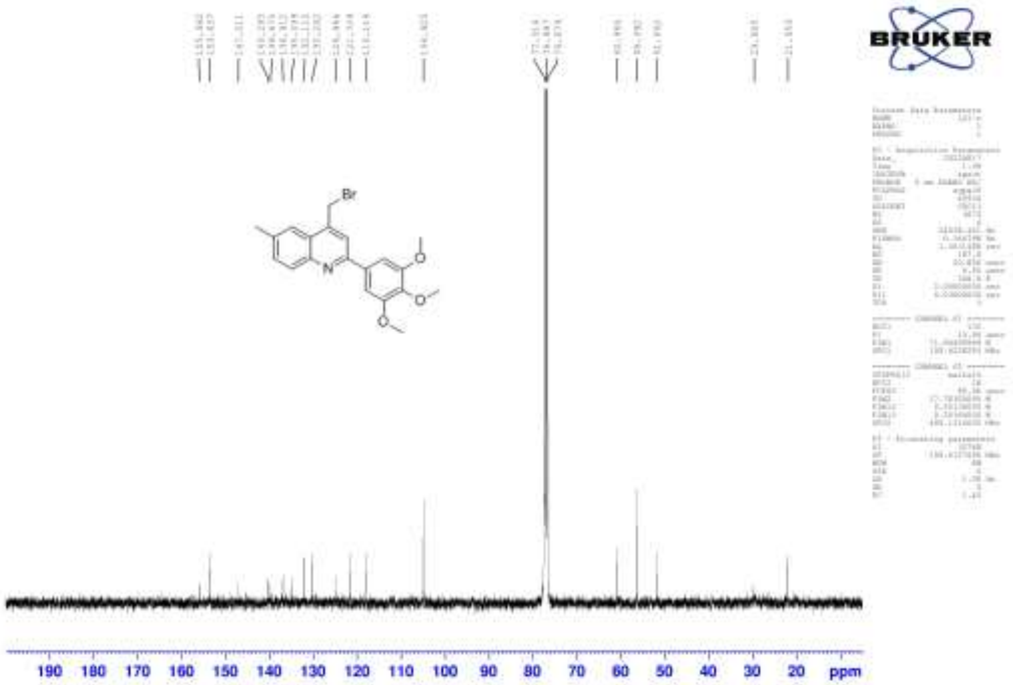

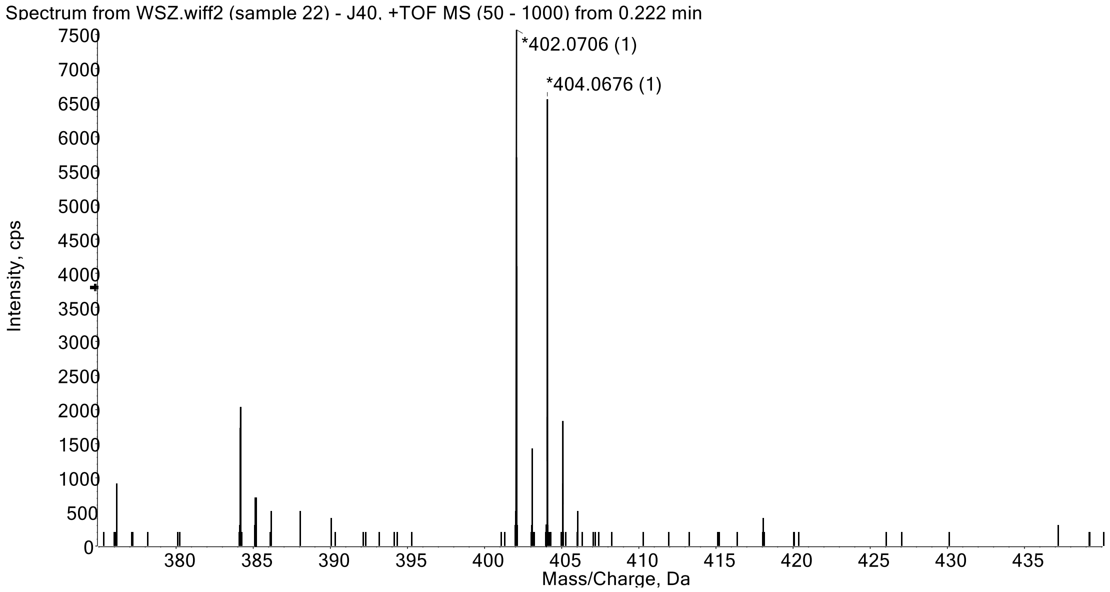
Spectrum from WSZ.wiff2 (sample 22) - J40, +TOF MS (50 - 1000) from 0.222 min
*402.0706 (1)
*404.0676 (1)
Intensity, cps
Mass/Charge, Da

Compound **G23**

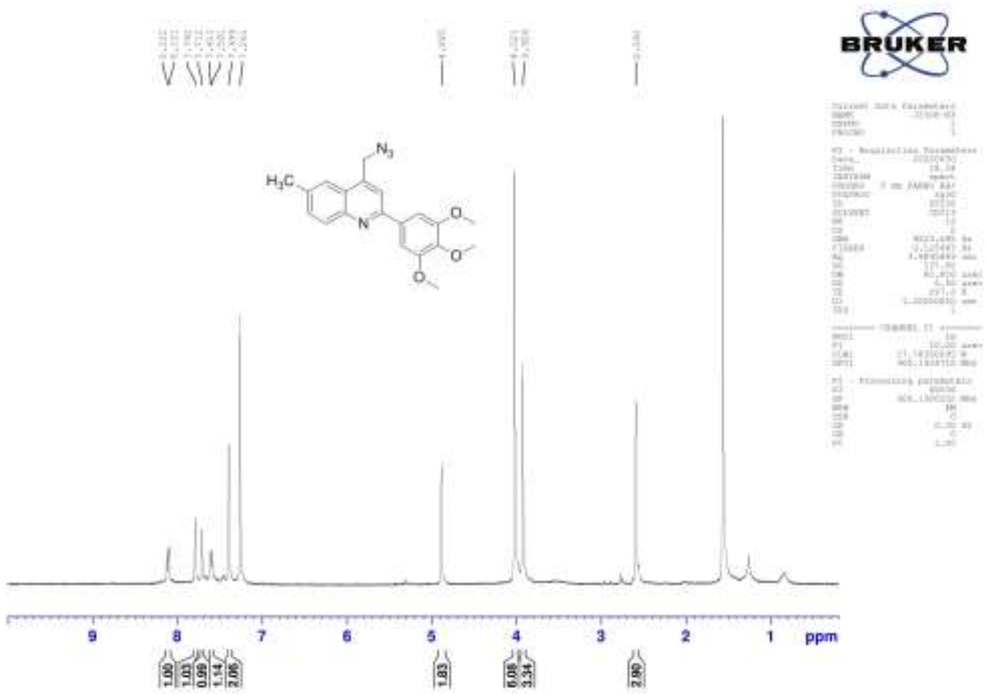

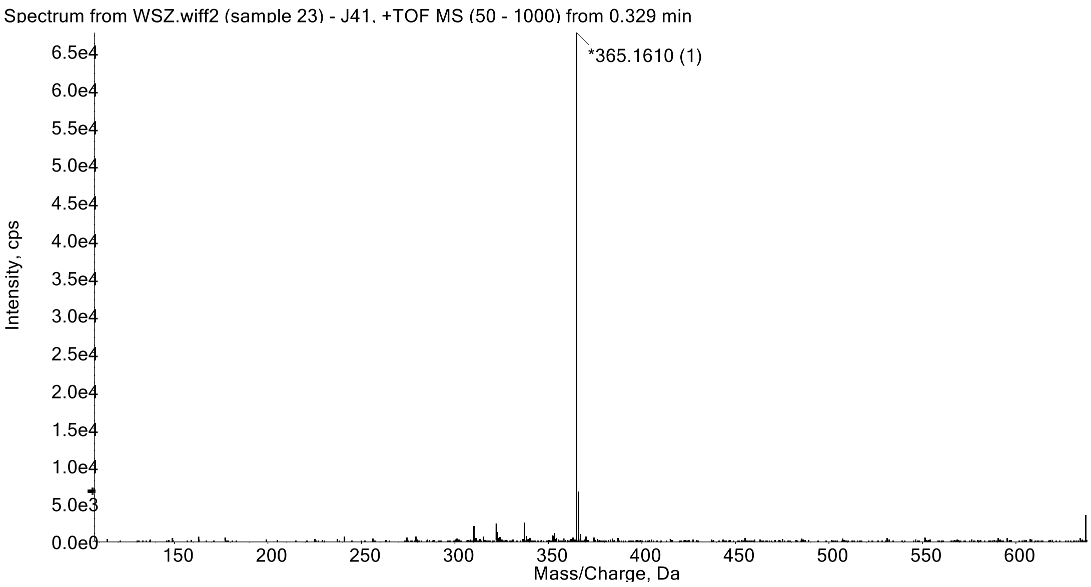

Compound **G24**

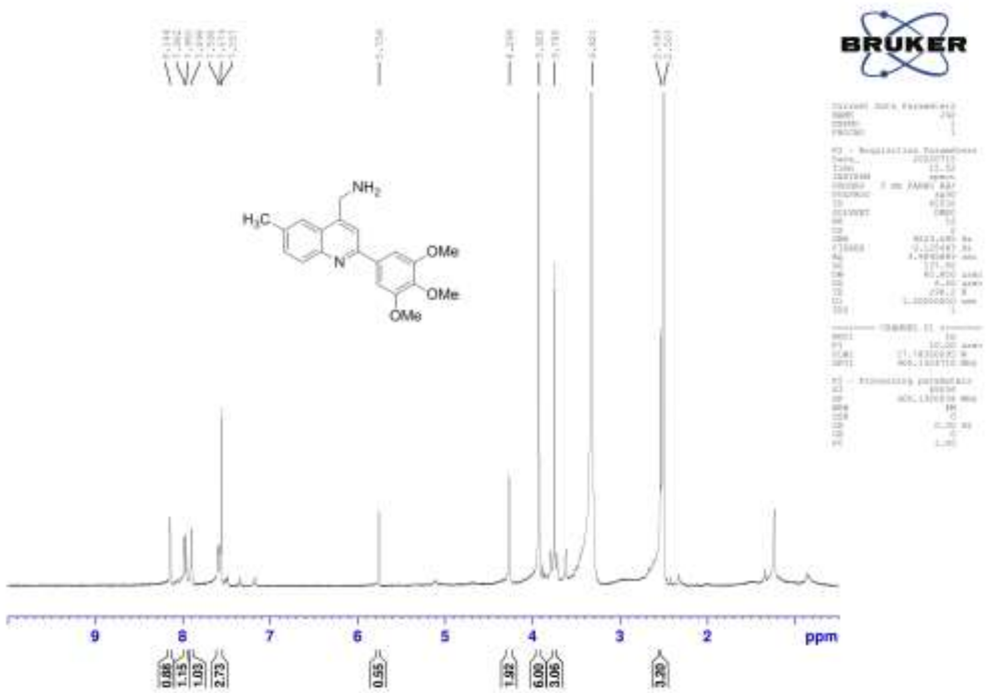

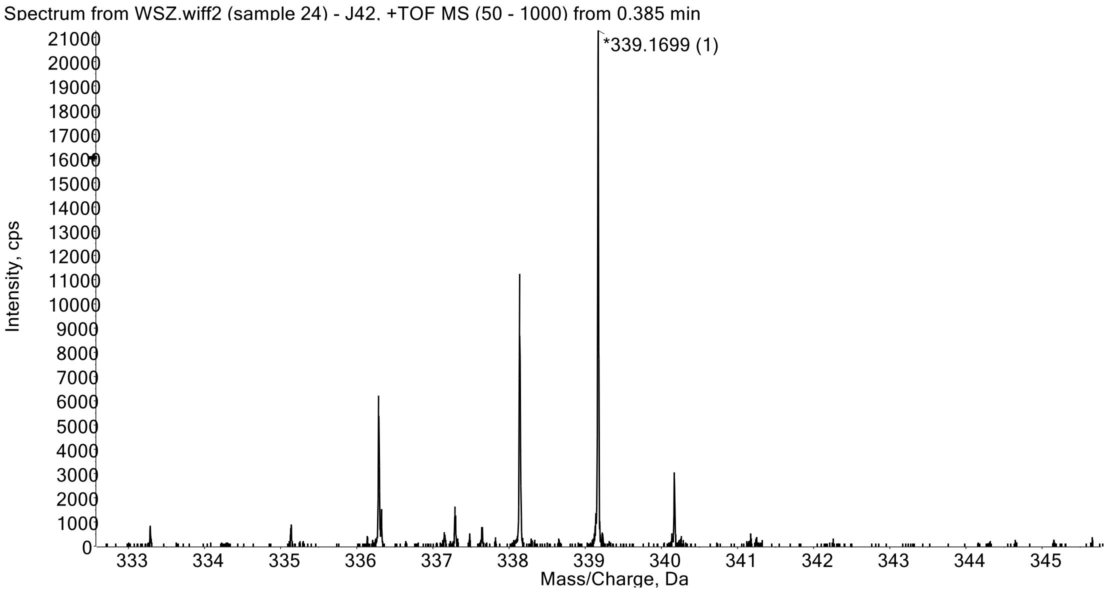

Compound **I1**

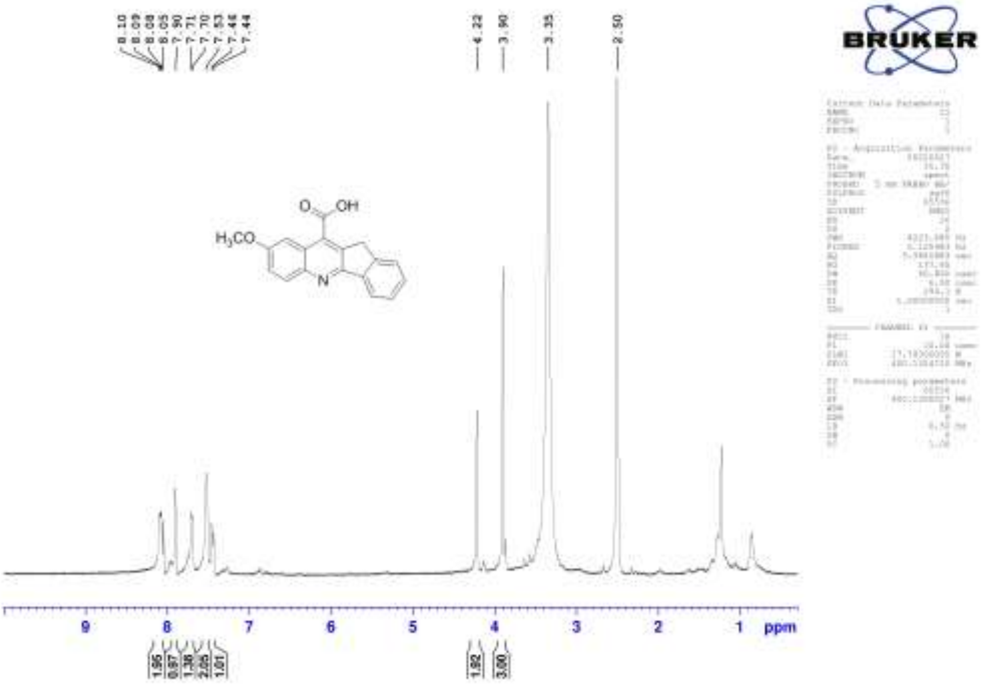

8.10
8.09
8.08
8.05
7.90
7.71
7.70
7.53
7.48
7.44
4.22
3.90
3.35
2.50
H3CO
OH
N
BRUKER
ppm
1.95
0.97
1.38
2.05
1.01
1.92
3.00

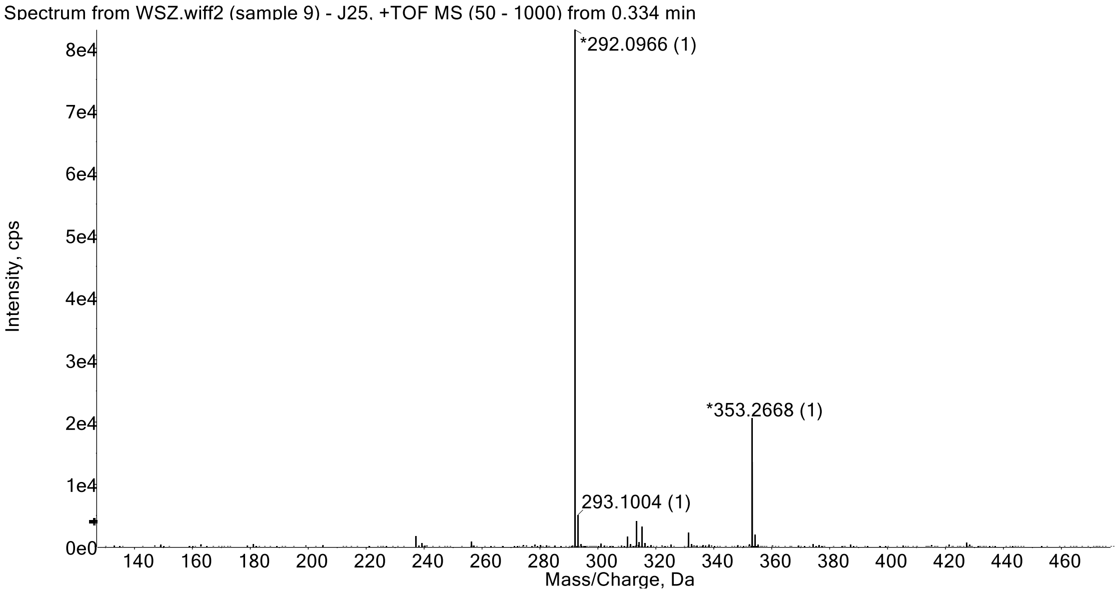

Spectrum from WSZ.wiff2 (sample 9) - J25, +TOF MS (50 - 1000) from 0.334 min
*292.0966 (1)
*353.2668 (1)
293.1004 (1)
Intensity, cps
Mass/Charge, Da

Compound **I2**

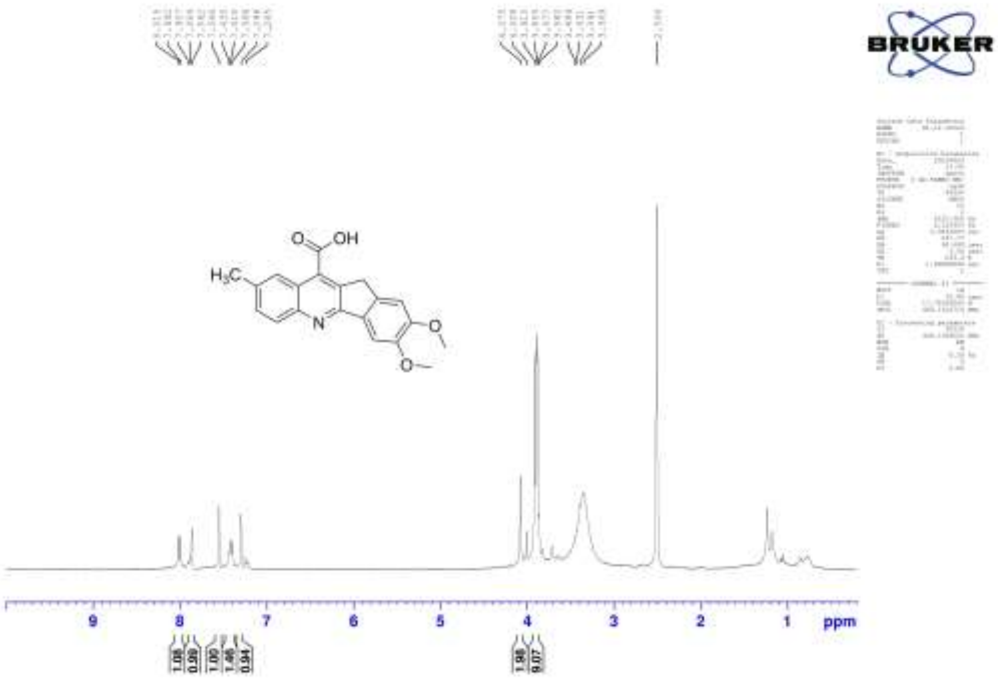

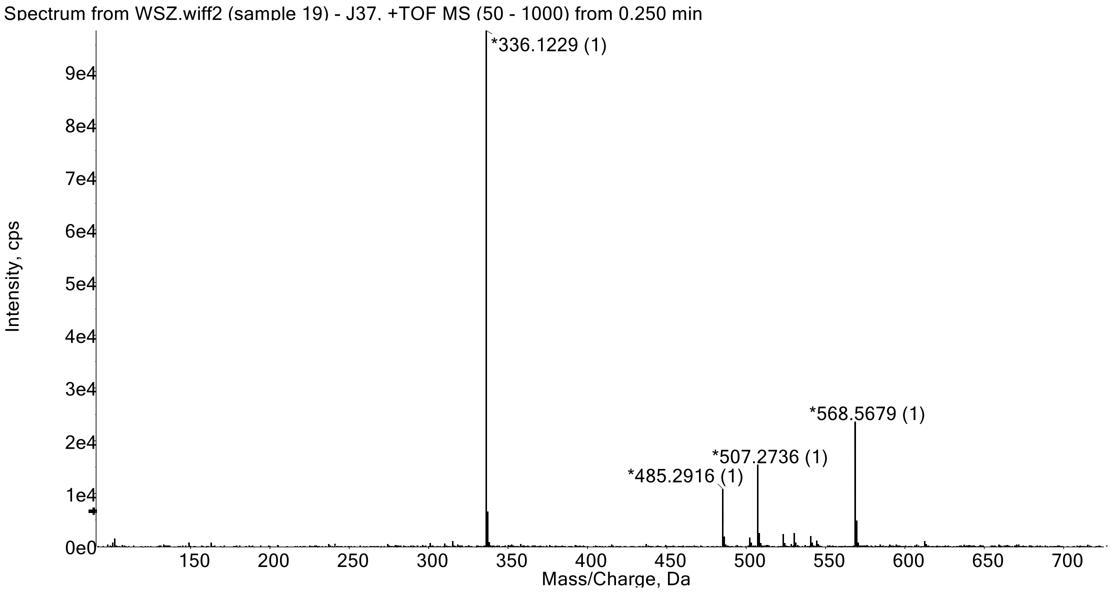

Spectrum from WSZ.wiff2 (sample 19) - J37, +TOF MS (50 - 1000) from 0.250 min
*336.1229 (1)
*568.5679 (1)
*507.2736 (1)
*485.2916 (1)
Intensity, cps
Mass/Charge, Da

Compound **J1**

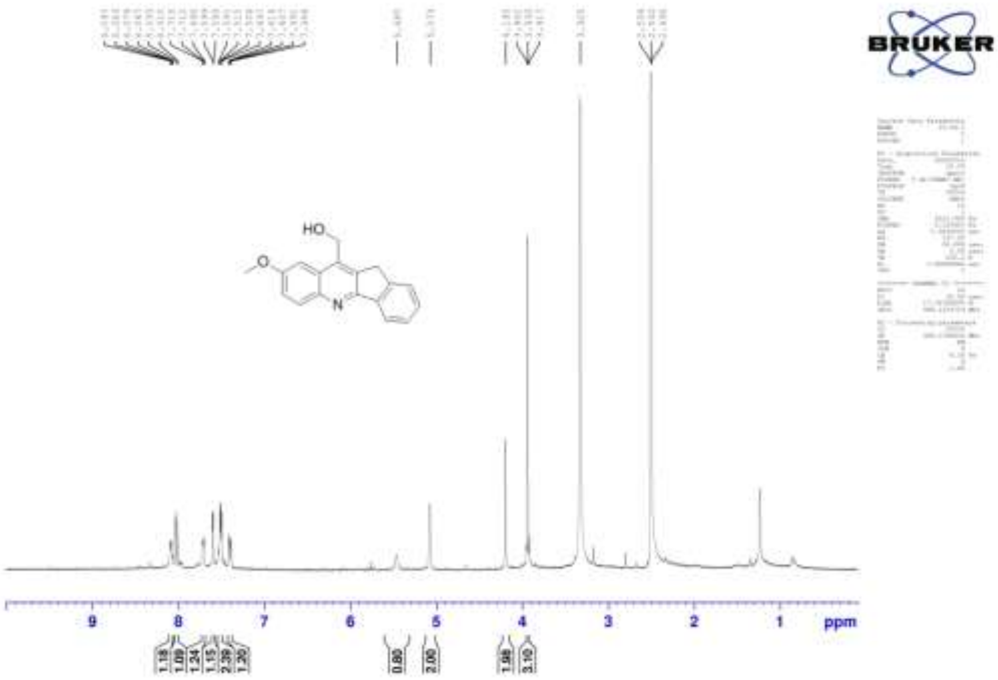
BRUKER
HO
N
9
8
7
6
5
4
3
2
1
ppm
1.18
1.09
1.24
1.15
2.29
1.26
0.80
2.00
1.98
3.10

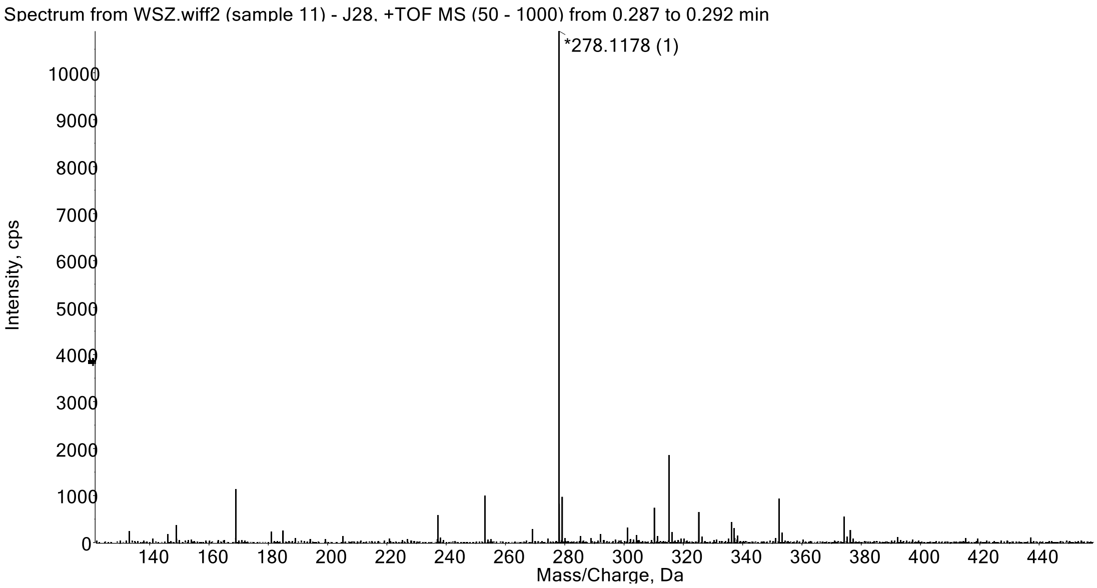
Spectrum from WSZ.wiff2 (sample 11) - J28, +TOF MS (50 - 1000) from 0.287 to 0.292 min
*278.1178 (1)
Intensity, cps
10000
9000
8000
7000
6000
5000
4000
3000
2000
1000
0
140
160
180
200
220
240
260
280
300
320
340
360
380
400
420
440
Mass/Charge, Da

Compound **J2**

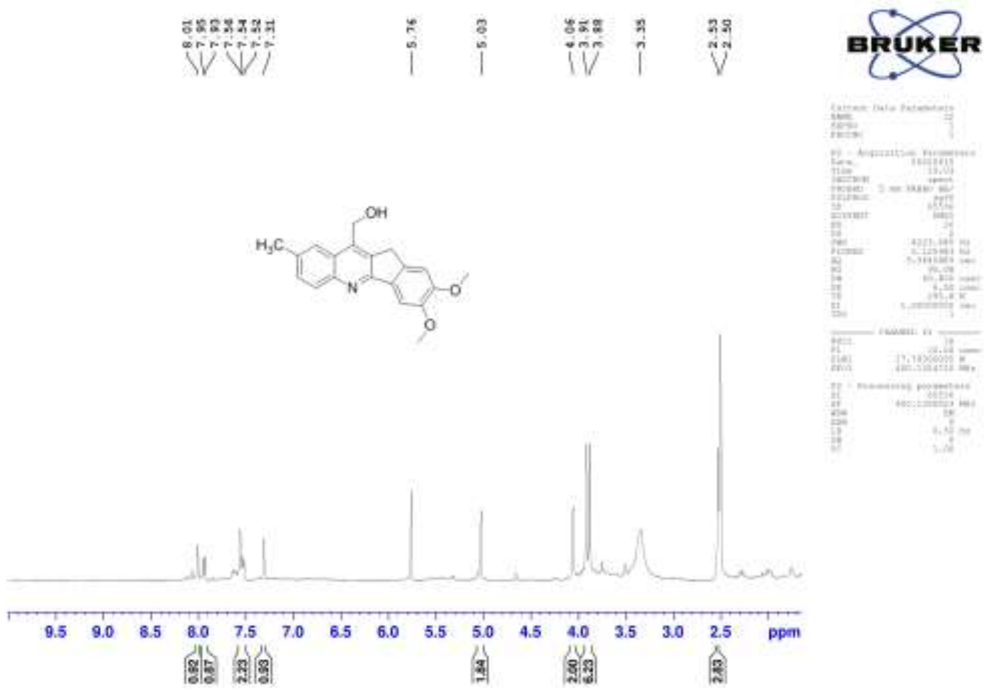

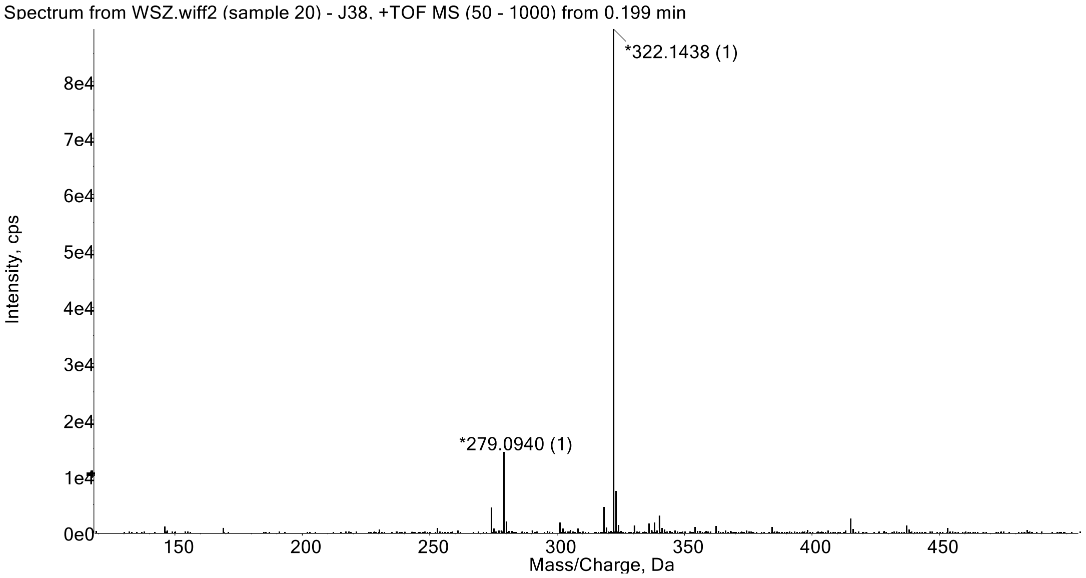
Spectrum from WSZ.wiff2 (sample 20) - J38, +TOF MS (50 - 1000) from 0.199 min
*322.1438 (1)
*279.0940 (1)
Intensity, cps
Mass/Charge, Da
